# The Digital Stressors Scale: Development and Validation of a New Survey Instrument to Measure Digital Stress Perceptions in the Workplace Context

**DOI:** 10.3389/fpsyg.2021.607598

**Published:** 2021-03-12

**Authors:** Thomas Fischer, Martin Reuter, René Riedl

**Affiliations:** ^1^Institute of Digital Business, Johannes Kepler University Linz, Linz, Austria; ^2^Department of Psychology, University of Bonn, Bonn, Germany; ^3^Digital Business, School of Business and Management, University of Applied Sciences Upper Austria, Steyr, Austria; ^4^Institute of Business Informatics – Information Engineering, Johannes Kepler University Linz, Linz, Austria

**Keywords:** digital stress, stressors, questionnaire, measurement scale, validation, technostress, work stress, digitalization

## Abstract

This article reports on the development of an instrument to measure the perceived stress that results from the use and ubiquity of digital technology in the workplace. Based upon a contemporary understanding of stress and a set of stressors that is a substantial update to existing scales, the Digital Stressors Scale (DSS) advances the measurement of digital stress. Initially, 138 items were constructed for the instrument and grouped into a set of 15 digital stressors. Based on a sample of *N* = 1,998 online questionnaires completed by individuals representative of the US employed population, the scale was refined using exploratory factor analyses (EFA) and PLS-SEM. The resulting and final scale consists of ten stressor categories reflective of one higher-order construct and a total of 50 items. Through a nomological network that includes important outcome variables of digital stress (emotional exhaustion, innovation climate, job satisfaction, user satisfaction) it was then demonstrated that the DSS provides substantial explanatory power, particularly related to emotional exhaustion and user satisfaction. Thus, the DSS constitutes a state-of-the-art self-report instrument to measure the extent of distress appraisal related to digital technologies in the workplace and helps to explain further how and why information and communication technologies can lead to adverse outcomes in individuals, thereby providing the starting point for job related organizational interventions.

## Introduction

We are experiencing an unprecedented prevalence of information and communication technologies (ICT) in our daily lives. Currently, almost 60% of the global population have access to the internet (Internetworldstats, 2020) and an estimated 1.4 billion smartphones are shipped every year (IDC, 2019). However, ICT are not just a tool for the individual but also an important asset for many organizations with global spending on enterprise software alone reaching an estimated 3.8 trillion dollars worldwide in 2019 (Gartner, 2019). The introduction of ICT in the organizational context has also led to an extensive line of research on the impact of these technologies. Brynjolfsson (1996), for example, highlighted that the cost of investments into ICT will yield a return about three times as high in customer benefits. Similarly, most research found that investments into information technology will reap great benefits for organizations (e.g., for automation purposes, Mukhopadhyay et al., 1997; or by enabling new sourcing strategies, Schneider and Sunyaev, 2016), but also for individuals (e.g., in the form of health information technology, Buntin et al., 2011, or smart home technologies, Wilson et al., 2017).

Despite such benefits, the use of ICT also has a “dark side,” including digital stress. For example, several studies found that unexpected ICT behavior can strain individual physiological wellbeing (e.g., computer breakdowns lead to elevated levels of adrenaline excretion and mental fatigue, Riedl, 2013). In recent years, it was also found that digital stress may negatively affect outcome variables directly related to information systems success (e.g., usage intention or user satisfaction, Fuglseth and Sørebø, 2014), individual performance at work (e.g., technology-supported performance, Ragu-Nathan et al., 2008), or emotional exhaustion (Ayyagari et al., 2011; Tams et al., 2014).

A growing stream of research, therefore, now also focuses on digital stress as a side effect of the increasing economical and societal prevalence of ICT (see, for example, reviews by Fischer and Riedl, 2017; Agogo et al., 2018; La Torre et al., 2019; Benzari et al., 2020), which is in line with earlier calls for further investigations into the intangible benefits and costs of ICT (e.g., Brynjolfsson and Hitt, 2000). Within this research stream, the main focus is on the use of ICT at work (Agogo et al., 2018; La Torre et al., 2019) and the main data collection methods are self-report questionnaires (Fischer and Riedl, 2017). This fact can be explained by the dominant role of situational appraisal in the stress process (e.g., Lazarus and Folkman, 1984; Cummings and Cooper, 1998), which necessitates the use of introspective measures. In particular, in the context of the current wave of digitalization, calls have been made for further inquiries into how people perceive the new digital environment and its impact on the individual, organizations, and society (Legner et al., 2017; Parviainen et al., 2017).

Here, we report on a new self-report measure for the assessment of perceived digital stress, as the speed at which our technological environment changes also demands a regular update of related measurement techniques. In particular, we seek to provide an update to an established measurement scale, the Technostress Creators (TSC) by Ragu-Nathan et al. (2008), by answering the following main research question: “*Which stressors should be part of a contemporary scale that measures digital stress and how can they be operationalized?*”

## Materials and Methods

The development and validation of the survey instrument is based on established frameworks (Moore and Benbasat, 1991; Netemeyer et al., 2003; MacKenzie et al., 2011), which include the following steps: (1) conceptualization of the focal construct of digital stress, (2) the development of the survey measure including the generation of items and card sorting to test initially the dimensionality of the construct, (3) specification of the nomological network for the construct, and (4) validation of the instrument including data collection procedures, assessment of psychometric properties, and comparison with an existing instrument.

### Conceptualization of “Digital Stress”

Changes in the technological environment have also changed the research on digital stress and in particular the conceptualization of this phenomenon. To clarify the understanding of digital stress that is used as a basis for the development of a new questionnaire, its main components are (1) stress, and (2) digital technologies (also briefly referred to as ICT). These are first clarified before definitions of digital stress as a construct are compared.

#### Stress

Originally understood as a bodily reaction to taxing stimuli (Selye, 1956), the understanding of this phenomenon has changed significantly and the modern approach to the conceptualization of stress entails a transaction between the individual and the environment (i.e., stress as a process, Lazarus and Folkman, 1984). Importantly, while the original understanding of stress did not consider perception to be of importance to the occurrence of adverse outcomes on the individual level, in the process-based understanding, perception (situational appraisal) plays a dominant role. To emphasize the role of perception further, we also consider the related concept of a “stressor.” Stressors are demands that force a variable outside of its range of stability (Cummings and Cooper, 1998). For example, unusual task demands might force an individual to handle an uncomfortably high amount of work, or system malfunctions might create interruptions in an individual’s usual workflow. To be stressors (i.e., a source of individual distress), these demands must first be perceived by the individual and then be appraised as detrimental to the individual’s well-being (e.g., a higher workload could also be perceived as beneficial if the individual is in need of higher levels of stimulation).

#### Technology

Rather than referring to all types of man-made inventions at this point (e.g., technologies such as wheels or written language), the focus is on digital technologies for the purpose of information management in a wider sense (e.g., capture, storage, retrieval, and analysis purposes). Such a conceptualization was, for example, the basis for the seminal study on digital stress by Ayyagari et al. (2011), who introduced a clear distinction between information and communication technologies and technologies found on the shop-floor (e.g., technologies for manufacturing automation). Digital technologies include, amongst others, mobile technologies (e.g., cell phones), network technologies (e.g., the Internet), communication technologies (e.g., e-mail), and generic application technologies (e.g., for word processing).

#### Digital Stress

The more widespread term used for digital stress in previous research is so-called “technostress,” which was coined by Brod (1982, p. 754) and refers to “… a condition resulting from the inability of an individual or organization to adapt to the introduction and operation of new technology.” Proposing a definition that is compatible with the transactional paradigm of stress, Ragu-Nathan et al. (2008, pp. 417-418) opted for a more general definition of the phenomenon, which is still widely used today and describes digital stress as “[a] phenomenon of stress experienced by end users in organizations as a result of their use of [ICT].” Riedl (2013, p. 18) more recently added that not only direct interaction, but also “… perceptions, emotions, and thoughts regarding the implementation of ICT in organizations and its pervasiveness in society in general” should be considered when assessing the stress potential of ICT. This addendum is also adopted here, as it helps to explain why potential future developments (e.g., the threat of job loss due to automation) could also lead to distress appraisal.

#### Dimensionality

Previous conceptualizations of digital stress indicate that it is a latent construct, usually composed of a multitude of stressors (Agogo et al., 2018). For example, Ayyagari et al. (2011) included six technology characteristics (i.e., usefulness, complexity, reliability, presenteeism, anonymity, pace of change) and Ragu-Nathan et al. (2008) used a set of five stressors that are reflective of digital stress (i.e., overload, invasion, complexity, insecurity, uncertainty). In both of these cases, there is a strong link to previous research in the wider context of organizational stress, with Ragu-Nathan et al. (2008) adapting popular work stressors (e.g., work overload becoming “techno-overload”) for their measurement scale and Ayyagari et al. (2011) linking technology characteristics (e.g., unreliability) to work stressors such as work overload. Hence, previous conceptualizations of work stress are an important basis for the conceptualization of digital stress at this point (e.g., Ivancevich and Matteson, 1980; Kahn and Byosiere, 1992; Williams and Cooper, 1998). In addition, there are stressors that are specific to digital technology (i.e., Privacy, Security, Unreliability, and Usefulness in the case of this study), which were consequently added to form a preliminary list of 15 ICT-related stressor categories (please refer to Section 1 in the Supplementary Material for further details):

1. *Boredom*. ICT can lead to boredom if more and more parts of an individual’s job are machine-paced and tasks that may be of importance to the employee are pushed towards automation (e.g., Stock, 2015).
2. *Complexity*. If ICT are not easily understood by individuals (e.g., software being hard to use, Al-Fudail and Mellar, 2008) this may be an important deterrent from work.
3. *Conflicts*. In some instances ICT can contribute to the blurring of boundaries between important life domains (e.g., work and home), referred to as the invasive property of technology (e.g., Ragu-Nathan et al., 2008).
4. *Control* (lack of). ICT can also limit the job autonomy of individuals and therefore reduce the degree of control that individuals have over their workday (e.g., Jones, 1999; Poole and Denny, 2001).
5. *Costs*. The use of ICT in the work context often involves a significant level of costs (e.g., Sahin and Coklar, 2009), though from an employee’s point of view costs is mostly reflected in time and cognitive effort.
6. *Insecurity*. ICT can cause a fear of unemployment (e.g., Sahin and Coklar, 2009; Frey and Osborne, 2017) as it is not certain which tasks and skills will be subject to automation in the future.
7. *Involvement* (lack of). Earlier research into the success of ICT (e.g., in terms of user satisfaction) found that the involvement of individuals in decision processes related to technological change (e.g., system design choices or purchase decisions) can be critical (e.g., McKeen et al., 1994).
8. *Overload*. External demands exceeding a desired level of stimulation (overload) in the form of work overload or information and communication overload are intensified through the use of ICT (e.g., Ayyagari et al., 2011; Barley et al., 2011; Galluch et al., 2015).
9. *Privacy Invasion*. The prospect of interactions with ICT being tracked is of major concern for many individuals and has also sparked an extensive stream of research (e.g., Bélanger and Crossler, 2011; Smith et al., 2011).
10. *Role Stress*. ICT can also contribute to higher levels of job-related ambiguity, as individuals are faced with a variety of demands that often compete for attention (e.g., Ayyagari et al., 2011; Schellhammer and Haines, 2013; Galluch et al., 2015).
11. *Safety*. There are many outside threats (i.e., outside of an organization) to the safety of work-related ICT, which can lead to stressful effects for the individual. In particular, many knowledge workers have to deal with potentially harmful programs (e.g., downloads that could include malicious code) that demand additional attention and not only threaten the individual, but also the organization (e.g., loss of company secrets) (e.g., Burke, 2009; D’Arcy et al., 2014; Hwang and Cha, 2018).
12. *Social Environment*. The characteristics of ICT and in particular communication technologies (e.g., e-mail) can also create unwanted norms and expectations that individuals have to deal with and may deviate from the actual desires of an individual (e.g., not wanting to communicate constantly) (e.g., Sahin and Coklar, 2009; Maier et al., 2015; Cao and Sun, 2018).
13. *Technical Support* (lack of). Not only do we consider stressful demands caused or mediated by ICT, but also the lack of resources to deal with such demands (e.g., inadequate technical support being in itself a source of distress, Ogan and Chung, 2002; Voakes et al., 2003; Al-Fudail and Mellar, 2008).
14. *Unreliability*. It can be highly stressful for individuals if ICT do not behave in an expected fashion, such as when response times are long or when a system breakdown occurs (e.g., Boucsein, 2009; Riedl et al., 2012).
15. *Usefulness* (lack of). Next to low levels of ease-of-use (i.e., high technology complexity), a lack of usefulness (Davis, 1989) is also considered to be a substantial digital stressor.

### Measure Development

#### Item Generation

Item statements for each of the 15 initial stressor categories were formulated independently by the first and third authors of this article and checked by the second author (e.g., phrasing and cognitive effort involved; items were reformulated where necessary), which led to an initial pool of 138 items (please refer to Section 2 in the Supplementary Material for a list of all items). In addition, as none of the authors is an English native speaker, the items were translated into their native tongue (i.e., German) based on their intended meaning and then translated back into English by a professional translator who was only involved for this purpose in the research project. The original English version was then compared with the translated version by the English native speaker and rated for content similarity, which was then the basis for corrections (see, for example, Eremenco et al., 2005 for a comparable approach).

#### Card Sorting

In line with the recommendations by MacKenzie et al. (2011), the dimensionality of the 138 items and 15 initial stressor categories as representations of digital stressors in the organizational context was initially assessed before the collection of survey data. More specifically, through two rounds of card sorting (five individuals in each round, blend of professionals and students), it was assessed whether these 15 stressor categories adequately represent the dimensionality of digital stress. In particular, we ascertained (i) whether they are in themselves crucial to the assessment of digital stressors and (ii) whether they are sufficiently distinct from each other. Based on a methodology applied by Moore and Benbasat (1991), separate rounds of open sorting (i.e., participants defined stressor categories themselves) and closed sorting (i.e., participants assigned statements to predefined stressor categories) were conducted. The closed sorting round revealed particular problems related to internal consistency of the initial stressor category *Costs* and based on the two open categories (“Not clear” and “Does not fit into any group”) items were flagged as potential candidates for removal during the measurement model evaluation stage (please refer to Section 3 in the Supplementary Material for further details on the Card Sorting procedure).

### Nomological Network

To assess the construct validity of the proposed instrument (Cronbach and Meehl, 1955), a nomological network with constructs known to have a relationship with digital stressors was established (MacKenzie et al., 2011). If digital stressors are actually measured, comparable patterns to those found in previous (technostress) research should emerge (e.g., Coltman et al., 2008, also refer to criterion variables in this context). The structure of the resulting nomological network is based on frameworks used frequently in research on digital stress (e.g., Ayyagari et al., 2011; Adam et al., 2017; Agogo et al., 2018). Common to these frameworks is a set of stimuli appraised as stressors, which then leads to detrimental outcomes (i.e., strains).

Based on evidence by Sarabadani et al. (2018), who analyzed ten years of applications of a technostress measurement instrument published in 2008, we identified important antecedents and outcomes of digital stress. This set includes (1) emotional exhaustion due to work as an outcome that is reflective of individual well-being at work and potentially indicative of long term consequences (e.g., health-related absences, Bakker et al., 2003), (2) the organizational climate for innovation, which is reflective of the perception that innovative behavior is supported within the organization, with innovation being crucial to organizational success (e.g., Wang and Wang, 2012), (3) job satisfaction as an outcome that is reflective of the work-related well-being of the individual, and (4) user satisfaction as an outcome that is reflective of the success of ICT employed at work.

#### Emotional Exhaustion

Next to cynicism and professional efficacy, emotional exhaustion is a common component of scales that measure symptoms of burnout and has been referred to as the stress dimension of burnout (Maslach et al., 2001, p. 403). More specifically, Maslach and Jackson (1981, p. 101) define it as “feelings of being emotionally overextended and exhausted by one’s work.” In line with previous research on digital stress (e.g., Ayyagari et al., 2011; Tams et al., 2014), it is expected that digital stressors will be positively related to emotional exhaustion.

#### Innovation Climate

Thus far, “…[a climate that] provide[s] support for innovation, encourage[s] communication, encourage[s] new ideas, and promote[s] supportive relationships among employees…” (Tarafdar et al., 2010, p. 315) has mainly been regarded as an inhibitor of digital stress (e.g., Ragu-Nathan et al., 2008; Tarafdar et al., 2015). It is argued here though that the presence of substantial stressors can reduce the perception of an organizational environment being conducive to innovative behavior (e.g., Clercq et al., 2014) Hence, it is expected that digital stressors will be negatively related to innovation climate.

#### Job Satisfaction

Previous research on digital stress has also found that job satisfaction, which can be defined as “a pleasurable or positive emotional state resulting from the appraisal of one’s job or job experiences” (Locke, 1976, p. 1300) can be negatively affected by digital stressors (e.g., Ragu-Nathan et al., 2008; Califf et al., 2015). In addition, reduced job satisfaction can be indicative of further long-term consequences of stress, such as individual turnover intention (e.g., Van Dick et al., 2004).

#### User Satisfaction

Both user satisfaction and job satisfaction are among the most important outcome variables in information systems research (e.g., Petter et al., 2008; Morris and Venkatesh, 2010), organization science (e.g., Bailey and Pearson, 1983; Wright and Bonett, 2007), and organizational psychology (e.g., Judge et al., 2001). Bhattacherjee (2001, p. 359) defines user satisfaction as “users’ affect with (feelings about) prior [digital technology] use” and it has been established in prior studies that ICT-related stressors can negatively impact this outcome variable (e.g., Tarafdar et al., 2010; Fuglseth and Sørebø, 2014).

**FIGURE 1:**
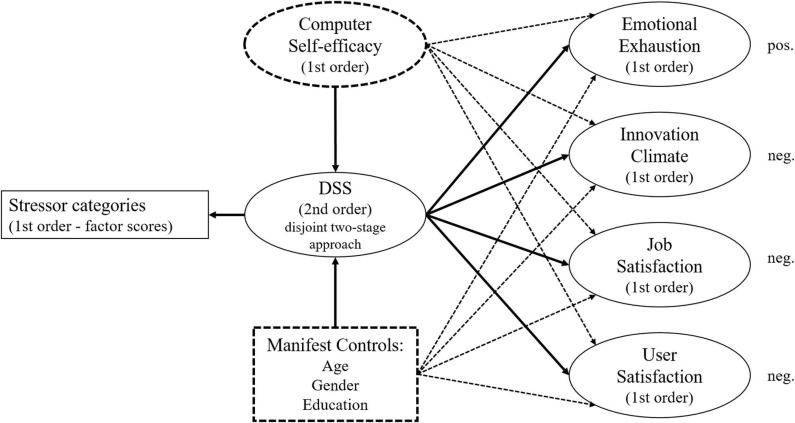
Nomological network for scale validation.

In addition to these criterion variables, a set of *control variables* is also included in the nomological network that have frequently been part of digital stress investigations. More specifically, individual characteristics including age, gender, highest level of education, and computer self-efficacy were measured. In line with previous studies, it is expected that age will be negatively related to digital stress such that younger individuals will experience higher levels of digital stress (Ragu-Nathan et al., 2008; Tarafdar et al., 2011; Hauk et al., 2019). Note that some studies also report a positive relationship between age and digital stress. However, these studies typically focus on a narrow facet of digital stress, and not on a more global construct (e.g., Tams et al., 2014, 2018 focus on interruption-based stress during computer work). For gender, it is expected that men will experience higher levels of digital stress than women (e.g., Tarafdar et al., 2011; Riedl et al., 2013). For education, a negative relationship with digital stress is expected, such that individuals with a higher level of education will experience lower levels of digital stress than individuals with a lower level of education (e.g., Tarafdar et al., 2011). Finally, computer self-efficacy is included as a control variable, which refers to the “…judgment of one’s capability to use a computer” (Compeau and Higgins, 1995, p. 192). In line with existing research (e.g., Shu et al., 2011), it is expected that individuals with high levels of computer self-efficacy will experience lower levels of digital stress as compared with individuals with lower levels of computer self-efficacy. The research model that is the basis for scale validation is summarized in Figure 1, with relationships for control purposes only being indicated by a dashed line and control variables being indicated by a dashed border. It is also highlighted that the Digital Stressors Scale (hereafter DSS) will be estimated as a higher-order construct, with the scores of the lower-order constructs (i.e., stressor categories) being used as indicators, following the disjointed two-stage approach as outlined by Sarstedt et al. (2019).

### Data Collection

#### Measures

Aside from the new measurement instrument, only established scales were used to collect data on the outcome and control constructs in the research model (see Figure 1). For emotional exhaustion, the corresponding five-item sub-scale in the Burnout Inventory by Maslach and Jackson (1981) was used (e.g., “I feel emotionally drained from my work”). For innovation climate, the five-item scale by Tarafdar et al. (2010) was applied (e.g., “We have a very open communications environment”). For job satisfaction, the three-item scale by Ragu-Nathan et al. (2008) was applied (e.g., “I like doing the things I do at work”). For user satisfaction, the four-item scale by Bhattacherjee (2001) was applied with a 7-point Likert scale with adjective pairs (e.g., “How do you feel about your overall experience of utilizing ICT in connection with your work tasks?” with answers ranging from 1 = very dissatisfied to 7 = very satisfied). For all other constructs, a 7-point Likert scale was consistently used ranging from 1 – “strongly disagree” to 7 – “strongly agree.”

Of the controls, only computer self-efficacy was a latent variable, which was measured using the 10-item instrument by Compeau and Higgins (1995) (e.g., “I could complete my tasks using new ICTs if there was no one around to tell me what to do as I go”) and a 7-point Likert scale (from 1 – “not at all imaginable” to 1 – “completely imaginable”). There were three options for gender (1 = male, 2 = female, and prefer not to say; “prefer not to say” with the latter treated as missing data), for age, participants were asked to indicate their year of birth and for educational attainment, all of the single-choice options were based on a classification system used by the US Bureau of Labor Statistics (10 categories, plus other, plus prefer not to say; see Table 1 below for the specific categories, Brundage, 2017).

**TABLE 1:** Overview of sample characteristics.

	**Full sample**	**Sample 1: Measurement model evaluation**	**Sample 2: Structural model evaluation**	**US census**
*Sample size*	*N* = 1,998	*N* = 1,016	*N* = 982	-
*Age*				
Median	39	38	39	42.2
Standard deviation	14.99	14.95	15.03	-
*Gender*				
Female	876 (43.8%)	456 (44.9%)	420 (42.8%)	46.9%
Male	1,122 (56.2%)	560 (55.1%)	562 (57.2%)	-
*Educational Attainment*				
Less than a highschool diploma	22 (1.4%)	12 (1.2%)	10 (1.0%)	8%
Highschool diploma	220 (11.0%)	106 (10.4%)	114 (11.6%)	26%
Some college (no degree)	358 (17.9%)	196 (19.3%)	162 (16.5%)	16%
Associate’s degree (occupational)	101 (5.1%)	52 (5.1%)	49 (5.0%)	5%
Associate’s degree (academic)	126 (6.3%)	64 (6.3%)	62 (6.3%)	6%
Bachelor’s degree	748 (37.4%)	366 (36.0%)	382 (38.9%)	24%
Master’s degree	316 (15.8%)	172 (16.9%)	144 (14.7%)	11%
Professional degree	49 (2.5%)	22 (2.2%)	27 (2.7%)	2%
Doctoral degree	52 (2.6%)	25 (2.5%)	27 (2.7%)	2%
Other	6 (0.3%)	1 (0.1%)	5 (0.5%)	-

To assess the convergent validity of the DSS, an existing instrument to measure digital stress was also included, the TSC scale by Ragu-Nathan et al. (2008). This scale’s 23 items were measured using a 7-point Likert scale ranging from 1 – “strongly disagree” to 7 – “strongly agree.”

#### Online Survey

Data were collected through a market research company^1^ from October 26 to November 8, 2018. The target population of the survey were employed individuals from the United States. All individuals who did not fulfill this criterion were excluded from participation. In addition to the survey items, two engagement checks that instructed participants to choose one specific option on the provided scale were included.

#### Sample Characteristics

The initial sample amounted to *N* = 3,358 completed questionnaires, which were then subject to a rigorous screening procedure to ensure the quality of the data (this was necessary due to the length of the questionnaire and the repetitiveness of the items for the new instrument) (Meade and Craig, 2012; DeSimone et al., 2015). Speeders were excluded (i.e., individuals with completion times of less than 10 min, the average for all *N* = 3,358 was about 25 min; *N* = 1,048 were excluded based on this criterion) as well as individuals who missed at least one of the engagement checks (*N* = 520). To ensure further the quality of the data, questionnaires containing a large share of missing data (i.e., more than 10% of items missing, *N* = 886) and/or showed low levels of engagement (i.e., a standard deviation of less than .50 on all continuous scales, *N* = 103) were also excluded. The final sample is *N* = 1,998 completed questionnaires for further analyses (please note that the listed exclusion criteria are not mutually exclusive and hence are overlapping, for example in the case of speeders and missing data).

The data were then split randomly into two sub-samples, one for the evaluation of the measurement model and one for the evaluation of the structural model (MacKenzie et al., 2011). The characteristics of these samples are displayed in Table 1 and are also compared to the US census, where data were available (Brundage, 2017; United States Department of Labor - Bureau of Labor Statistics., 2018). It can be observed that overall the samples are slightly younger, contain more men and show a higher educational attainment (e.g., more individuals with a bachelor’s degree and fewer individuals with only a highschool diploma) than the US average, which has to be kept in mind when interpreting the results of the analyses.

### Data Analysis

For step four in the scale development process (i.e., validation), a number of data analysis procedures are necessary, which are mainly used to establish the reliability and validity of the new instrument. In line with psychological and social science practices, the 7-point Likert scales data were treated as interval-scaled data (e.g., Norman, 2010; Wu and Leung, 2017).

The analyses were performed in several phases, as it was likely that the indicators that are part of the instrument would form a higher-order construct. For each level in this higher-order construct (i.e., from indicators to lower-order constructs, from lower-order to higher-order constructs), reliability and validity metrics were first assessed to guarantee internal consistency (initially without any relationships to external variables). Second, the relationships with criterion variables were tested (for further details, please refer to the Confirmatory Composite Analysis proposed by Hair et al., 2020).

In each phase, the directionality of the relationship between indicators and the higher-order construct had to be defined first (i.e., reflective if indicators are manifestations of a common construct or formative if they form the construct; Jarvis et al., 2003). For the first level (i.e., indicators to lower-order constructs), we followed Ragu-Nathan et al. (2008) and hence used reflective specification. We then initially conducted a series of exploratory factor analyses (EFA) as well as parallel analyses and a Velicer’s MAP test (O‘Connor, 2000) to develop insight into the dimensionality of the DSS further. For the resulting factors, we then followed the steps recommended by Hair et al. (2019) to ensure the quality of the measurement model involving the resulting 1st order constructs:

• In line with recommendations by MacKenzie et al. (2011), the validity of the new construct (*construct validity*) can be indicated by its (i) content validity (initially established based on the literature review that was used to create the items and initial factors), (ii) convergent validity (indicators’ load on their respective construct; average variance extracted (AVE) is used as the main indicator, as well as extent and significance of loadings of an indicator), (iii) discriminant validity (smallest possible overlap with other constructs; Fornell-Larcker criterion (Fornell and Larcker, 1981), and heterotrait-monotrait ratio of correlations (HTMT, Henseler et al., 2015) were used as indicators), and its (iv) nomological validity (based on existing knowledge, the construct is expected to show relationships with other constructs). Content, convergent, and discriminant validity were tested in both phases, while nomological validity was tested using the highest-level construct (i.e., the 2nd order construct in our case).
• For the *reliability* of the constructs, three indicators are used, namely Cronbach’s Alpha (α - most conservative measure and therefore the lower bound), Composite reliability (ρc - higher bound) and the Rho Alpha (ρA) (Hair et al., 2019).

These indicators were used to create a set of 1st order constructs that showed sufficient reliability and convergent validity (constructs that did not fulfill these minimum requirements were removed). The indicators were then used to form a 2nd order construct, and the model specification (reflective vs. formative) was investigated using the criteria proposed by Coltman et al. (2008). For the 2nd order construct, reliability and validity were assessed again, including discriminant validity in relation to the four criterion variables.

These steps concluded the *evaluation of the measurement model*. Thus, the new instrument as well as other constructs included in this investigation showed sufficient internal consistency and were also sufficiently conceptually different from each other.

The nomological validity of the new instrument was then tested during the *structural model evaluation*, when its relationships with the four criterion variables and the control variables were tested. For this purpose, a number of regression models were estimated. In addition, the same procedures were implemented using the existing TSC instrument to make possible a direct comparison with our DSS. Moreover, we confirmed that the relationships with other variables found with TSC could also be found with the new instrument.

The psychometric properties of the DSS were predominantly assessed using PLS-SEM (using SmartPLS 3 v. 3.2.8) due to some of the benefits of this analytical approach as compared with covariance-based SEM (CB-SEM). According to recent evidence presented by Hair et al. (2019), PLS-SEM is more robust against non-normality of data and is particularly suited for formative models (formative models are also feasible in CB-SEM using MIMIC models, Diamantopoulos, 2011, though such models can lead to results that are not theoretically sound, Hair et al., 2019). Although CB-SEM is the prime method to investigate higher order constructs, it has also been shown recently that PLS-SEM supports models with higher order constructs (Sarstedt et al., 2019).

## Results

### Measurement Model Evaluation

To evaluate the measurement model, the factor structure for the DSS first had to be checked for its internal consistency, convergent validity, and discriminant validity (Hair et al., 2019; Sarstedt et al., 2019). For the related analyses, the first sub-sample was used and if not reported otherwise, 5,000 iterations were applied in each run.

#### Exploratory Factor Analysis (EFA)

As the open sorting task led to further stressor categories that could be considered, the factor structure was further checked employing an EFA in SPSS v. 26 (extraction: principal axis; rotation: promax) with all 138 items as input. With no factor restrictions, this approach resulted in 17 factors with an Eigenvalue above 1 (KMO:0.985, Bartlett’s:0.000, explained variance: 54.70%) (please refer to Section 4.1 in the Supplementary Material for the full pattern matrix). When restricting the factor extraction to 15 and 20 factors respectively (15 original stressor categories and five categories considered from the open sorting), the results only changed marginally (15 factors – KMO:0.985, Bartlett’s:0.000, explained variance: 53.86%; 20 factors – KMO:0.985, Bartlett’s:0.000, explained variance: 55.87%). Hence, there is potential for factor reduction, which is further indicated by the first extracted factor explaining 36.92% of indicator variance and eight factors being sufficient to explain a majority of indicator variance (i.e., the cumulative explained variance of the first eight factors with the largest share of explained variance is 50.25%). In line with recommendations by O‘Connor (2000), we also ran two additional analyses to get an idea of the number of factors in the final solution. We conducted a parallel analysis and a Velicer’s MAP test (MAP), using the syntax for SPSS provided by O‘Connor (2000). We ran the parallel analysis syntax and compared the randomly generated Eigenvalues with the Eigenvalues created by a principal component analysis (PCA) without rotation. In this procedure, the PCA resulted in 17 factors with an Eigenvalue above 1, but only 7 of these factors had Eigenvalues larger than the respective factors randomly generated during parallel analysis, which indicates that this number of factors should be retained. We then also ran the MAP, which resulted in a recommendation of 12 factors that should be retained. Hence, both of these methods further substantiated the idea that a solution with 15 factors would not be realistic and we expected that the final factor solution would be within the range of 7 to 12 factors.

#### Set of 1st Order Constructs

The original 15 stressor categories were used initially for measurement model evaluation in SmartPLS with the goal of creating a set of lower order constructs that is internally consistent as indicated by reliability metrics, has sufficient convergent validity as indicated by the average variance explained (AVE) and, if possible at this stage (i.e., without the use of a higher-order construct), has sufficient discriminant validity as indicated by the Fornell-Larcker criterion and the HTMT (Hair et al., 2019; Sarstedt et al., 2019). This process involved the refinement of each stressor category (e.g., removal of indicators with low factor loadings and high cross-loadings), which was necessary as the 15 categories in their initial form did not meet the reliability and validity thresholds (see Tables 10 and 11 in Section 4.2 of the Supplementary Material).

First, after items were removed from these categories, they were used to form alternative categories (i.e., the five categories identified during the card sorting exercise) with the goal of building internally consistent categories, while also retaining as many items and categories as possible. This procedure was chosen as these additional categories overlapped with existing stressor categories. However, none of the additional categories emerged as a viable alternative (in terms of reliability and convergent validity) to the 15 existing categories without introducing additional ambiguity. As an example, based on the results of the open sorting procedure “Distraction through ICT” would include between 5 and 18 items, which are mostly part of the original category “Role stress,” yet also including items from “Social Environment” and “Safety.” Hence, creating this larger category “Distraction through ICT” would have threatened the internal consistency of other categories and therefore would have led to an overall less distinctive factor structure.

Second, the process was then repeated with the initial 15 categories, with the priority being the retention of categories rather than items (i.e., number of items per category was reduced before the elimination of a whole category was considered). This was a repetitive and hence exhausting process with a back and forth movement between the elimination of items and categories and an reintroduction of items and categories (e.g., when issues due to substantial cross-loadings were resolved, which then warranted an attempt to reintroduce a previously eliminated category). These two goals (i.e., trying to retain as many items and categories as possible, while also trying to create internally consistent categories) ultimately led to the elimination of five stressor categories (due to internal consistency issues). Hence, due to the iterative nature of this process and the involved challenges, further investigations into the factor structure of digital stressor categories and the validation of our final 10-factor structure are warranted.

The final factor structure, which fulfills all necessary criteria is presented in Table 2 (i.e., reliability metrics > 0.700, Nunnally and Bernstein, 1994; AVE > 0.500, MacKenzie et al., 2011) (please refer to Section 4.2 in the Supplementary Material for further details). For discriminant validity, the following criteria were applied: fulfillment of the Fornell-Larcker criterion (Fornell and Larcker, 1981) and HTMT < 0.900 (fulfilled in most cases) (Henseler et al., 2015). In the resulting factor structure, five of the original stressor categories had to be removed due to issues related to reliability and/or convergent validity. The resulting set of 1st order constructs was then used as the basis to test a model including a higher order construct for the DSS, as digital stress has previously also been mainly measured as a 2nd order construct (e.g., Ragu-Nathan et al., 2008; Sarabadani et al., 2018).

**TABLE 2:** Reliability and validity statistics for 1**st** order DSS constructs.

**Stressor categories**	**Cronbach’s α**	**ρA**	**Composite reliability ρc**	**Average Variance Extracted (AVE)**
I. Complexity	0.876	0.879	0.910	0.669
II. Conflicts	0.905	0.907	0.929	0.724
III. Insecurity	0.921	0.926	0.940	0.758
IV. Invasion	0.860	0.874	0.898	0.638
V. Overload	0.849	0.864	0.892	0.625
VI. Safety	0.847	0.851	0.891	0.622
VII. Social environment	0.795	0.805	0.859	0.549
VIII. Usefulness	0.838	0.857	0.885	0.609
IX. Technical support	0.873	0.873	0.908	0.663
X. Unreliability	0.838	0.845	0.885	0.608
**Criterion variables**				
A. Emotional exhaustion	0.887	0.889	0.918	0.691
B. Innovation climate	0.718	0.764	0.836	0.631
C. Job satisfaction	0.822	0.831	0.894	0.738
D. User satisfaction	0.923	0.929	0.946	0.813

The items included in the final ten stressor categories are listed below:

I. **Complexity**

1. I often find it too complicated to accomplish a task using the ICT that are available to me at work.
2. I often need more time than expected to accomplish a task using the ICT that are available to me at work.
3. I feel that the ICT that are available to me at work are too confusing.
4. I often do not find enough time to keep up with new functionalities of ICT at work.
5. It would take me too long to completely figure out how to use the ICT that are available to me at work.

II. **Conflicts**

1. I feel that my private life suffers due to ICT enabling work-related problems to reach me everywhere.
2. It is too hard for me to keep my private life and work life separated due to ICT.
3. ICT make it harder to create clear boundaries between my private life and work life.
4. My work-life balance suffers due to ICT.
5. The ubiquity of ICT disturbs my work-life balance.

III. **Insecurity**

1. I feel that my job position is threatened due to ICT.
2. I fear that I could be replaced at work due to the increasing standardization of work processes, which is enabled by ICT.
3. I cannot be optimistic about my long-term job security because of the threat of ICT automatization.
4. I fear that I could be replaced by machines.
5. I fear that digitalization will cost me my job.

IV. **Invasion (of Privacy)**

1. I fear that my use of ICT is less confidential than I would like to.
2. I fear that the information that I exchange using ICT is not as protected as I would like to.
3. I fear that malevolent outsiders (e.g., hackers) can easily copy my identity due to ICT.
4. My personal information is too easily accessible due to ICT.
5. I fear that my personal data can easily be stolen by others online.

V. **Overload**

1. Due to ICT I have too much to do.
2. Due to ICT I have a too large variety of different things to do at work.
3. ICT make it too easy for other individuals to send me additional work.
4. I never have any spare time, because my schedule is too tightly organized by ICT.
5. There is a constant surge of work-related information coming in through ICT that I just cannot keep up with.

VI. **Safety**

1. I have to worry too often, whether I might download malicious programs.
2. I have to worry too often, whether I might receive malicious e-mails.
3. I fear that hackers might get access to company secrets through a mistake of mine.
4. I feel anxious when I get an e-mail from somebody that I do not know as it could be a malevolent attack.
5. E-Mails whose sender I do not know make me nervous.

VII. **Social Environment**

1. Due to ICT I have too much to do with the problems of others.
2. I think that ICT generate too much of an expectation that I have to be reachable everywhere and at any time.
3. Too much time gets lost at work because of irrelevant communication with other people on social media.
4. I feel that ICT create unwanted social norms (e.g., the expectation that e-mails should be answered right away).
5. It is too hard to take a break from social interactions at work due to the communication possibilities of ICT.

VIII. **Technical Support**

1. I have to worry about ICT-related problems as our organization does not offer enough support for their removal.
2. In the case of ICT-related problems, it happens too often that there is not enough support available at work.
3. I think that it happens too often that technical support is not available when I need it.
4. I often have to wait for a long time because technical problems cannot be adequately solved in our organization.
5. I fear that a technical problem I have at work could not be solved by anyone else at work.

IX. **Usefulness**

1. I think that the demands of my work and the functions provided by the ICT I use do not fit sufficiently.
2. I think that I do not gain enough benefits from using the ICT that I am provided with at work for my tasks.
3. The ICT I use at work are full of too many functionalities that I never need.
4. It requires too many different systems to fulfill the tasks that I have to do during an average day at work.
5. I think that most of the ICT I am supplied with at work is not useful enough and I could work without it.

X. **Unreliability**

1. I think that I am too often confronted with unexpected behavior of the ICT I use at work (e.g., breakdowns or long response times).
2. I think that I lose too much time due to technical malfunctions.
3. I think that I spend too much time trying to fix technical malfunctions.
4. There is just too much of my time at work wasted coping with the unreliability of ICT.
5. The daily hassles with ICT (e.g., slow programs or unexpected behavior) are really bothering me.

#### Model Specification

**FIGURE 2:**
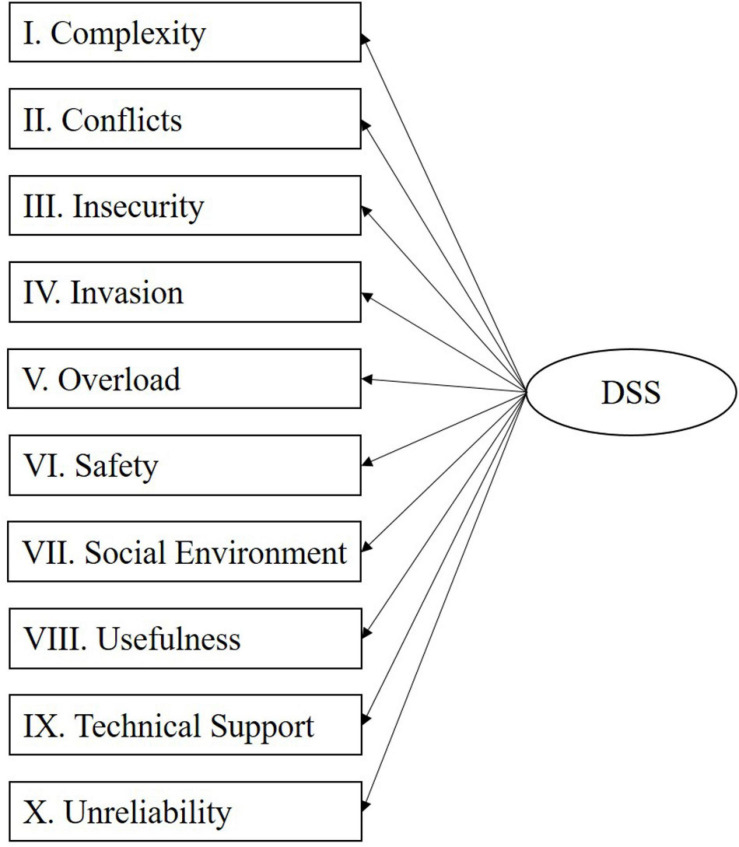
Reflective model specification.

**FIGURE 3:**
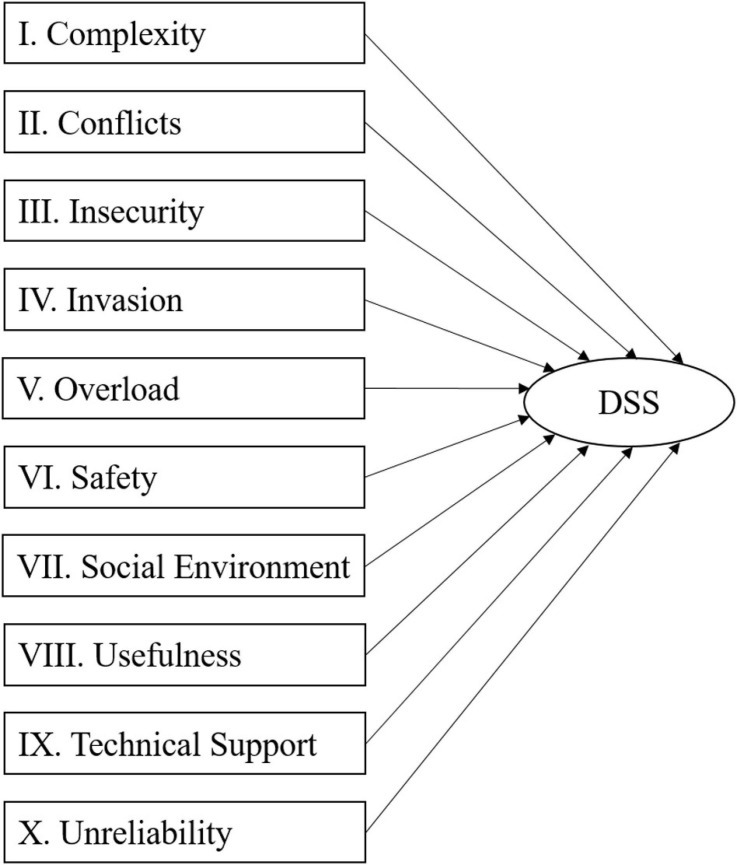
Formative model specification.

In line with previous conceptualizations of digital stress as a higher order construct (e.g., Ragu-Nathan et al., 2008), such a conceptualization was also tested for the new measurement scale. Support for a potential higher order construct can also be found in the correlation patterns of the 1st order constructs in the DSS, which range from 0.414 (Insecurity and Invasion) to.809 (Complexity and Unreliability). This is comparable with the correlations of the five 1st order constructs in an existing instrument (i.e., the TSC), which range from 0.357 (Invasion and Uncertainty) to.727 (Invasion and Overload). Regarding the relationships with outcome variables, in most cases, correlations with emotional exhaustion are positive (0.405 to.613). Further, correlations with innovation climate (-0.004 to -0.135; one exception with a correlation of.001), job satisfaction (-0.128 to -0.258), and user satisfaction (-0.199 to -0.409) are negative. Although a reflective specification was chosen for the 1st order constructs, it was further assessed whether the higher-order construct should be specified as a reflective or as a formative construct (Jarvis et al., 2003; MacKenzie et al., 2011). For this purpose, the six theoretical and empirical considerations proposed by Coltman et al. (2008) were applied to argue for a reflective or formative specification. Regarding the distinction between reflective and formative models, we refer to Kenny (2016), who distinguished them as follows: “A formative construct or composite refers to an index of a weighted sum of variables. In a formative construct, the indicators cause the construct, whereas in a more conventional latent variables, sometimes called reflective constructs, the indicators are caused by the latent variable.” This distinction is also in line with Jarvis et al. (2003) and MacKenzie et al. (2011) and we illustrate the reflective specification in Figure 2 and the formative specification in Figure 3 below (please refer to Section 4.3 in the Supplementary Material for further details).

In order to estimate the 2nd order construct, we followed the disjointed two-stage approach as outlined by Sarstedt et al. (2019), which involved first calculating a model in which all 1st order constructs are connected to the outcome variables. The resulting factor scores for the 1st order constructs were then used for a second model in which these factor scores served as indicators for the 2nd order construct.

Following Coltman et al.’s (2008) considerations led us to mixed results and therefore a more practical approach was chosen, and a reflective specification was directly compared with a formative specification. This step involved the estimation of both types of models and the comparison of the resulting path coefficients and the explained variance for the endogenous variables (see also Coltman et al., 2008 for a comparable approach). The results of this comparison can be found in Table 3. The patterns for the path coefficients (i.e., sign and significance of paths from the 2nd order construct to the criterion variables) are comparable, though the loadings (weights) for the 1st order constructs differ, as some of the weights in the formative specification are not significant. In addition, the difference in explained variance only ranges from 0.004 to 0.023, which is considered marginal at this point as it is generally expected that formative specifications explain a larger share of variance (Coltman et al., 2008). Therefore, as there is no clear indication for a formative specification, a reflective specification was chosen instead, in line with Ragu-Nathan et al. (2008). Nonetheless, as the results were mostly ambiguous, alternative specifications (e.g., 1st order reflective and 2nd order formative) should be further investigated in the future (see, for example, Sarstedt et al., 2019, p. 198) for an overview of all four main types of model specifications, combining reflective and formative specifications).

**TABLE 3:** Comparison of 2nd order DSS construct with reflective and formative specification.

**Criterion variable**	**Path coefficients, significance and explained variance**	
	**Reflective specification**	**Formative specification**
Emotional exhaustion	β = 0.645 (t = 32.303, *p* < 0.001),	β = 0.662 (t = 32.446, *p* < 0.001),
	R^2^ adj. = 0.415	R^2^ adj. = 0.438
Innovation climate	β = −0.092 (t = 2.633, *p* = 0.008),	β = −0.111 (t = 2.932, *p* = 0.003),
	R^2^ adj. = 0.007	R^2^ adj. = 0.011
Job satisfaction	β = −0.275 (t = 8.770, *p* < 0.001),	β = −0.288 (t = 8.990, *p* < 0.001),
	R^2^ adj. = 0.075	R^2^ adj. = 0.082
User satisfaction	β = −0.381 (t = 11.132, *p* < 0.001),	β = −0.403 (t = 11.324, *p* < 0.001),
	R^2^ adj. = 0.145	R^2^ adj. = 0.162
Path/Weight significance	Loadings all significant (*p* < 0.05)	Weights not all significant (*p* > 0.05 for Complexity, Insecurity, Overload, Safety, Technical Support)
		

#### 2nd Order Construct Model Assessment

Based on a reflective (1st order)/reflective (2nd order) specification (also referred to as a superordinate construct by Edwards (2001), or a Type I construct by Jarvis et al. (2003); as shown in Figure 2) reliability and validity metrics were then estimated again (Sarstedt et al., 2019). The results are displayed in Tables 4, 5, which indicate sufficient reliability (Cronbach’s α, ρA, and ρc > 0.700), sufficient convergent validity (AVE > 0.500), and sufficient discriminant validity (based on Fornell-Larcker criterion displayed in Table 4 and HTMT<0.900 or <0.850 as displayed in Table 5).

**TABLE 4:** Reliability and validity statistics for 2nd order DSS construct (reflective/reflective).

	**α**	**ρ*A***	**ρ*c***	***AVE***	***DSS***	***A***	***B***	***C***	***D***
DSS	0.944	0.953	0.953	0.669	*0*.*818*				
A. Emotional exhaustion	0.887	0.889	0.918	0.691	0.645	*0*.*831*			
B. Innovation climate	0.718	0.740	0.840	0.636	–0.092	–0.282	*0.798*		
C. Job satisfaction	0.822	0.840	0.894	0.738	–0.275	–0.500	0.518	*0.859*	
D. User satisfaction	0.923	0.932	0.946	0.813	–0.381	–0.357	0.396	0.462	*0.902*

*Values in italics in the right half of the table indicate the square root of the AVE, while values below indicate the inter-construct correlations for the latent variable scores.*

**TABLE 5:** Discriminant validity for 2nd order DSS construct based on HTMT.

	**DSS**	**Emotional exhaustion**	**Innovation climate**	**Job satisfaction**
Emotional exhaustion	0.697			
Innovation climate	0.111	0.357		
Job satisfaction	0.304	0.577	0.662	
User satisfaction	0.399	0.393	0.490	0.524

Further details of the resulting model specification can be found in Section 4.5 of the Supplementary Material. In addition, two alternative model specifications were also tested and the results are presented in Section 4.4 of the Supplementary Material (one 1st order construct including all items in Section 4.4.1 and the possibility of several 2nd order constructs in Section 4.4.2), though none of them emerged as a better alternative to the current model specification.

### Structural Model Evaluation

**FIGURE 4:**
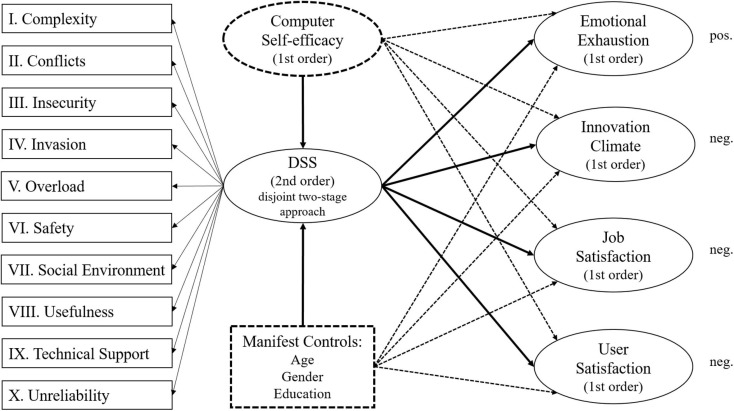
Structural model.

To evaluate the structural model, which also includes four control variables (i.e., age, gender, highest level of education, and computer self-efficacy), the second sub-sample was used and calculations in SmartPLS involved 5,000 iterations if not stated differently. To check initially whether the two sub-samples were comparable and that the selection of samples would not coincidentally lead to different results, the scores for each included latent variable were statistically compared using Mann-Whitney tests. As none of these tests approach statistical significance, it can be assumed that the results of model estimations with both samples will lead to comparable results. The model that was assessed at this stage is illustrated in Figure 4 below. Note that dashed variables and dashed lines indicate control variables and their relationships with outcome variables.

#### Explanatory Power

To assess construct validity, the relationship of the new instrument with the four criterion variables and a selection of control variables were estimated. In addition, the same models were also estimated with an alternative measure that is already established in research on digital stress (i.e., TSC scale, Ragu-Nathan et al., 2008). An indicator for their comparable scope, in addition to their items and dimensions, is the high correlation of their latent variable scores of.923 (i.e., correlation of DSS and TSC, *p* < 0.001, based on Spearman correlation). Four separate regression models were estimated at this point (DSS without controls and with controls, and TSC without controls and with controls; for further details please refer to Section 5 in the Supplementary Material). The main results related to nomological validity are presented in Table 6, which includes an assessment of the support for previously expected relationships between constructs based on significance (*p* values) and path coefficients (β values). The main results related to explanatory power are presented in Table 7, which includes the path coefficients and significance for each criterion variable as well as the explained variance (R^2^ adjusted) and effect size (f^2^) in the case of models with controls.

**TABLE 6:** Nomological validity assessment for DSS and TSC.

**Expected relationships**	**DSS**	**TSC**
DSS/TSC → Emotional exhaustion (pos.)	Supported β = 0.640, *p* < 0.001	Supported β = 0.597, *p* < 0.001
DSS/TSC → Innovation climate (neg.)	Supported β = −0.232, *p* < 0.001	Supported β = −0.216, *p* = 0.001
DSS/TSC → Job satisfaction (neg.)	Supported β = −0.274, *p* < 0.001	Supported β = −0.222, *p* < 0.001
DSS/TSC → User satisfaction (neg.)	Supported β = −0.438, *p* < 0.001	Supported β = −0.373, *p* < 0.001
Age → DSS/TSC (neg.)	Supported β = −0.122, *p* < 0.001	Supported β = −0.104, *p* = 0.159
Gender → DSS/TSC (neg. = higher for men)	Not supported β = −0.050, *p* = 0.107	Supported β = −0.064, *p* = 0.034
Education → DSS/TSC (neg.)	Not supported β = 0.046, *p* = 0.159	Not supported β = 0.034, *p* = 0.293
Computer Self-Efficacy → DSS/TSC (neg.)	Supported β = −0.164, *p* < 0.001	Supported β = −0.203, *p* = 0.010

**TABLE 7:** Path coefficients and effect sizes for DSS and TSC.

	**Emotional exhaustion**	**Innovation climate**	**Job satisfaction**	**User satisfaction**
DSS – main effects	β = 0.640 (t = 32.991,	β = −0.232 (t = 7.955,	β = −0.274 (t = 9.038,	β = −0.438 (t = 14.672,
	*p* < 0.001), R^2^ adj. = 0.409	*p* < 0.001), R^2^ adj. = 0.054	*p* < 0.001), R^2^ adj. = 0.075	*p* < 0.001), R^2^ adj. = 0.192
DSS - with controls	β = 0.636 (t = 31.112,	β = −0.204 (t = 5.939,	β = −0.238 (t = 7.348,	β = −0.421 (t = 13.985,
	*p* < 0.001), R^2^ adj. = 0.442, f^2^ = 0.699	*p* < 0.001), R^2^ adj. = 0.043, f^2^ = 0.045	*p* < 0.001), R^2^ adj. = 0.107, f^2^ = 0.061	*p* < 0.001), R^2^ adj. = 0.228, f^2^ = 0.221
TSC – main effects	β = 0.597 (t = 29.661,	β = −0.216 (t = 3.363,	β = −0.222 (t = 7.149,	β = −0.373 (t = 11.914,
	*p* < 0.001), R^2^ adj. = 0.326	*p* < 0.001), R^2^ adj. = 0.046	*p* < 0.001), R^2^ adj. = 0.048	*p* < 0.001), R^2^ adj. = 0.139
TSC - with controls	β = 0.601 (t = 28.683,	β = −0.154 (t = 4.197,	β = −0.184 (t = 5.862,	β = −0.348 (t = 10.970,
	*p* < 0.001), R^2^ adj. = 0.395, f^2^ = 0.568	*p* < 0.001), R^2^ adj. = 0.063, f^2^ = 0.024	*p* < 0.001), R^2^ adj. = 0.049, f^2^ = 0.036	*p* < 0.001), R^2^ adj. = 0.172, f^2^ = 0.139

As can be seen in Table 6, there is support for most of the expected relationships, with two exceptions. First, while there is a significant influence of gender on the TSC in the expected direction (i.e., men experienced higher levels of digital stress as measured by the TSC), this relationship is not significant for the DSS. It has to be noted though that while this relationship is clearly not significant for the DSS (t = 1.613, *p* = 0.107), it is also not highly significant for the TSC (t = 2.121, *p* = 0.034). Hence, a substantial difference related to the influence of gender on the results should be subject to further investigations in the future. Second, the highest level of education did not have a significant impact on either the DSS or the TSC. As these results are again comparable across measures (as for age), this does not pose a substantial threat to the results in terms of nomological validity.

**FIGURE 5:**
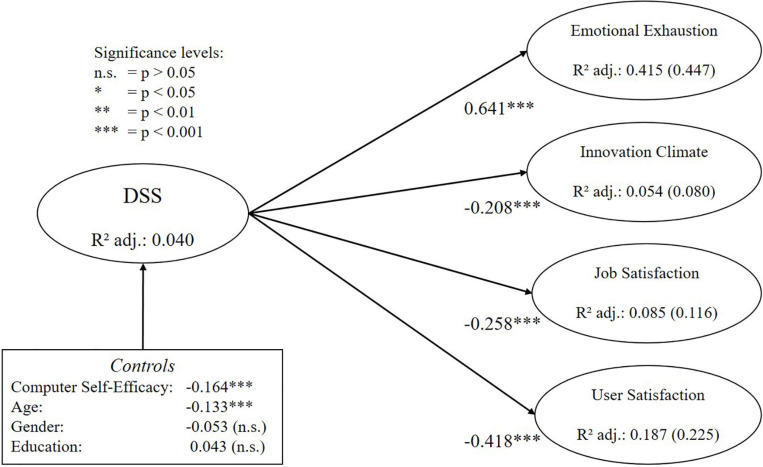
Results of the nomological validity assessment for the DSS.

**FIGURE 6:**
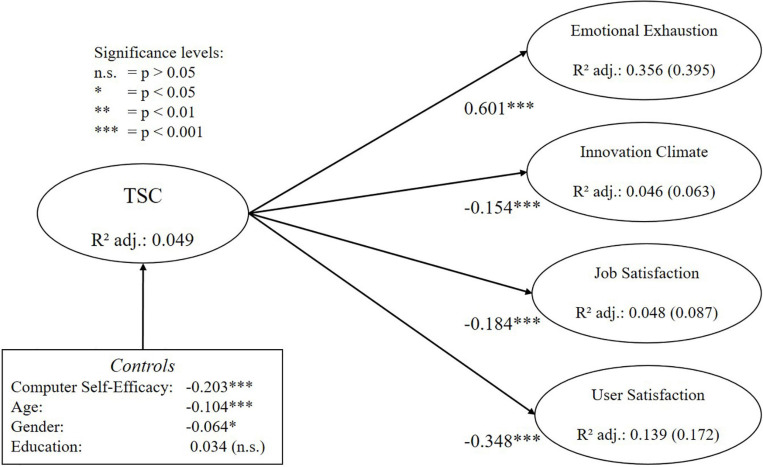
Results of the nomological validity assessment for the TSC.

For both measures (i.e., DSS and TSC), all relationships with criterion variables are significant and remain significant if control variables are included in the structural model (Table 7). Based on values for f^2^, it can also be observed that the new instrument shows a large effect size for emotional exhaustion, a medium effect size for user satisfaction and a small effect size for innovation climate and job satisfaction (small: >0.02, medium: >0.15, large: >0.35, based on Cohen, 1992). In addition, these effect sizes are consistently larger than those of the TSC. Based on the review results of Sarabadani et al. (2018), it can also be assessed whether the found path coefficients are comparable to the findings of other studies or constitute a potential outlier. For user satisfaction, Sarabadani et al. (2018) found that the TSC in previous studies showed path coefficients between -0.17 and -0.42, to which the value of this study with -0.35 is comparable. For job satisfaction, they found that the TSC in previous studies showed path coefficients between -0.13 and -0.41, to which the value of this study with -0.18 is also comparable. Hence, we can assume that the effect sizes found in this study are within an expected range. Finally, the combined included control variables only explain 3.8% of the variance in DSS and 4.9% of the variance in TSC, which further indicates that the found effects are mainly due to the measures for digital stress. Figures 5, 6 below summarize the estimates for the main relationships in the nomological network for the DSS and the TSC respectively. Please note that numbers in brackets for the criterion variables indicate total variance explained by the TSC and the control variables.

## Discussion

The DSS is a state-of-the-art instrument to measure the perception of digital stressors in the workplace. It comprises 50 items in ten stressor categories that can be consolidated in one 2nd order construct to measure digital stress, which has been performed as part of this study. Each indicator is measured with a 7-point Likert scale, with higher values indicating higher levels of stress. It has to be noted though that each stressor category is also a reliable and internally consistent scale in itself and could therefore be applied on its own, although further research is needed to establish the value of these separate scales.

As the DSS is not the first measurement instrument in the area of digital stress, it was tested against the widely used TSC by Ragu-Nathan et al. (2008). As an initial proof of its construct validity, the DSS correlates strongly with the existing measure (r_*s*_ = 0.923, *p* < 0.001), though it provides additional benefits. First, the ten involved stressor categories (1st order constructs) cover aspects that are not included in the existing measure, such as perceptions of distress related to information security or technology unreliability. What follows is that the richness of the phenomenon is better captured by the new instrument and also considers more recent forms of potential stressors. More specifically, the new scale additionally covers stress perceptions caused by data privacy issues (*Invasion*; e.g., a lack of confidentiality of data), the threat of malignant aspects of technology (*Safety*; e.g., malware or malicious e-mails), pressure from the social environment (*Social Environment*; e.g., pressure to respond to e-mails quickly), a lack of usefulness of technology (*Usefulness*; e.g., too many functionalities of ICT with little value to the work of a user), a lack of technical support (*Technical Support*; e.g., help not being available when technical malfunctions occur), and technology that does not behave as expected (*Unreliability*; e.g., long response times or system breakdowns).

Second, it was demonstrated that the DSS can explain more variance for a number of criterion variables, including emotional exhaustion (f^2^ DSS: 0.699, f^2^ TSC: 0.568, Δf^2^ = 0.131), innovation climate (f^2^ DSS: 0.043, f^2^ TSC: 0.024, Δf^2^ = 0.019), job satisfaction (f^2^ DSS: 0.061, f^2^ TSC: 0.036, Δf^2^ = 0.025), and user satisfaction (f^2^ DSS: 0.221, f^2^ TSC: 0.139, Δf^2^ = 0.081). This is further substantiated by a number of hierarchical regressions that were additionally calculated, which show that the DSS can explain variance for each of our four criterion variables over and above the TSC (Δr^2^ for emotional exhaustion of 0.042; Δr^2^ for innovation climate of 0.025; Δr^2^ for job satisfaction of 0.023; and Δr^2^ for user satisfaction of 0.059; see Section 5.3 in the Supplementary Material for further details).

Third, items for the DSS were formulated based on the concept of a discrepancy between situational circumstances and internal standards (e.g., desires) that form distress perceptions. This focus is not given fully in the TSC. Consider, for example, the item “There are constant changes in computer software in our organization” (part of “Techno-Uncertainty” in TSC). Some items in the TSC do *not* conform to the most established conceptualization of stress in psychology, namely the Lazarus model, which defines stress as a discrepancy between a desire and an actual value. Regarding the mentioned sample item, note that the constant changes can be regarded as stressful, but at the same time they could be perceived as beneficial because technologies are less likely to show bugs or other errors due to constant maintenance. Hence TSC is limited in its potential to capture distress.

### Limitations

This study’s limitations are mainly caused by practical constraints inherent in the development of a new measurement instrument as not every step in the development process can be feasibly executed in an ideal fashion (MacKenzie et al., 2011). First, as data were collected through a single cross-sectional survey, the threat of common-method bias must be considered (Podsakoff et al., 2003). As such, several remedies were implemented to reduce the likelihood that the results of this study were affected by this potential issue. This included engagement checks in the survey (i.e., two separate questions that instructed the participant to choose a specific option), splitting the overall samples into sub-samples which then served as an initial means to cross-validate the results (e.g., MacKenzie et al., 2011), and a statistical means to assess the extent of common method bias (i.e., the full-collinearity score, Kock, 2015), which did not indicate any significant bias. Nonetheless though, further investigations should be conducted to cross-validate the results of this study.

In addition, while cross-validation of new measurement instruments is critical, in line with the recommendations by MacKenzie et al. (2011) this study first and foremost ensured that the conceptual definition of the instrument, the development of its indicators, and the specification of the measurement model are sound. Hence, although a large sample of *N* = 1,998 individuals (mostly representative of the US employed population) was the basis for this study, there is further need for cross-validation. In particular, an extension to other countries and languages is needed.

### Directions for Future Research

Through a cross-sectional online survey, this study showed that the perception of distress caused by ICT is positively related to emotional exhaustion and negatively related to satisfaction with ICT at work. This investigation showed that a linear relationship between the DSS and these constructs already explains a substantial share of their variance (i.e., R^2^ adj. without controls for emotional exhaustion of 0.409; R^2^ adj. without controls for user satisfaction of 0.192). In the wake of further investigations into the types of relationships that digital stressors show with outcome variables, it should be kept in mind that the relevance of stressors included in the DSS may change. In fact, the changing nature of our technological environment was one of the main motivations for the development of the DSS and the investigation of its dimensionality (i.e., stressor categories). Regular updates of instruments such as the DSS are crucial to ensure that stressors that appear more relevant over time in practice (i.e., the work context) are not overlooked in research and organizational practice. Likewise, it has to be acknowledged that stressors may become obsolete or are found to be less relevant than others over time and therefore have to be removed from the set of stressor categories included in the DSS. This is particularly true when the goal is to investigate digital stress in the context of more specific participant groups (e.g., less formally educated people, Marchiori et al., 2018) or technologies (e.g., social media, Maier et al., 2015). Hence, studies that intend to apply the DSS should always reflect on the composition of its stressors and argue their relevance for the specific research question.

While this study investigated the role of digital stressors within a nomological network of important outcome variables that have previously been found to be related to digital stress (i.e., emotional exhaustion, innovation climate, job satisfaction, user satisfaction), as well as a set of control variables that have been found to influence digital stress appraisal (i.e., age, gender, education, computer self-efficacy), further variables should be added to this nomological network in future studies. This will further bolster the validity of the proposed instrument (e.g., individual characteristics such as personality characteristics like negative affectivity and extraversion, Ayyagari et al., 2011; or organizational characteristics such as social norms related to technology use, Barley et al., 2011). In addition, relationships between these new variables which are conceptualized in seminal theories (e.g., in the organizational stress domain the Person-Environment Fit Theory, Edwards et al., 1998; or in the technology use domain the Technology Acceptance Model, Davis, 1989) should also be considered as a model extension in future research.

While this study highlighted the convergent validity of the DSS with another measure related to digital stress (i.e., the TSC), it should also be a focus of future research to establish further convergent validity and/or discriminant validity with other potentially related measures, particularly in the area of occupational stress. Potential scales that could be the subject of such investigations have been proposed in the past such as measures of stress perceptions at work (e.g., Hackman and Oldham, 1975; Motowidlo et al., 1986; Williams and Cooper, 1998; Siegrist et al., 2009).

Overall, our newly developed instrument is a complement and update to the already existing set of measures in the field of occupational stress research, and particularly studies into digital stress. It is hoped that the instrument’s usefulness, which has been demonstrated in this study, will be further validated and extended through application in future research.

## Data Availability Statement

The raw data supporting the conclusion of this manuscript will be made available by the authors, without undue reservation.

## Author Contributions

TF, MR, and RR conceptualized the development approach. TF collected the data. TF and MR processed the data and performed the analyses. TF drafted the manuscript. All authors discussed the results and commented on the manuscript.

## Conflict of Interest

The authors declare that the research was conducted in the absence of any commercial or financial relationships that could be construed as a potential conflict of interest.

## Footnotes

1 https://www.respondi.com/
