# Supplementary material for: The Digital Stressors Scale: Development and Validation of a New Survey Instrument to Measure Digital Stress Perceptions in the Workplace Context
Source: Front Psychol. 2021 Mar 12;12:607598. doi: 10.3389/fpsyg.2021.607598 (PMC7994533; doi:10.3389/fpsyg.2021.607598)
Supplement: Supplementary file 1 [file Data_Sheet_1.docx]

Supplementary Material

This document provides further information on the development process of the Digital Stressors Scale. Should you require further information, please do not hesitate to contact the authors of the article.

# Dimensionality of Digital Stress

Previous conceptualizations of work stress (also referred to as job stress, occupational stress, or organizational stress) were reviewed to create a list of stressors (i.e., Bosma, Peter, Siegrist, & Marmot, 1998; Cooper & Cartwright, 1994; Cooper & Payne, 1978; Ivancevich & Matteson, 1980; Karasek et al., 1998; Marshall & Cooper, 1979; Parker & DeCotiis, 1983; Peters & O'Connor, 1980; Williams & Cooper, 1998; see also Kahn & Byosiere, 1992 for a meta-review of conceptualizations of occupational stress). Initial categories of stressors found on the individual level (e.g., job characteristics) and the organizational level (e.g., decision-making processes) are summarized in Table 1.

**Table 1.** Stressors commonly Found in Organizational Stress Research

|  | Cooper and Payne (1978) | Marshall and Cooper (1979) | Ivancevich and Matteson (1980) | Peters and O'Connor (1980) | Parker and DeCotiis (1983) | Kahn and Byosiere (1992) | Cooper and Cartwright (1994) | Bosma et al. (1998) | Karasek et al. (1998) | Williams and Cooper (1998) |
| --- | --- | --- | --- | --- | --- | --- | --- | --- | --- | --- |
| *Boredom* (e.g. underutilization, level of task variety) | X |  |  |  | X |  |  |  |  |  |
| *Complexity* (e.g., task preparation and characteristics of equipment, task complexity) |  |  |  | X |  | X |  |  |  |  |
| *Conflicts* (e.g., home-work interferences) |  |  |  |  |  |  | X |  |  | X |
| *Control* (e.g., job control, autonomy, decision latitude) | X |  |  |  |  |  |  | X | X |  |
| *Costs* (e.g., costs, demands, and responsibilities) | X | X | X |  | X |  |  |  |  | X |
| *Insecurity* (e.g., job insecurity and career ambiguity) | X | X | X |  |  | X | X | X | X |  |
| *Involvement* (e.g., lack of participation in decision-making processes) |  | X |  |  |  | X |  |  |  |  |
| *Overload* (e.g., workload and work-related effort) | X | X | X |  | X | X |  | X | X | X |
| *Role Stress* (e.g., role conflict and role ambiguity) | X | X | X |  | X | X | X |  |  | X |
| *Social Environment* (e.g., demands of the social environment and norms) |  |  |  |  | X | X | X |  |  | X |
| *Technical Support* (e.g., support services) | X | X |  | X |  | X |  |  |  |  |

It is important to note that the focus here is on stressors that are commonly found in occupational stress research. Stressors that are specific to a certain theory (e.g., lack of reward, Bosma et al., 1998) or represent a specific aspect of one of the listed stressors (e.g., bullying as a stressful aspect of the social environment) are not included. Yet, such stressors should not be excluded categorically, but rather considered when more specific scales are developed (e.g., focusing on the potential for social conflict caused by ICT).

Based on these findings from a review of previous conceptualizations of organizational stress, the literature on digital stress was further reviewed for an indication on how to transfer these dimensions to the more specific domain of digital stress. Further, stressors that are specific to digital stress (i.e., Privacy invasion, Safety, Unreliability, and Usefulness) were added. Importantly, in the resulting preliminary factor structure, not only potential stressors that are directly related to technology were included, but also such stressors that may arise from the technological environment. Consider, for example, technology that behaves in an unreliable way (e.g., software breakdowns or long response times). Unreliable behavior in itself can be a substantial stressor (e.g., Riedl, Kindermann, Auinger, & Javor, 2012), but with sufficient coping resources (e.g., technical support) its negative effects can be mitigated. Yet, if such resources are not available (e.g., no IT helpdesk in small companies), the perceived possibility of unreliable ICT behavior alone can already be considered a substantial stressor. These circumstances are therefore also included as potential stressors in a preliminary list of digital stress categories.

# Initial Item Pool

In Table 2, all 138 items generated for the 15 stressor categories are listed.

**Table 2.** Initial Pool of 138 Items for the DSS

| 1. **Boredom** 2. Due to ICT I have too little to do. 3. Due to ICT my work is too monotonous. 4. Due to ICT I always have to work in accordance with the same tedious routines. 5. ICT automatically rule out too many decision alternatives that I would be willing to try. 6. ICT take away too many of the aspects of my work that I find entertaining. 7. ICT have automatized work tasks that I really enjoyed doing myself. 8. Sometimes I feel bored, because ICT made my job too easy and less thrilling. 9. ICT have made my job less interesting. 10. ICT undertake tasks for me and hence my job is monotonous. |
| --- |
| 1. **Complexity** 2. I often find it too complicated to accomplish a task using the ICT that are available to me at work. 3. I often need more time than expected to accomplish a task using the ICT that are available to me at work. 4. I feel that the ICT that are available to me at work are too confusing. 5. Often I find the ICT that are available to me at work not user-friendly enough. 6. I often do not find enough time to keep up with new functionalities of ICT at work. 7. There are too many functionalities of the ICT at work that I only require rarely and I need to learn how to use every time again. 8. It would take me too long to completely figure out how to use the ICT that are available to me at work. 9. I feel that using ICT is often a complex process. 10. The complexity involved in using ICT is typically high. |
| 1. **Conflicts** 2. ICT enables private problems to reach me too often at work. 3. ICT enables work-related problems to reach me too often at home. 4. I feel that my work routine suffers due to ICT enabling private problems to reach me everywhere. 5. I feel that my private life suffers due to ICT enabling work-related problems to reach me everywhere. 6. It is too hard for me to keep my private life and work life separated due to ICT. 7. ICT make it harder to create clear boundaries between my private life and work life. 8. My work-life balance suffers due to ICT. 9. Distinguishing the use of ICT for work and for private life is difficult. 10. The ubiquity of ICT disturbs my work-life balance. |
| 1. **Control** 2. I think it is bad when ICT dictate how I should do my work (e.g., when work routines are highly penetrated by ICT). 3. I think that I am too dependent on ICT at work. 4. Due to ICT I do not have the autonomy at work that I would like to have. 5. I think that I can work more autonomously due to ICT (e.g., when I can accomplish the same task with different ICT). **(-)** 6. I think that ICT have too much impact on the timing of my breaks (i.e., when I can take a break and when not). 7. ICT force me to follow a standardized workflow; I cannot work according to my desired work schedule. 8. ICT give me more flexibility at work; it is easier to reschedule tasks if needed. **(-)** 9. I cannot experiment with new ways of doing my work, as ICT force me into specific work routines. 10. ICT define the way in which I have to do my work. |
| 1. **Costs** 2. I have to invest more time into the adaptation of ICT to my individual needs than I would like to. 3. I think that the time needed to adapt ICT to my individual needs is worth it. **(-)** 4. I feel uncomfortable when replacing an existing ICT with a new one as I have often already invested a lot of time into their adaptation to my own needs. 5. I often fear using new ICT, because of the cost that would be involved should I not do so. 6. I often feel uncomfortable when I ask for old ICT to be replaced by new ICT as I know that new ICT cost a lot of money. 7. I think that no matter the amount of money invested into ICT, it is always worth it. **(-)** 8. I often feel that setting up new ICT is too much of a hassle. 9. I am often annoyed by ICT updates as they are not worth my time. 10. I think that the time needed to adapt ICT to my individual needs is not worth it. |
| 1. **Insecurity** 2. I feel that my job position is threatened due to ICT. 3. I feel that it is threatening that my job could be accomplished in an automated fashion due to ICT. 4. I fear that I could be replaced by individuals with better technology expertise at work. 5. I fear that I could be replaced at work due to the increasing standardization of work processes, which is enabled by ICT. 6. I fear that what I like about my job will one day be obsolete due to ICT. 7. I cannot be optimistic about my long-term job security because of the threat of ICT automatization. 8. It is too hard for me to get involved in my job routine, as I feel that it will change quickly anyway due to ICT. 9. I fear that I could be replaced by machines. 10. I fear that digitalization will cost me my job. |
| 1. **Involvement** 2. If decisions are made in my work environment to use new ICT or replace existing ICT than I am getting too much involved in the decision process. 3. If decisions are made in my work environment to use new ICT or replace existing ICT than I am getting sufficiently involved in the decision process. **(-)** 4. It is frustrating if new ICT is introduced at the workplace without being asked what we think about it beforehand. 5. I am informed too often about the new ICT I have to use at work when there is no time left to do anything about it. 6. I do not get involved enough when the requirements concerning new ICT are assessed. 7. I feel that new ICT is too often forced upon me at work without an opportunity to express my opinion about it. 8. I am annoyed when my demands for new or changed ICT are ignored at work. 9. I am rarely involved in organization decision processes about hardware and software acquisitions. 10. Management involves me in decisions about hardware and software purchases. **(-)** |
| 1. **Overload** 2. Due to ICT I have too much to do. 3. Due to ICT I have a too large variety of different things to do at work. 4. ICT provides me with more information than I can handle. 5. ICT make it too easy for other individuals to send me additional work. 6. I never have any spare time, because my schedule is too tightly organized by ICT. 7. ICT make it too easy for other individuals to request my opinion or assistance for all sorts of tasks. 8. There is a constant surge of work-related information coming in through ICT that I just cannot keep up with. 9. ICT make it easier for colleagues to delegate work to me. 10. ICT is the reason why I have too much work. |
| 1. **Privacy Invasion** 2. I fear that my use of ICT is less confidential than I would like to. 3. I fear that I can be more easily monitored due to ICT than I would like to. 4. I fear that I can be more easily monitored by my superiors due to ICT than I would like to. 5. I fear that the information that I exchange using ICT is not as protected as I would like to. 6. I fear that malevolent outsiders (e.g., hackers) can easily copy my identity due to ICT. 7. My personal information is too easily accessible due to ICT. 8. It is unnerving that I can never be sure whether my activity with ICT can be tracked or not. 9. I fear that my personal data can easily be stolen by others online. 10. I fear that my activities on the Internet can be tracked by others. |
| 1. **Role stress** 2. It is too difficult for me to concentrate on my work as I could be disrupted by ICT that I might need for other tasks at any point. 3. It is too difficult for me to concentrate on my work as I am getting constantly disrupted by ICT that I do not actually need to accomplish my tasks. 4. It is too difficult for me to concentrate on my work and cope with ICT-related problems at the same time. 5. Problems with ICT (e.g., technical malfunctions) require more of my attention than I would like to give them. 6. I am too often interrupted by electronic messages (e.g., emails) during work. 7. I think that it is bad, if I am interrupted by electronic media (e.g., due to pop-ups on websites) during work. 8. Due to frequent technical interruptions (e.g., reminders by other systems than the one that I currently work with) I can work in a less orderly fashion than I would like to. 9. I cannot concentrate on my work as much as I want to due to the constant distraction afforded by electronic media (e.g., Facebook). 10. Security measures (e.g., warning messages) interrupt me too often during my work tasks. 11. My colleagues feel encouraged to interrupt me too often during work when I am online in electronic media (e.g., chat tools). 12. The constant multitasking that is needed to handle all the ICT I need for work does not allow me to concentrate on the task at hand sufficiently. 13. ICT make it too easy for new tasks and demands to reach me before I have the chance to finish what I am doing at the moment. |
| 1. **Safety** 2. I have to worry too often, whether I might download malicious programs. 3. I have to worry too often, whether I might receive malicious e-mails. 4. I have to cope too often with rigorous security measures that are implemented using ICT (e.g., resulting in e-mails being wrongly classified as junk e-mail). 5. I have to worry too often, whether I might damage ICT when interacting with them. 6. Too much time is needed to sort out spam mails during work. 7. I fear that hackers might get access to company secrets through a mistake of mine. 8. I feel anxious when I get an e-mail from somebody that I do not know as it could be a malevolent attack. 9. E-Mails whose sender I do not know make me nervous. 10. A considerable number of e-mails are sent with evil intent. |
| 1. **Social environment** 2. Due to ICT I have too much to do with the problems of others. 3. Due to ICT I have less contact with other people than I would like to. 4. I am too often prompted by people in my close environment to use specific ICT. 5. I think that ICT generate too much of an expectation that I have to be reachable everywhere and at any time. 6. Too much time gets lost at work because of irrelevant communication with other people on social media. 7. I feel that ICT create unwanted social norms (e.g., the expectation that e-mails should be answered right away). 8. It is too hard to take a break from social interactions at work due to the communication possibilities of ICT. 9. ICT helps me to avoid interaction with others. **(-)** 10. Due to ICT I have more contact with beloved individuals. **(-)** |
| 1. **Technical support** 2. I have to worry about ICT-related problems as our organization does not offer enough support for their removal. 3. In the case of ICT-related problems, it happens too often that there is not enough support available at work. 4. I think that it happens too often that technical support is not available when I need it. 5. I often have to wait for a long time because technical problems cannot be adequately solved in our organization. 6. It annoys me if it takes longer than expected for a technical problem to be solved at work. 7. Whenever I encounter a technical problem at work, I blame our incompetent technical support for it. 8. I fear that a technical problem I have at work could not be solved by anyone else at work. 9. The help desk in my company works perfectly. **(-)** 10. The IT service desk members in my company always fulfill my expectations. **(-)** |
| 1. **Unreliability** 2. I think that I am too often confronted with unexpected behavior of the ICT I use at work (e.g., breakdowns or long response times). 3. I think that I lose too much time due to technical malfunctions. 4. I think that I spend too much time trying to fix technical malfunctions. 5. There is just too much of my time at work wasted coping with the unreliability of ICT. 6. I fear that a system might crash if I need it most. 7. I fear that a lot of work might get lost if do not save my progress often enough. 8. The daily hassles with ICT (e.g., slow programs or unexpected behavior) are really bothering me. 9. The unreliability of ICT is its most dominant characteristic. 10. I fear that a system breaks down when I need it most. |
| 1. **Usefulness** 2. I think that the demands of my work and the functions provided by the ICT I use do not fit sufficiently. 3. I think that I do not gain enough benefits from using the ICT that I am provided with at work for my tasks. 4. At work, I profit from the synergies between my tasks and the functions that are provided by the ICT that I can use. **(-)** 5. The ICT I use at work are full of too many functionalities that I never need. 6. It requires too many different systems to fulfill the tasks that I have to do during an average day at work. 7. I think that most of the ICT I am supplied with at work is not useful enough and I could work without it. 8. I have to constantly switch between systems to fulfill a task, which can get annoying. 9. The ICT at work is ideal, because one single system offers all functionalities that I need to accomplish my work. **(-)** 10. The task-technology fit of the ICT that I use at work is excellent. **(-)** |
| **(-)** indicates reverse-scored items. |

# Card Sorting

In this section, additional details on the open card sorting and closed card sorting rounds are provided. **Open Sorting.** Five individuals participated in the *open card sorting task* (3f, 2m; average age of 28 years; two graduate students, two PhD students, one office worker). All individuals were placed in the same room and provided with the necessary equipment to conduct the online card sorting. Due to the large amount of 138 items, an online tool was used instead of printed cards (i.e., https://www.provenbyusers.com; please refer to Figure 1 for a screenshot of the tool). The duration of the exercise was about four hours, which included an introduction to the goal of the exercise, an explanation of digital stress and ICT, a training exercise with 20 items, the individual sorting of all 138 items, a phase to individually find definitions for the formed groups and a final group discussion of the stressor categories that were used by more than one participant. Each participant received a € 20 Amazon gift certificate for taking part in the task.

The main goal of this task was to freely form groups of stress categories. The five participants created between 12 and 16 groups for the 138 statements, which is already reasonably close to the initial 15 stressor categories. Most stressor categories were replicated by the participants, though also some additional, more specific groups were formed. In particular, five potential stressor categories emerged that were either sub-dimensions of existing stressor categories or new combinations of aspects from different stressor categories namely *Distraction through ICT* (mostly Role Stress items), *Isolation by ICT* (mostly Social Environment items), *Multitasking* (mostly Usefulness and Overload items), *Lack of Training* (mostly Complexity items) and *Uncertainty* (mostly Complexity and Costs items). Together with the existing 15 stressor categories, definitions for these new categories were formulated in the group and then used as input for the closed sorting exercise (see Table 3 below). In addition, the results of the open sorting exercise allowed for an initial check of internal consistency and discriminant validity for the formed groups (i.e., how often certain items were grouped together; see Table 4 below), which indicated potential for some stressor categories to be combined, while most of them already performed well (i.e., items were often group together as originally suggested).

**Closed Sorting**. The card sorting exercise was then repeated with an additional five participants (4f, 1m; average age of 24 years; two undergraduate students, one PhD student, two professionals). All individuals conducted the exercise online using the same tool as before and € 20 Amazon gift certificates were raffled amongst them. The task was a *closed sorting*, which included the same 138 items as before, but this time the participants had to sort the items into the previously created groups (see Table 3 for the group descriptions and Table 4 for the result of the two card sorting rounds). Two additional groups were provided to indicate the quality of the items ("Not clear") and the potential for missing categories ("Does not fit into ay group"). Participants also were allowed to duplicate items and sort them into several groups. Figure 1 provides a screenshot of the online tool used for the card sorting exercise.


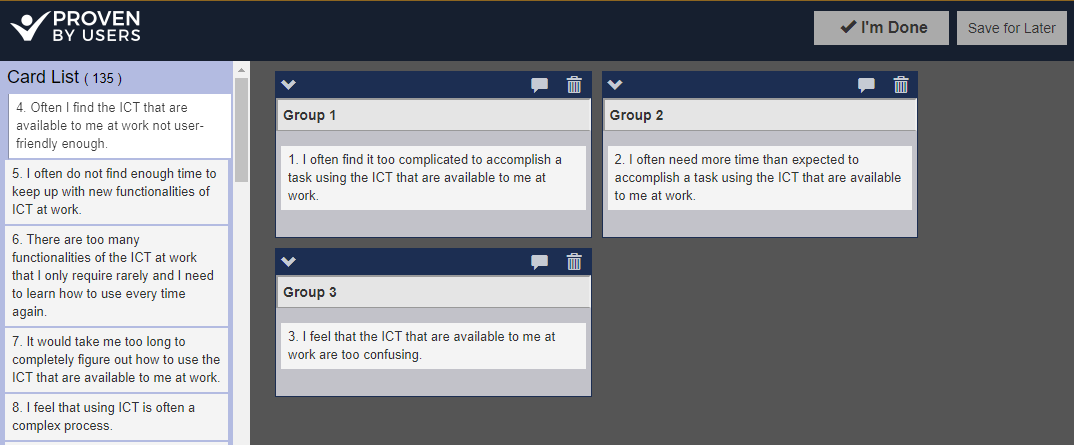


**Figure 1.** Screenshot of the Card Sorting Tool by "ProvenbyUsers"

**Table 3.** Item Group Definitions Created during the Open Sorting Round

| *Group* | *Description* |
| --- | --- |
| Boredom | Distress caused by a lack of entertainment and creativity at work, caused by too monotonous work schedules implemented through ICT. |
| Complexity | Distress caused by highly complex ICT, with too many, confusing functionalities. |
| Conflicts | Distress caused by conflicts between work life and private life, with ICT erasing the boundaries between these domains. |
| Costs | Distress caused by the costs that are linked to ICT such as their monetary cost, as well as cognitive effort and time needed to adapt and understand ICT. |
| Insecurity | Stress caused by the increasing importance of ICT and diminished importance of own job skills with the potential of job loss. |
| Lack of Control | Distress caused by the lack of control over one's job routines, caused by an increasing dependence on ICT and workflows implemented through ICT. |
| Lack of Involvement | Distress caused by a lack of involvement in decision-making related to ICT, such as the collection of requirements for new ICT or evaluation of existing ICT. |
| Lack of Technical Support | Distress caused by a lack of technical support in the case of ICT-related problems, such as a missing or incompetent helpdesk. |
| Overload | Distress caused by too much work or information being transmitted through ICT. |
| Privacy Invasion | Distress caused by a lack of privacy of data that is processed at work; fear of being monitored by one's employer or other individuals at work. |
| Role Stress | Distress caused by differing demands that have to be fulfilled at work; ICT requires the individual to assume many different roles aside from being an employee such as a technician when ICT problems arise or a security expert when security-related warnings pop up. |
| Safety Threats | Distress caused by potential damages and threats to ICT safety that can arise when interacting with ICT such as computer viruses or malevolent outsiders (e.g., hackers). |
| Social Demands | Distress caused by social demands that can be more easily implemented through ICT such as constant messaging and the pressure to respond immediately. |
| Unreliability | Distress caused by unreliable behavior of ICT such as long response times and malfunctions (e.g., system breakdowns). |
| Lack of Usefulness | Distress caused by a lack of usefulness of provided ICT for work tasks; too many systems, each providing only a small selection of useful functionality, but also systems with a large selection of functionalities of which only few are frequently used for work purposes. |
| *Potential new categories:* |  |
| Distraction (through ICT) | Distress caused by the constant distractions that are caused by ICT such as pop-up messages by messaging services or automatically generated security warnings. |
| Isolation (by ICT) | Distress caused by communication norms and routines that reduce one's preferred form of communication (e.g., physical conversations) with other individuals. |
| Multitasking | Distress caused by the constant need to switch between different systems and the demands caused by ICT (e.g., doing work and coping with ICT-related problems). |
| Lack of Training | Distress caused by the perceived lack of training related to the ICT provided at work; users feel that they do not possess the necessary skills and qualifications needed to properly work with ICT currently and in the future. |
| Uncertainty | Distress caused by constant changes in the technological environment and the demands that these changes create (e.g., learning about new systems and personalizing new or changed systems). |

Table 4 includes the results of an initial check for internal consistency and discriminant validity based on the open sorting exercise. For each stressor category, it is shown to which degree, on average, items were sorted into the same category. Diagonally (in italics), the number indicates the percentage to which items who are supposedly in the same stressor category were also sorted together. For example, in the case of *Boredom*, items that were formulated for this category were also sorted together in the same group on average in 81% of the cases. In addition, the numbers below the diagonal percentages indicate to which extent items from one stressor category were on average grouped together with items from a different stressor category. For example, in the case of *Boredom* and *Complexity*, items were sorted together on average only in 1% of the cases. Items being sorted together into their supposed group gives an initial indication of the internal consistency of the stressor category (i.e., a high number on the diagonal line), while items being sorted together with items from other groups gives an initial indication of the discriminant validity of a stressor category (i.e., low numbers below the diagonal line). To summarize the potential discriminant validity for each stressor category, two additional values are included in separate lines on the bottom of the table. Maximum closeness indicates the highest level of similarity to another stressor category (e.g., for *Boredom*, on average 29% of items were grouped together with items from the stressor category *Control*), while the minimum difference value indicates the lowest level of distance to any other stressor category (i.e., the level of internal similarity, such as 81% for Boredom, minus the highest level of similarity with another stressor category, such as 29% for Boredom). Based on these results, some stressor categories can be initially flagged as problematic, such as *Control* and *Costs*, with potentially low levels of internal consistency and low levels of discriminant validity. Other stressor categories already perform well though, such as *Conflicts* and *Privacy Invasion*.

**Table 4**. Similarity Matrix for Stressor Categories based on Open Sorting and Closed Sorting

|  | Boredom | Complexity | Conflicts | Control | Costs | Insecurity | Involvement | Overload | Privacy Invasion | Role Stress | Safety | Social Environment | Tech. Support | Unreliability | Usefulness |
| --- | --- | --- | --- | --- | --- | --- | --- | --- | --- | --- | --- | --- | --- | --- | --- |
| Boredom | 81 (45) |  |  |  |  |  |  |  |  |  |  |  |  |  |  |
| Complexity | 1 (2) | 50 (38) |  |  |  |  |  |  |  |  |  |  |  |  |  |
| Conflicts | 0 (0) | 0 (0) | 100 (81) |  |  |  |  |  |  |  |  |  |  |  |  |
| Control | 29 (24) | 5 (3) | 0 (0) | 53 (78) |  |  |  |  |  |  |  |  |  |  |  |
| Costs | 0 (1) | 17 (16) | 0 (2) | 3 (1) | 43 (22) |  |  |  |  |  |  |  |  |  |  |
| Insecurity | 21 (12) | 7 (1) | 0 (0) | 8 (4) | 3 (1) | 79 (78) |  |  |  |  |  |  |  |  |  |
| Involvement | 0 (2) | 7 (1) | 0 (0) | 5 (6) | 14 (1) | 1 (1) | 79 (63) |  |  |  |  |  |  |  |  |
| Overload | 8 (5) | 9 (5) | 0 (4) | 14 (16) | 4 (5) | 2 (1) | 1 (3) | 66 (46) |  |  |  |  |  |  |  |
| Privacy I | 0 (0) | 1 (0) | 0 (0) | 2 (0) | 1 (0) | 0 (5) | 1 (0) | 0 (0) | 91 (71) |  |  |  |  |  |  |
| Role Stress | 1 (2) | 1 (4) | 0 (0) | 2 (5) | 6 (7) | 0 (0) | 1 (4) | 11 (6) | 0 (0) | 64 (43) |  |  |  |  |  |
| Safety | 0 (1) | 3 (1) | 0 (0) | 1 (0) | 1 (2) | 0 (0) | 0 (1) | 2 (1) | 23 (9) | 8 (7) | 53 (56) |  |  |  |  |
| Social Environment | 0 (4) | 1 (1) | 9 (17) | 0 (10) | 1 (3) | 0 (1) | 2 (2) | 5 (18) | 0 (0) | 7 (20) | 1 (4) | 60 (38) |  |  |  |
| Tech. Support | 0 (0) | 0 (1) | 0 (0) | 2 (0) | 0 (1) | 0 (0) | 2 (0) | 0 (1) | 0 (0) | 5 (5) | 2 (0) | 0 (1) | 96 (67) |  |  |
| Unreliability | 0 (2) | 2 (2) | 0 (0) | 3 (2) | 2 (3) | 0 (4) | 3 (2) | 4 (0) | 0 (0) | 9 (1) | 5 (2) | 1 (0) | 52 (15) | 74 (68) |  |
| Usefulness | 1 (2) | 18 (23) | 0 (0) | 2 (3) | 5 (6) | 0 (0) | 1 (4) | 5 (0) | 0 (0) | 5 (4) | 1 (1) | 0 (0) | 6 (0) | 6 (0) | 46 (40) |
| Max. Closeness | 29 (7) | 18 (23) | 9 (17) | 29 (24) | 14 (7) | 21 (12) | 14 (16) | 29 (24) | 23 (9) | 23 (20) | 9 (20) | 18 (23) | 52 (15) | 52 (15) | 6 (5) |
| Min. Difference | 59 (38) | 32 (15) | 91 (65) | 23 (54) | 29 (15) | 58 (67) | 65 (47) | 37 (22) | 68 (61) | 40 (23) | 44 (36) | 42 (16) | 44 (52) | 23 (53) | 39 (35) |
| Values outside of brackets represent results from the open sorting round, while values in brackets represent results from the closed sorting round. | | | | | | | | | | | | | | | |

# Measurement Model Evaluation

In this section, reliability and validity statistics for the DSS and TSC as well as criterion variables and control variables are reported. Calculations in this section are based on sub-sample one.

## Results of Exploratory Factor Analysis (EFA)

**Table 5.** Initial EFA of all 138 items

|  | 1 | 2 | 3 | 4 | 5 | 6 | 7 | 8 | 9 | 10 | 11 | 12 | 13 | 14 | 15 | 16 | 17 |
| --- | --- | --- | --- | --- | --- | --- | --- | --- | --- | --- | --- | --- | --- | --- | --- | --- | --- |
| DSS001 | 0.932 | 0.042 | -0.027 | -0.077 | -0.013 | 0.035 | 0.030 | 0.042 | -0.058 | -0.027 | -0.169 | 0.067 | -0.005 | 0.028 | 0.084 | 0.048 | -0.068 |
| DSS002 | 0.836 | -0.034 | 0.008 | -0.031 | 0.027 | -0.017 | -0.034 | -0.046 | 0.017 | -0.058 | 0.013 | 0.097 | -0.003 | 0.017 | 0.064 | -0.067 | 0.011 |
| DSS003 | 1.000 | -0.068 | 0.000 | -0.039 | 0.010 | -0.013 | 0.049 | -0.081 | -0.048 | 0.002 | -0.177 | 0.119 | 0.028 | -0.032 | 0.076 | 0.039 | 0.040 |
| DSS004 | 0.958 | -0.060 | -0.038 | 0.012 | 0.008 | 0.046 | 0.017 | -0.092 | 0.034 | -0.171 | -0.031 | 0.087 | 0.015 | -0.016 | -0.004 | 0.106 | 0.095 |
| DSS005 | 0.758 | -0.049 | 0.021 | 0.043 | 0.003 | -0.056 | -0.041 | 0.014 | 0.030 | -0.011 | -0.046 | 0.270 | 0.054 | -0.019 | 0.033 | -0.086 | 0.063 |
| DSS006 | 0.632 | -0.051 | 0.062 | -0.101 | -0.021 | 0.051 | -0.095 | 0.097 | 0.013 | 0.031 | 0.146 | 0.356 | -0.029 | -0.036 | 0.062 | 0.066 | -0.048 |
| DSS007 | 0.900 | -0.153 | 0.016 | -0.058 | -0.020 | 0.038 | 0.033 | -0.029 | -0.005 | 0.034 | -0.159 | 0.343 | 0.034 | -0.038 | 0.061 | -0.026 | 0.023 |
| DSS008 | 0.706 | -0.016 | 0.042 | 0.065 | -0.034 | 0.055 | -0.017 | 0.083 | 0.004 | -0.159 | -0.145 | 0.214 | 0.121 | -0.033 | -0.050 | 0.005 | -0.057 |
| DSS009 | 0.792 | -0.047 | 0.054 | 0.121 | 0.042 | -0.011 | -0.020 | 0.009 | -0.026 | -0.244 | -0.034 | 0.183 | 0.155 | -0.114 | -0.116 | 0.088 | -0.143 |
| DSS010 | 0.130 | 0.514 | 0.012 | -0.018 | 0.009 | -0.017 | -0.031 | 0.063 | -0.016 | 0.102 | 0.096 | 0.027 | -0.046 | 0.021 | 0.134 | 0.016 | -0.001 |
| DSS011 | -0.060 | 0.806 | 0.028 | -0.008 | 0.009 | 0.033 | -0.025 | 0.037 | -0.066 | -0.044 | 0.062 | -0.089 | 0.012 | -0.090 | 0.042 | 0.071 | -0.019 |
| DSS012 | 0.335 | 0.466 | -0.055 | 0.041 | -0.014 | -0.063 | 0.059 | 0.072 | -0.024 | 0.061 | 0.023 | -0.030 | 0.000 | 0.057 | 0.133 | 0.009 | -0.092 |
| DSS013 | -0.070 | 0.869 | 0.048 | -0.023 | -0.013 | 0.104 | 0.023 | -0.069 | 0.010 | -0.094 | 0.067 | -0.056 | -0.011 | -0.035 | 0.020 | 0.036 | 0.048 |
| DSS014 | -0.010 | 0.931 | 0.016 | -0.130 | -0.040 | 0.037 | -0.026 | 0.052 | 0.000 | -0.099 | -0.009 | 0.018 | -0.007 | -0.015 | 0.047 | -0.022 | 0.012 |
| DSS015 | -0.014 | 0.839 | 0.013 | 0.079 | -0.032 | -0.046 | -0.058 | 0.029 | -0.032 | -0.094 | 0.001 | 0.015 | -0.013 | -0.016 | 0.078 | 0.054 | 0.084 |
| DSS016 | 0.132 | 0.802 | 0.050 | -0.010 | -0.008 | 0.001 | 0.047 | -0.078 | 0.048 | -0.076 | -0.065 | -0.054 | 0.065 | -0.069 | -0.003 | -0.045 | 0.036 |
| DSS017 | 0.082 | 0.694 | -0.035 | -0.022 | 0.050 | 0.100 | -0.071 | -0.013 | 0.010 | 0.027 | -0.200 | -0.003 | 0.019 | 0.106 | 0.134 | 0.014 | 0.022 |
| DSS018 | 0.292 | 0.633 | -0.005 | 0.067 | 0.004 | -0.064 | 0.041 | -0.057 | -0.017 | -0.085 | -0.051 | -0.071 | 0.024 | -0.028 | 0.004 | -0.104 | 0.073 |
| DSS019 | 0.381 | 0.036 | -0.010 | 0.185 | -0.055 | 0.053 | 0.020 | -0.032 | -0.009 | 0.042 | 0.088 | 0.150 | -0.023 | 0.050 | -0.133 | 0.181 | -0.016 |
| DSS020 | 0.234 | 0.087 | 0.091 | 0.055 | 0.149 | -0.073 | 0.025 | 0.011 | 0.042 | 0.155 | 0.123 | 0.071 | 0.184 | -0.071 | 0.103 | -0.143 | 0.087 |
| DSS021 | 0.485 | -0.002 | 0.161 | 0.053 | -0.144 | -0.026 | 0.004 | -0.011 | -0.044 | 0.094 | 0.125 | -0.026 | 0.078 | 0.064 | -0.031 | 0.013 | -0.031 |
| DSS022 | -0.040 | 0.019 | 0.070 | -0.028 | -0.573 | 0.036 | 0.008 | 0.095 | -0.006 | -0.123 | -0.052 | -0.001 | -0.136 | 0.027 | 0.002 | 0.070 | -0.107 |
| DSS023 | 0.374 | 0.273 | 0.070 | -0.028 | -0.017 | 0.031 | 0.014 | 0.097 | -0.074 | 0.014 | -0.049 | -0.141 | 0.045 | 0.056 | -0.077 | -0.043 | -0.076 |
| DSS024 | 0.474 | 0.035 | 0.041 | -0.047 | -0.092 | 0.099 | -0.020 | -0.014 | -0.049 | 0.107 | 0.128 | -0.083 | 0.087 | 0.056 | -0.128 | -0.023 | -0.016 |
| DSS025 | 0.177 | -0.045 | -0.006 | 0.043 | -0.631 | 0.043 | 0.044 | -0.034 | 0.019 | -0.015 | -0.044 | -0.006 | -0.072 | -0.027 | -0.087 | 0.022 | 0.139 |
| DSS026 | 0.487 | -0.043 | 0.018 | -0.041 | -0.100 | 0.072 | 0.053 | 0.114 | -0.064 | 0.178 | 0.036 | 0.014 | 0.218 | 0.030 | -0.007 | 0.036 | 0.096 |
| DSS027 | 0.191 | 0.058 | 0.062 | -0.015 | 0.223 | 0.028 | 0.037 | 0.124 | 0.078 | -0.020 | 0.056 | 0.022 | 0.466 | -0.019 | 0.020 | -0.112 | 0.003 |
| DSS028 | 0.743 | 0.033 | 0.070 | 0.057 | 0.002 | -0.046 | -0.105 | -0.002 | -0.062 | -0.130 | 0.011 | 0.081 | 0.071 | 0.049 | 0.002 | 0.053 | -0.012 |
| DSS029 | 0.145 | 0.071 | 0.049 | -0.023 | -0.603 | -0.016 | 0.023 | -0.026 | 0.026 | 0.026 | -0.135 | -0.073 | 0.022 | -0.046 | 0.073 | 0.099 | 0.108 |
| DSS030 | 0.412 | 0.009 | 0.055 | 0.011 | 0.075 | 0.140 | -0.066 | 0.139 | -0.025 | -0.029 | 0.007 | 0.408 | 0.012 | 0.008 | 0.035 | 0.079 | 0.031 |
| DSS031 | 0.579 | 0.040 | 0.083 | 0.083 | 0.081 | 0.033 | -0.035 | 0.003 | 0.004 | -0.027 | -0.042 | 0.055 | -0.097 | 0.047 | -0.064 | -0.046 | -0.124 |
| DSS032 | 0.354 | -0.010 | 0.105 | 0.055 | 0.077 | 0.041 | -0.048 | -0.007 | 0.019 | 0.070 | 0.102 | 0.119 | -0.249 | 0.041 | -0.051 | -0.060 | -0.071 |
| DSS033 | -0.071 | 0.110 | 0.012 | 0.050 | -0.531 | -0.068 | -0.103 | -0.045 | 0.089 | 0.115 | -0.107 | 0.052 | -0.051 | -0.015 | -0.051 | 0.031 | 0.428 |
| DSS034 | 0.810 | 0.044 | 0.007 | 0.008 | -0.035 | -0.068 | 0.048 | 0.131 | -0.045 | -0.097 | -0.065 | 0.317 | -0.035 | -0.085 | 0.025 | 0.040 | 0.007 |
| DSS035 | 0.545 | 0.101 | -0.099 | 0.141 | -0.099 | -0.047 | 0.032 | 0.072 | -0.045 | 0.116 | -0.045 | 0.129 | -0.065 | 0.031 | -0.031 | 0.040 | 0.179 |
| DSS036 | 0.885 | -0.001 | 0.014 | 0.032 | -0.147 | -0.068 | -0.052 | -0.136 | -0.063 | 0.045 | -0.049 | 0.081 | -0.118 | -0.032 | 0.018 | 0.193 | 0.000 |
| DSS037 | 0.114 | 0.070 | 0.697 | -0.015 | -0.023 | -0.029 | -0.008 | 0.007 | -0.019 | 0.081 | -0.038 | 0.040 | 0.035 | -0.019 | 0.033 | -0.002 | 0.068 |
| DSS038 | 0.084 | -0.070 | 0.705 | 0.066 | -0.046 | -0.022 | -0.001 | 0.030 | 0.008 | 0.050 | 0.044 | -0.023 | -0.002 | 0.030 | 0.020 | -0.021 | -0.071 |
| DSS039 | 0.120 | 0.055 | 0.532 | -0.044 | 0.061 | 0.099 | 0.003 | -0.056 | 0.016 | 0.040 | 0.003 | 0.107 | 0.027 | 0.049 | 0.024 | 0.026 | 0.000 |
| DSS040 | -0.004 | 0.043 | 0.790 | 0.005 | -0.062 | 0.012 | -0.009 | 0.003 | 0.011 | 0.040 | 0.019 | 0.013 | 0.032 | 0.017 | -0.001 | 0.042 | 0.007 |
| DSS041 | 0.165 | 0.049 | 0.657 | 0.056 | 0.009 | -0.046 | 0.036 | 0.029 | 0.024 | -0.090 | 0.070 | -0.029 | 0.054 | -0.028 | 0.046 | 0.013 | -0.072 |
| DSS042 | 0.177 | 0.066 | 0.644 | -0.056 | -0.014 | 0.007 | 0.041 | -0.056 | 0.017 | 0.060 | -0.023 | -0.001 | -0.037 | 0.000 | -0.050 | -0.023 | 0.042 |
| DSS043 | 0.510 | 0.044 | 0.202 | -0.035 | -0.068 | -0.056 | -0.014 | 0.028 | -0.024 | 0.116 | -0.003 | -0.017 | 0.009 | 0.017 | 0.024 | -0.085 | 0.000 |
| DSS044 | -0.035 | -0.032 | 0.857 | 0.035 | -0.022 | -0.024 | -0.009 | 0.045 | 0.005 | 0.028 | 0.018 | 0.029 | 0.043 | -0.035 | 0.036 | -0.025 | 0.021 |
| DSS045 | 0.127 | 0.011 | 0.824 | -0.008 | -0.007 | -0.020 | 0.031 | -0.035 | -0.014 | -0.056 | 0.031 | 0.021 | -0.017 | -0.012 | 0.007 | 0.012 | -0.013 |
| DSS046 | 0.201 | 0.107 | -0.020 | 0.512 | 0.007 | 0.022 | 0.018 | -0.090 | 0.001 | -0.048 | 0.030 | 0.085 | -0.124 | 0.150 | -0.042 | 0.012 | -0.011 |
| DSS047 | -0.010 | 0.044 | 0.025 | 0.629 | -0.005 | -0.083 | -0.004 | -0.073 | 0.014 | 0.033 | -0.011 | -0.023 | 0.003 | 0.435 | -0.063 | -0.013 | 0.065 |
| DSS048 | 0.029 | -0.050 | 0.021 | 0.434 | 0.065 | -0.006 | 0.049 | -0.002 | 0.012 | 0.119 | -0.026 | -0.062 | 0.013 | 0.525 | 0.037 | 0.031 | 0.062 |
| DSS049 | 0.089 | 0.049 | -0.064 | 0.736 | 0.023 | -0.022 | -0.049 | -0.069 | 0.039 | -0.029 | -0.040 | -0.030 | 0.004 | 0.129 | 0.008 | 0.040 | -0.023 |
| DSS050 | -0.023 | -0.030 | 0.031 | 0.674 | -0.018 | 0.169 | 0.036 | -0.008 | -0.041 | -0.039 | 0.079 | -0.041 | -0.022 | -0.100 | -0.005 | -0.120 | -0.019 |
| DSS051 | 0.081 | 0.065 | -0.023 | 0.837 | -0.002 | -0.150 | -0.021 | -0.019 | -0.007 | -0.062 | 0.020 | -0.056 | 0.012 | -0.002 | 0.019 | -0.012 | 0.060 |
| DSS052 | 0.090 | 0.059 | -0.048 | 0.478 | 0.004 | 0.049 | -0.010 | 0.017 | -0.015 | 0.070 | -0.081 | 0.014 | -0.113 | 0.413 | 0.007 | 0.023 | 0.020 |
| DSS053 | -0.110 | -0.062 | 0.008 | 0.856 | -0.069 | 0.059 | 0.056 | 0.093 | -0.013 | -0.065 | -0.026 | -0.059 | 0.051 | 0.024 | 0.078 | -0.013 | -0.029 |
| DSS054 | -0.141 | -0.104 | 0.101 | 0.789 | -0.018 | -0.028 | -0.012 | 0.068 | -0.006 | -0.073 | -0.051 | -0.012 | -0.003 | 0.360 | 0.039 | 0.055 | -0.060 |
| DSS055 | 0.537 | 0.103 | 0.015 | -0.072 | 0.069 | 0.069 | -0.131 | -0.155 | -0.022 | 0.097 | -0.013 | -0.070 | 0.006 | -0.081 | 0.011 | 0.120 | -0.041 |
| DSS056 | -0.209 | -0.013 | -0.031 | -0.008 | -0.195 | -0.008 | 0.529 | 0.061 | 0.060 | -0.011 | -0.055 | -0.055 | 0.099 | 0.026 | 0.015 | -0.144 | 0.011 |
| DSS057 | 0.340 | 0.084 | 0.102 | 0.027 | 0.081 | 0.151 | 0.004 | 0.029 | 0.013 | -0.114 | -0.002 | 0.179 | 0.078 | 0.104 | -0.082 | 0.238 | 0.086 |
| DSS058 | 0.754 | 0.023 | -0.008 | -0.007 | 0.053 | 0.009 | 0.047 | -0.083 | 0.060 | -0.088 | 0.050 | 0.031 | 0.073 | 0.050 | -0.067 | 0.000 | -0.003 |
| DSS059 | 0.385 | 0.085 | 0.066 | -0.043 | 0.059 | 0.035 | 0.279 | 0.008 | -0.048 | 0.151 | -0.130 | 0.107 | -0.011 | 0.078 | -0.003 | 0.087 | 0.046 |
| DSS060 | 0.557 | 0.007 | 0.019 | 0.000 | -0.025 | 0.032 | 0.110 | -0.013 | 0.017 | 0.076 | 0.077 | 0.087 | 0.115 | 0.042 | -0.005 | 0.099 | 0.129 |
| DSS061 | 0.350 | 0.036 | -0.085 | -0.076 | 0.165 | 0.088 | -0.009 | 0.149 | 0.020 | 0.131 | 0.020 | 0.032 | -0.057 | 0.152 | -0.054 | 0.075 | -0.025 |
| DSS062 | 0.217 | 0.018 | 0.080 | 0.026 | 0.144 | 0.064 | 0.817 | -0.100 | -0.115 | -0.024 | -0.014 | -0.013 | -0.034 | -0.008 | -0.044 | 0.145 | -0.046 |
| DSS063 | -0.061 | -0.072 | -0.021 | 0.024 | -0.162 | 0.018 | 0.792 | -0.021 | -0.033 | -0.059 | 0.053 | -0.085 | 0.066 | 0.044 | 0.012 | -0.012 | -0.058 |
| DSS064 | 0.541 | 0.222 | -0.055 | -0.004 | -0.001 | -0.070 | 0.004 | -0.122 | 0.064 | 0.007 | 0.113 | -0.042 | 0.109 | 0.039 | -0.119 | -0.146 | -0.033 |
| DSS065 | 0.392 | 0.237 | -0.075 | -0.066 | 0.047 | -0.001 | -0.013 | 0.022 | 0.071 | 0.034 | 0.247 | 0.023 | 0.090 | -0.036 | -0.017 | -0.076 | -0.113 |
| DSS066 | 0.561 | 0.066 | -0.019 | 0.028 | 0.051 | -0.095 | -0.050 | 0.005 | 0.057 | -0.068 | 0.033 | 0.173 | 0.090 | 0.095 | 0.020 | -0.124 | -0.097 |
| DSS067 | 0.049 | 0.292 | -0.034 | -0.006 | 0.064 | 0.005 | 0.034 | 0.030 | 0.008 | 0.077 | 0.403 | -0.050 | 0.032 | -0.089 | -0.013 | -0.012 | -0.097 |
| DSS068 | 0.472 | 0.308 | 0.027 | -0.051 | -0.033 | 0.008 | -0.011 | 0.013 | 0.009 | -0.049 | 0.012 | -0.032 | 0.103 | 0.031 | -0.044 | -0.091 | -0.054 |
| DSS069 | 0.027 | 0.219 | -0.020 | 0.102 | 0.103 | 0.016 | -0.020 | -0.061 | 0.030 | 0.043 | 0.419 | -0.022 | -0.020 | -0.055 | 0.048 | 0.022 | -0.088 |
| DSS070 | 0.726 | 0.181 | -0.098 | -0.010 | -0.025 | -0.004 | -0.011 | -0.056 | 0.046 | -0.146 | 0.110 | 0.064 | 0.119 | 0.014 | 0.112 | -0.033 | 0.000 |
| DSS071 | -0.043 | 0.131 | 0.015 | 0.014 | 0.325 | -0.053 | 0.035 | 0.009 | -0.051 | 0.035 | 0.345 | -0.030 | 0.061 | 0.005 | -0.057 | -0.039 | -0.048 |
| DSS072 | 0.567 | 0.195 | -0.021 | -0.101 | 0.005 | -0.067 | 0.016 | -0.110 | 0.004 | 0.118 | 0.051 | -0.007 | 0.041 | -0.032 | -0.025 | -0.170 | 0.104 |
| DSS073 | 0.177 | 0.010 | 0.287 | -0.061 | 0.140 | -0.083 | 0.062 | -0.027 | 0.046 | 0.417 | -0.080 | 0.005 | -0.136 | -0.035 | 0.068 | -0.053 | -0.080 |
| DSS074 | 0.255 | -0.016 | 0.063 | -0.021 | -0.062 | -0.060 | -0.004 | 0.099 | 0.053 | 0.543 | 0.013 | 0.016 | 0.003 | 0.042 | -0.065 | -0.049 | 0.004 |
| DSS075 | 0.290 | -0.002 | 0.033 | 0.051 | 0.003 | 0.021 | 0.030 | 0.115 | 0.011 | 0.286 | 0.058 | 0.005 | 0.212 | 0.013 | -0.045 | -0.021 | -0.047 |
| DSS076 | 0.553 | 0.018 | 0.020 | 0.045 | -0.006 | 0.036 | 0.003 | -0.017 | -0.034 | 0.094 | 0.008 | -0.037 | 0.092 | 0.054 | -0.019 | 0.007 | -0.003 |
| DSS077 | 0.496 | 0.037 | 0.017 | 0.013 | -0.119 | 0.013 | -0.062 | -0.046 | -0.063 | 0.285 | 0.036 | -0.044 | 0.073 | 0.017 | 0.006 | 0.079 | -0.010 |
| DSS078 | 0.408 | -0.205 | 0.096 | 0.067 | 0.137 | -0.022 | -0.081 | -0.043 | -0.081 | 0.156 | 0.048 | -0.151 | 0.098 | -0.038 | -0.029 | 0.046 | -0.148 |
| DSS079 | 0.045 | -0.114 | 0.114 | -0.076 | 0.067 | -0.058 | -0.031 | 0.067 | 0.019 | 0.717 | 0.013 | -0.008 | -0.030 | 0.072 | 0.108 | 0.033 | 0.036 |
| DSS080 | 0.367 | 0.006 | 0.099 | -0.030 | -0.138 | -0.011 | -0.035 | -0.082 | 0.005 | 0.421 | -0.007 | 0.010 | 0.034 | 0.084 | -0.061 | 0.119 | 0.119 |
| DSS081 | 0.242 | -0.014 | 0.117 | 0.037 | 0.034 | 0.042 | -0.039 | 0.011 | 0.010 | 0.473 | -0.088 | -0.053 | 0.051 | -0.015 | -0.035 | -0.006 | 0.012 |
| DSS082 | 0.358 | 0.177 | 0.012 | -0.050 | -0.084 | 0.023 | 0.045 | -0.019 | -0.039 | 0.098 | 0.276 | 0.049 | -0.124 | 0.051 | 0.108 | -0.085 | 0.078 |
| DSS083 | 0.283 | 0.304 | 0.018 | -0.033 | -0.087 | 0.074 | 0.017 | -0.057 | 0.046 | 0.047 | 0.244 | 0.020 | -0.095 | 0.071 | 0.113 | 0.004 | 0.012 |
| DSS084 | 0.644 | 0.081 | 0.037 | 0.011 | -0.021 | -0.052 | -0.010 | 0.111 | -0.057 | 0.003 | 0.039 | 0.106 | -0.043 | -0.049 | 0.007 | -0.077 | 0.035 |
| DSS085 | 0.578 | 0.034 | -0.045 | -0.047 | 0.025 | 0.047 | -0.036 | 0.161 | 0.015 | -0.009 | 0.057 | 0.048 | 0.059 | -0.014 | -0.048 | 0.030 | 0.165 |
| DSS086 | 0.190 | 0.201 | -0.023 | 0.109 | -0.037 | -0.051 | -0.050 | 0.055 | -0.003 | -0.053 | 0.409 | 0.040 | 0.043 | -0.016 | 0.316 | -0.014 | 0.082 |
| DSS087 | 0.058 | 0.149 | -0.042 | 0.283 | 0.017 | -0.014 | 0.094 | 0.050 | -0.046 | 0.087 | 0.105 | 0.090 | -0.035 | -0.077 | 0.006 | 0.022 | 0.042 |
| DSS088 | 0.532 | 0.080 | -0.044 | 0.051 | 0.011 | -0.028 | 0.042 | 0.048 | 0.013 | -0.023 | 0.147 | -0.052 | 0.012 | 0.071 | 0.024 | -0.073 | 0.044 |
| DSS089 | 0.015 | 0.409 | 0.092 | 0.043 | 0.002 | -0.025 | 0.003 | 0.059 | 0.019 | 0.101 | 0.073 | 0.073 | -0.004 | 0.018 | 0.414 | 0.008 | -0.025 |
| DSS090 | 0.532 | 0.038 | -0.069 | 0.058 | -0.002 | 0.079 | -0.027 | 0.030 | -0.058 | 0.060 | 0.057 | -0.165 | -0.064 | 0.081 | 0.059 | 0.024 | 0.022 |
| DSS091 | 0.284 | 0.167 | 0.169 | -0.094 | 0.023 | 0.013 | -0.071 | -0.095 | -0.058 | -0.051 | 0.357 | -0.022 | -0.028 | 0.041 | 0.149 | 0.095 | 0.091 |
| DSS092 | 0.628 | 0.069 | 0.001 | 0.000 | -0.059 | -0.031 | -0.017 | -0.108 | -0.023 | -0.115 | 0.298 | -0.008 | 0.000 | 0.063 | 0.022 | -0.011 | 0.115 |
| DSS093 | 0.214 | 0.177 | 0.045 | -0.005 | 0.105 | -0.025 | 0.011 | -0.055 | 0.000 | -0.059 | 0.468 | 0.093 | 0.031 | -0.057 | 0.018 | -0.037 | 0.031 |
| DSS094 | 0.055 | -0.001 | 0.007 | 0.238 | -0.005 | 0.484 | -0.035 | 0.074 | 0.012 | 0.035 | -0.079 | 0.030 | 0.001 | -0.053 | 0.002 | -0.149 | 0.020 |
| DSS095 | 0.124 | -0.083 | 0.026 | 0.135 | -0.016 | 0.621 | -0.003 | -0.024 | 0.005 | 0.010 | 0.049 | -0.004 | 0.010 | -0.054 | 0.043 | -0.082 | 0.058 |
| DSS096 | 0.557 | 0.086 | -0.086 | 0.074 | 0.020 | 0.122 | -0.041 | 0.004 | -0.035 | -0.120 | 0.114 | -0.081 | 0.027 | 0.038 | 0.012 | 0.044 | 0.033 |
| DSS097 | 0.549 | 0.047 | 0.104 | -0.027 | 0.062 | 0.109 | 0.000 | -0.031 | -0.021 | 0.061 | -0.072 | 0.040 | -0.129 | 0.020 | -0.054 | -0.061 | 0.000 |
| DSS098 | 0.354 | 0.068 | -0.011 | -0.005 | 0.012 | 0.215 | -0.114 | 0.030 | -0.022 | 0.070 | 0.073 | -0.029 | 0.009 | -0.125 | 0.071 | -0.035 | 0.020 |
| DSS099 | 0.098 | -0.026 | 0.056 | 0.282 | 0.001 | 0.310 | -0.041 | 0.060 | -0.092 | 0.068 | -0.038 | 0.018 | -0.020 | 0.049 | 0.110 | -0.089 | -0.013 |
| DSS100 | -0.050 | 0.084 | -0.032 | -0.064 | -0.014 | 0.896 | 0.027 | 0.046 | 0.058 | -0.028 | -0.077 | 0.115 | -0.011 | -0.008 | -0.072 | 0.082 | -0.009 |
| DSS101 | -0.016 | 0.033 | -0.043 | 0.007 | -0.021 | 0.810 | 0.081 | 0.058 | 0.028 | -0.149 | 0.019 | 0.045 | 0.065 | 0.018 | -0.018 | 0.167 | -0.079 |
| DSS102 | 0.037 | 0.042 | 0.124 | 0.250 | -0.037 | 0.296 | -0.079 | -0.112 | 0.017 | 0.031 | 0.058 | -0.020 | -0.021 | -0.175 | -0.069 | 0.035 | 0.120 |
| DSS103 | 0.409 | 0.333 | -0.024 | -0.066 | 0.009 | 0.035 | 0.004 | -0.008 | 0.018 | -0.060 | 0.231 | -0.002 | -0.134 | 0.003 | -0.023 | -0.008 | -0.101 |
| DSS104 | 0.441 | 0.057 | -0.059 | 0.060 | 0.035 | -0.003 | -0.068 | -0.033 | 0.078 | 0.206 | -0.028 | -0.024 | 0.147 | 0.045 | 0.131 | 0.111 | -0.073 |
| DSS105 | 0.513 | 0.035 | 0.018 | -0.053 | 0.044 | 0.019 | -0.039 | 0.033 | -0.017 | 0.040 | 0.196 | 0.031 | 0.012 | -0.029 | 0.030 | 0.012 | -0.071 |
| DSS106 | -0.043 | 0.560 | -0.051 | 0.109 | -0.026 | 0.007 | -0.012 | 0.022 | 0.008 | 0.042 | 0.209 | 0.161 | -0.004 | -0.053 | -0.009 | 0.167 | -0.012 |
| DSS107 | 0.249 | 0.303 | -0.060 | 0.100 | -0.011 | -0.064 | -0.044 | 0.133 | -0.012 | 0.063 | -0.004 | 0.016 | 0.063 | 0.037 | 0.272 | 0.039 | -0.098 |
| DSS108 | 0.118 | 0.350 | -0.052 | 0.274 | -0.003 | -0.080 | 0.033 | 0.009 | 0.019 | -0.026 | 0.194 | 0.171 | -0.018 | -0.001 | 0.054 | 0.143 | 0.069 |
| DSS109 | 0.156 | 0.478 | 0.104 | 0.062 | -0.004 | -0.046 | 0.045 | 0.068 | 0.068 | -0.030 | 0.059 | -0.004 | 0.025 | 0.052 | 0.230 | 0.026 | -0.110 |
| DSS110 | -0.089 | -0.248 | 0.032 | -0.069 | -0.228 | -0.042 | -0.112 | 0.030 | -0.058 | -0.183 | 0.030 | -0.009 | -0.085 | 0.013 | -0.132 | -0.126 | 0.048 |
| DSS111 | 0.218 | -0.133 | -0.034 | -0.114 | -0.358 | -0.007 | -0.063 | 0.020 | 0.087 | -0.016 | -0.209 | -0.017 | -0.058 | 0.170 | -0.021 | 0.028 | 0.019 |
| DSS112 | 0.730 | 0.052 | 0.003 | -0.007 | -0.104 | 0.013 | 0.054 | -0.030 | 0.029 | 0.035 | 0.063 | 0.012 | -0.102 | -0.020 | 0.060 | 0.136 | -0.066 |
| DSS113 | 0.842 | 0.030 | -0.058 | 0.000 | -0.126 | -0.010 | 0.055 | -0.067 | -0.031 | 0.092 | -0.052 | 0.031 | -0.079 | -0.038 | 0.037 | 0.230 | -0.060 |
| DSS114 | -0.029 | -0.085 | 0.018 | 0.010 | -0.630 | 0.025 | 0.063 | 0.080 | 0.093 | 0.027 | 0.102 | 0.027 | -0.175 | -0.085 | 0.054 | 0.011 | -0.145 |
| DSS115 | 0.700 | -0.023 | -0.087 | 0.035 | 0.050 | -0.082 | -0.009 | 0.017 | 0.042 | 0.115 | 0.063 | 0.287 | -0.050 | -0.124 | 0.007 | 0.088 | 0.006 |
| DSS116 | 0.744 | -0.034 | 0.030 | -0.070 | -0.006 | -0.064 | 0.037 | -0.064 | -0.037 | -0.089 | 0.271 | -0.048 | 0.052 | -0.011 | 0.012 | 0.045 | 0.096 |
| DSS117 | 0.794 | 0.022 | -0.036 | 0.030 | -0.116 | 0.034 | 0.043 | -0.131 | -0.030 | 0.091 | -0.033 | 0.024 | -0.146 | -0.007 | 0.050 | 0.414 | -0.041 |
| DSS118 | 0.580 | -0.077 | 0.003 | -0.085 | 0.027 | 0.023 | 0.025 | -0.033 | -0.090 | 0.023 | 0.214 | -0.015 | 0.022 | 0.095 | 0.017 | 0.023 | 0.310 |
| DSS119 | 0.050 | -0.014 | -0.032 | 0.016 | -0.410 | 0.037 | 0.029 | -0.057 | 0.166 | -0.125 | 0.022 | -0.002 | -0.005 | 0.037 | 0.035 | -0.096 | 0.333 |
| DSS120 | 0.031 | 0.075 | 0.007 | 0.073 | -0.598 | -0.007 | 0.071 | 0.054 | 0.156 | -0.041 | -0.072 | -0.076 | -0.019 | -0.030 | -0.004 | -0.031 | 0.100 |
| DSS121 | 0.700 | 0.037 | 0.007 | 0.012 | 0.025 | -0.012 | -0.006 | 0.003 | 0.106 | -0.018 | 0.049 | -0.041 | -0.073 | 0.002 | -0.097 | 0.073 | -0.032 |
| DSS122 | 0.687 | -0.069 | 0.010 | 0.031 | 0.088 | 0.037 | 0.036 | 0.011 | 0.264 | -0.100 | 0.037 | -0.056 | 0.065 | -0.011 | -0.045 | 0.076 | -0.023 |
| DSS123 | 0.556 | -0.101 | 0.021 | 0.085 | 0.086 | 0.021 | 0.070 | 0.004 | 0.367 | -0.005 | 0.035 | -0.053 | -0.033 | -0.092 | -0.012 | 0.104 | -0.104 |
| DSS124 | 0.597 | -0.029 | 0.039 | 0.008 | 0.022 | -0.017 | 0.059 | 0.148 | 0.139 | -0.014 | 0.032 | -0.137 | 0.029 | -0.022 | -0.005 | 0.038 | 0.010 |
| DSS125 | 0.087 | -0.052 | -0.013 | 0.161 | 0.071 | 0.010 | 0.000 | 0.271 | 0.126 | 0.104 | 0.067 | 0.209 | 0.058 | 0.069 | 0.018 | 0.137 | 0.086 |
| DSS126 | 0.457 | -0.035 | 0.098 | -0.060 | 0.127 | -0.003 | 0.056 | -0.020 | 0.190 | 0.128 | -0.055 | -0.127 | -0.029 | 0.061 | 0.124 | 0.059 | 0.009 |
| DSS127 | 0.432 | 0.023 | 0.097 | 0.121 | 0.106 | -0.084 | -0.127 | 0.069 | 0.065 | 0.001 | -0.104 | -0.084 | -0.089 | 0.024 | -0.078 | 0.047 | 0.004 |
| DSS128 | -0.070 | 0.010 | -0.009 | 0.003 | -0.317 | 0.010 | -0.091 | 0.032 | 0.639 | 0.068 | -0.003 | 0.035 | 0.063 | 0.001 | -0.008 | 0.008 | 0.131 |
| DSS129 | -0.038 | 0.013 | 0.026 | -0.034 | -0.268 | 0.071 | -0.059 | -0.057 | 0.765 | 0.026 | -0.053 | -0.022 | 0.062 | 0.041 | 0.037 | -0.060 | 0.029 |
| DSS130 | 0.698 | 0.039 | -0.042 | -0.016 | -0.024 | -0.025 | 0.065 | 0.204 | -0.036 | -0.088 | 0.032 | -0.046 | 0.030 | 0.037 | -0.057 | -0.011 | 0.061 |
| DSS131 | 0.697 | -0.033 | 0.000 | 0.044 | -0.003 | -0.028 | -0.017 | 0.222 | 0.011 | -0.092 | -0.041 | -0.119 | -0.001 | 0.022 | -0.028 | 0.022 | 0.109 |
| DSS132 | 0.652 | 0.089 | 0.076 | -0.041 | -0.058 | -0.066 | -0.110 | 0.225 | -0.023 | -0.036 | -0.066 | -0.084 | -0.047 | -0.043 | -0.073 | 0.068 | 0.104 |
| DSS133 | 0.788 | 0.027 | 0.039 | 0.013 | -0.098 | -0.121 | 0.022 | 0.161 | -0.065 | -0.004 | 0.024 | -0.068 | -0.058 | -0.072 | -0.024 | 0.021 | 0.034 |
| DSS134 | 0.220 | 0.042 | -0.018 | 0.038 | -0.051 | 0.077 | -0.026 | 0.685 | -0.019 | 0.072 | -0.125 | 0.091 | 0.050 | 0.029 | 0.062 | -0.095 | -0.091 |
| DSS135 | 0.058 | -0.009 | -0.019 | 0.167 | 0.074 | 0.063 | -0.021 | 0.367 | -0.050 | -0.006 | 0.114 | 0.116 | 0.067 | 0.046 | 0.079 | 0.020 | -0.052 |
| DSS136 | 0.454 | 0.016 | -0.010 | -0.063 | 0.011 | -0.001 | 0.017 | 0.186 | 0.063 | 0.166 | 0.058 | 0.047 | -0.014 | 0.018 | -0.027 | 0.006 | 0.144 |
| DSS137 | 0.745 | -0.058 | -0.010 | 0.053 | -0.019 | -0.057 | 0.067 | 0.182 | -0.090 | 0.012 | -0.057 | -0.032 | -0.056 | -0.086 | -0.057 | 0.001 | -0.038 |
| DSS138 | 0.268 | 0.008 | 0.034 | 0.024 | -0.052 | 0.020 | -0.044 | 0.644 | -0.016 | 0.060 | -0.048 | 0.104 | 0.126 | -0.055 | 0.058 | -0.072 | -0.043 |

## DSS 1^st^ Order Constructs – Reliability and Validity Assessment

**Table 6.** Factor Loadings for 1^st^ Order DSS Constructs and Criterion Variables

| *Factors and Items* | *Loadings* |
| --- | --- |
| 1. **Complexity** 2. I often find it too complicated to accomplish a task using the ICT that are available to me at work. 3. I often need more time than expected to accomplish a task using the ICT that are available to me at work. 4. I feel that the ICT that are available to me at work are too confusing. 5. I often do not find enough time to keep up with new functionalities of ICT at work. 6. It would take me too long to completely figure out how to use the ICT that are available to me at work. | .843  .822  .830  .799  .794 |
| 1. **Conflicts** 2. I feel that my private life suffers due to ICT enabling work-related problems to reach me everywhere. 3. It is too hard for me to keep my private life and work life separated due to ICT. 4. ICT make it harder to create clear boundaries between my private life and work life. 5. My work-life balance suffers due to ICT. 6. The ubiquity of ICT disturbs my work-life balance. | .860  .851  .830  .847  .839 |
| 1. **Insecurity** 2. I feel that my job position is threatened due to ICT. 3. I fear that I could be replaced at work due to the increasing standardization of work processes, which is enabled by ICT. 4. I cannot be optimistic about my long-term job security because of the threat of ICT automatization. 5. I fear that I could be replaced by machines. 6. I fear that digitalization will cost me my job. | .874  .877  .861  .861  .858 |
| 1. **Invasion (of Privacy)** 2. I fear that my use of ICT is less confidential than I would like to. 3. I fear that the information that I exchange using ICT is not as protected as I would like to. 4. I fear that malevolent outsiders (e.g., hackers) can easily copy my identity due to ICT. 5. My personal information is too easily accessible due to ICT. 6. I fear that my personal data can easily be stolen by others online. | .803  .807  .794  .814  .775 |
| 1. **Overload** 2. Due to ICT I have too much to do. 3. Due to ICT I have a too large variety of different things to do at work. 4. ICT make it too easy for other individuals to send me additional work. 5. I never have any spare time, because my schedule is too tightly organized by ICT. 6. There is a constant surge of work-related information coming in through ICT that I just cannot keep up with. | .849  .808  .662  .814  .808 |
| 1. **Safety** 2. I have to worry too often, whether I might download malicious programs. 3. I have to worry too often, whether I might receive malicious e-mails. 4. I fear that hackers might get access to company secrets through a mistake of mine. 5. I feel anxious when I get an e-mail from somebody that I do not know as it could be a malevolent attack. 6. E-Mails whose sender I do not know make me nervous. | .808  .836  .730  .814  .749 |
| 1. **Social Environment** 2. Due to ICT I have too much to do with the problems of others. 3. I think that ICT generate too much of an expectation that I have to be reachable everywhere and at any time. 4. Too much time gets lost at work because of irrelevant communication with other people on social media. 5. I feel that ICT create unwanted social norms (e.g., the expectation that e-mails should be answered right away). 6. It is too hard to take a break from social interactions at work due to the communication possibilities of ICT. | .783  .745  .655  .738  .779 |
| 1. **Technical Support** 2. I have to worry about ICT-related problems as our organization does not offer enough support for their removal. 3. In the case of ICT-related problems, it happens too often that there is not enough support available at work. 4. I think that it happens too often that technical support is not available when I need it. 5. I often have to wait for a long time because technical problems cannot be adequately solved in our organization. 6. I fear that a technical problem I have at work could not be solved by anyone else at work. | .826  .826  .769  .825  .640 |
| 1. **Usefulness** 2. I think that the demands of my work and the functions provided by the ICT I use do not fit sufficiently. 3. I think that I do not gain enough benefits from using the ICT that I am provided with at work for my tasks. 4. The ICT I use at work are full of too many functionalities that I never need. 5. It requires too many different systems to fulfill the tasks that I have to do during an average day at work. 6. I think that most of the ICT I am supplied with at work is not useful enough and I could work without it. | .829  .808  .708  .752  .794 |
| 1. **Unreliability** 2. I think that I am too often confronted with unexpected behavior of the ICT I use at work (e.g., breakdowns or long response times). 3. I think that I lose too much time due to technical malfunctions. 4. I think that I spend too much time trying to fix technical malfunctions. 5. There is just too much of my time at work wasted coping with the unreliability of ICT. 6. The daily hassles with ICT (e.g., slow programs or unexpected behavior) are really bothering me. | .817  .827  .801  .841  .783 |
| **A. Emotional Exhaustion**   1. I feel burned out from my work. 2. I feel used up at the end of the workday. 3. I feel emotionally drained from my work. 4. I feel fatigued when I get up in the morning and have to face another day on the job. 5. Working all day is really a strain for me. | .862  .825  .863  .849  .752 |
| **B. Innovation Climate**   1. In our organization we have a very open communications environment. 2. In our organization employees and functional managers are supportive of each other. 3. In our organization employees at all levels are rewarded for learning new skills. 4. Management encourages experimental mind-set and risk taking. 5. In our organization new ideas are easy to be implemented. | .835  .851  -  -  .687 |
| **C. Job Satisfaction**   1. I like doing the things I do at work. 2. I feel a sense of pride in doing my job. 3. My job is enjoyable. | .871  .815  .890 |
| **D. User Satisfaction**  How do you feel about your overall experience of utilizing ICT in connection with your work tasks?  Very dissatisfied/Very satisfied  Very displeased/Very pleased  Very frustrated/Very contented  Absolutely terrible/Absolutely delighted | .921  .922  .908  .855 |

Table 7 shows the loadings and crossloadings for the final set of items and the ten 1^st^ order constructs as well as the outcome variables included in the measurement model. A grey background indicates the items that belong to a specific construct (first row).

**Table 7.** Crossloadings for the ten 1^st^ order Constructs and the Outcome Variables

|  | I | II | III | IV | V | VI | VII | VIII | IX | X | A | B | C | D |
| --- | --- | --- | --- | --- | --- | --- | --- | --- | --- | --- | --- | --- | --- | --- |
| DSS001 | 0.843 | 0.542 | 0.579 | 0.418 | 0.649 | 0.491 | 0.576 | 0.719 | 0.636 | 0.672 | 0.438 | -0.068 | -0.189 | -0.348 |
| DSS002 | 0.822 | 0.525 | 0.539 | 0.426 | 0.655 | 0.468 | 0.580 | 0.671 | 0.606 | 0.651 | 0.452 | -0.110 | -0.191 | -0.300 |
| DSS003 | 0.830 | 0.466 | 0.564 | 0.387 | 0.595 | 0.433 | 0.524 | 0.678 | 0.589 | 0.617 | 0.409 | -0.071 | -0.184 | -0.314 |
| DSS005 | 0.799 | 0.512 | 0.505 | 0.453 | 0.605 | 0.446 | 0.541 | 0.630 | 0.554 | 0.587 | 0.425 | -0.059 | -0.168 | -0.288 |
| DSS007 | 0.794 | 0.402 | 0.499 | 0.363 | 0.525 | 0.416 | 0.450 | 0.602 | 0.497 | 0.530 | 0.360 | -0.063 | -0.171 | -0.289 |
| DSS013 | 0.481 | 0.860 | 0.455 | 0.486 | 0.635 | 0.439 | 0.663 | 0.517 | 0.489 | 0.516 | 0.529 | -0.059 | -0.222 | -0.245 |
| DSS014 | 0.499 | 0.851 | 0.432 | 0.406 | 0.626 | 0.382 | 0.668 | 0.507 | 0.445 | 0.518 | 0.497 | -0.041 | -0.226 | -0.250 |
| DSS015 | 0.450 | 0.830 | 0.394 | 0.480 | 0.600 | 0.389 | 0.657 | 0.487 | 0.450 | 0.506 | 0.492 | -0.043 | -0.183 | -0.225 |
| DSS016 | 0.550 | 0.874 | 0.520 | 0.473 | 0.681 | 0.421 | 0.661 | 0.562 | 0.527 | 0.560 | 0.551 | -0.103 | -0.232 | -0.272 |
| DSS018 | 0.575 | 0.839 | 0.512 | 0.481 | 0.674 | 0.435 | 0.656 | 0.584 | 0.544 | 0.581 | 0.503 | -0.072 | -0.217 | -0.260 |
| DSS037 | 0.599 | 0.502 | 0.874 | 0.371 | 0.551 | 0.443 | 0.494 | 0.567 | 0.528 | 0.555 | 0.390 | -0.043 | -0.230 | -0.247 |
| DSS040 | 0.543 | 0.469 | 0.877 | 0.372 | 0.521 | 0.433 | 0.454 | 0.529 | 0.509 | 0.538 | 0.352 | -0.039 | -0.236 | -0.255 |
| DSS042 | 0.599 | 0.496 | 0.861 | 0.362 | 0.547 | 0.440 | 0.487 | 0.550 | 0.533 | 0.554 | 0.391 | -0.071 | -0.221 | -0.263 |
| DSS044 | 0.524 | 0.424 | 0.858 | 0.343 | 0.479 | 0.429 | 0.408 | 0.497 | 0.476 | 0.497 | 0.310 | -0.034 | -0.179 | -0.172 |
| DSS045 | 0.591 | 0.471 | 0.884 | 0.350 | 0.533 | 0.430 | 0.463 | 0.541 | 0.519 | 0.537 | 0.351 | -0.020 | -0.186 | -0.215 |
| DSS046 | 0.479 | 0.514 | 0.385 | 0.803 | 0.509 | 0.525 | 0.574 | 0.500 | 0.483 | 0.487 | 0.381 | -0.067 | -0.173 | -0.227 |
| DSS049 | 0.399 | 0.446 | 0.311 | 0.807 | 0.441 | 0.505 | 0.510 | 0.427 | 0.440 | 0.415 | 0.323 | 0.007 | -0.102 | -0.155 |
| DSS050 | 0.395 | 0.403 | 0.366 | 0.794 | 0.424 | 0.614 | 0.480 | 0.399 | 0.419 | 0.408 | 0.304 | 0.037 | -0.085 | -0.136 |
| DSS051 | 0.389 | 0.453 | 0.313 | 0.814 | 0.435 | 0.490 | 0.532 | 0.409 | 0.447 | 0.460 | 0.313 | 0.021 | -0.077 | -0.141 |
| DSS053 | 0.296 | 0.321 | 0.249 | 0.775 | 0.316 | 0.519 | 0.399 | 0.328 | 0.362 | 0.340 | 0.272 | 0.011 | -0.037 | -0.102 |
| DSS064 | 0.635 | 0.626 | 0.528 | 0.435 | 0.849 | 0.415 | 0.628 | 0.619 | 0.573 | 0.617 | 0.503 | -0.103 | -0.244 | -0.285 |
| DSS065 | 0.563 | 0.602 | 0.460 | 0.430 | 0.808 | 0.427 | 0.641 | 0.591 | 0.562 | 0.574 | 0.483 | -0.042 | -0.192 | -0.204 |
| DSS067 | 0.389 | 0.515 | 0.357 | 0.410 | 0.662 | 0.365 | 0.562 | 0.450 | 0.438 | 0.455 | 0.356 | 0.003 | -0.140 | -0.118 |
| DSS068 | 0.637 | 0.627 | 0.550 | 0.424 | 0.814 | 0.439 | 0.634 | 0.594 | 0.572 | 0.611 | 0.510 | -0.082 | -0.232 | -0.244 |
| DSS070 | 0.669 | 0.617 | 0.477 | 0.460 | 0.808 | 0.446 | 0.642 | 0.656 | 0.593 | 0.616 | 0.491 | -0.065 | -0.196 | -0.260 |
| DSS094 | 0.468 | 0.419 | 0.432 | 0.570 | 0.443 | 0.808 | 0.445 | 0.428 | 0.460 | 0.470 | 0.344 | 0.028 | -0.123 | -0.153 |
| DSS095 | 0.507 | 0.414 | 0.462 | 0.558 | 0.478 | 0.836 | 0.463 | 0.460 | 0.502 | 0.512 | 0.332 | 0.006 | -0.120 | -0.191 |
| DSS099 | 0.494 | 0.424 | 0.453 | 0.558 | 0.470 | 0.730 | 0.454 | 0.439 | 0.424 | 0.443 | 0.303 | 0.049 | -0.093 | -0.130 |
| DSS100 | 0.393 | 0.362 | 0.343 | 0.479 | 0.358 | 0.814 | 0.399 | 0.379 | 0.435 | 0.415 | 0.324 | -0.038 | -0.110 | -0.187 |
| DSS101 | 0.313 | 0.296 | 0.278 | 0.448 | 0.337 | 0.749 | 0.345 | 0.344 | 0.397 | 0.358 | 0.292 | -0.039 | -0.070 | -0.187 |
| DSS103 | 0.578 | 0.630 | 0.486 | 0.468 | 0.690 | 0.462 | 0.783 | 0.608 | 0.574 | 0.615 | 0.519 | -0.061 | -0.231 | -0.245 |
| DSS106 | 0.391 | 0.589 | 0.307 | 0.487 | 0.543 | 0.355 | 0.745 | 0.465 | 0.401 | 0.440 | 0.416 | -0.029 | -0.161 | -0.202 |
| DSS107 | 0.472 | 0.481 | 0.366 | 0.391 | 0.506 | 0.358 | 0.655 | 0.466 | 0.424 | 0.459 | 0.370 | -0.004 | -0.144 | -0.174 |
| DSS108 | 0.410 | 0.534 | 0.289 | 0.547 | 0.509 | 0.371 | 0.738 | 0.480 | 0.446 | 0.467 | 0.444 | -0.038 | -0.179 | -0.220 |
| DSS109 | 0.561 | 0.626 | 0.498 | 0.460 | 0.632 | 0.421 | 0.779 | 0.543 | 0.517 | 0.544 | 0.499 | -0.059 | -0.201 | -0.229 |
| DSS112 | 0.673 | 0.541 | 0.535 | 0.438 | 0.631 | 0.464 | 0.576 | 0.829 | 0.649 | 0.662 | 0.484 | -0.163 | -0.230 | -0.338 |
| DSS113 | 0.657 | 0.480 | 0.497 | 0.393 | 0.558 | 0.407 | 0.526 | 0.808 | 0.586 | 0.597 | 0.415 | -0.119 | -0.217 | -0.361 |
| DSS115 | 0.599 | 0.463 | 0.402 | 0.452 | 0.537 | 0.384 | 0.537 | 0.708 | 0.526 | 0.554 | 0.393 | -0.026 | -0.113 | -0.278 |
| DSS116 | 0.626 | 0.519 | 0.503 | 0.386 | 0.654 | 0.382 | 0.569 | 0.752 | 0.593 | 0.645 | 0.437 | -0.083 | -0.196 | -0.259 |
| DSS117 | 0.596 | 0.433 | 0.463 | 0.395 | 0.512 | 0.386 | 0.512 | 0.794 | 0.577 | 0.563 | 0.400 | -0.073 | -0.188 | -0.349 |
| DSS121 | 0.641 | 0.549 | 0.519 | 0.470 | 0.645 | 0.477 | 0.594 | 0.679 | 0.826 | 0.699 | 0.483 | -0.132 | -0.230 | -0.263 |
| DSS122 | 0.585 | 0.464 | 0.463 | 0.439 | 0.588 | 0.470 | 0.533 | 0.632 | 0.826 | 0.680 | 0.438 | -0.135 | -0.183 | -0.264 |
| DSS123 | 0.486 | 0.367 | 0.404 | 0.420 | 0.444 | 0.420 | 0.434 | 0.547 | 0.769 | 0.583 | 0.375 | -0.138 | -0.136 | -0.267 |
| DSS124 | 0.567 | 0.465 | 0.496 | 0.421 | 0.567 | 0.450 | 0.515 | 0.591 | 0.825 | 0.711 | 0.446 | -0.103 | -0.178 | -0.289 |
| DSS127 | 0.463 | 0.395 | 0.423 | 0.379 | 0.447 | 0.376 | 0.413 | 0.467 | 0.640 | 0.520 | 0.335 | 0.024 | -0.093 | -0.127 |
| DSS130 | 0.614 | 0.522 | 0.485 | 0.450 | 0.628 | 0.464 | 0.589 | 0.628 | 0.666 | 0.817 | 0.461 | -0.102 | -0.184 | -0.305 |
| DSS131 | 0.581 | 0.484 | 0.478 | 0.457 | 0.557 | 0.474 | 0.538 | 0.606 | 0.694 | 0.827 | 0.436 | -0.054 | -0.163 | -0.320 |
| DSS132 | 0.604 | 0.509 | 0.520 | 0.403 | 0.573 | 0.433 | 0.535 | 0.620 | 0.677 | 0.801 | 0.452 | -0.064 | -0.190 | -0.324 |
| DSS133 | 0.676 | 0.542 | 0.569 | 0.454 | 0.643 | 0.460 | 0.593 | 0.685 | 0.697 | 0.841 | 0.461 | -0.083 | -0.228 | -0.349 |
| DSS136 | 0.575 | 0.505 | 0.461 | 0.420 | 0.574 | 0.445 | 0.541 | 0.614 | 0.623 | 0.783 | 0.494 | -0.096 | -0.226 | -0.319 |
| EMEX01 | 0.410 | 0.505 | 0.342 | 0.320 | 0.492 | 0.304 | 0.496 | 0.459 | 0.446 | 0.473 | 0.862 | -0.287 | -0.481 | -0.317 |
| EMEX02 | 0.424 | 0.523 | 0.329 | 0.359 | 0.508 | 0.342 | 0.515 | 0.471 | 0.462 | 0.467 | 0.825 | -0.246 | -0.343 | -0.278 |
| EMEX03 | 0.458 | 0.516 | 0.356 | 0.336 | 0.524 | 0.328 | 0.515 | 0.472 | 0.459 | 0.505 | 0.863 | -0.243 | -0.428 | -0.331 |
| EMEX04 | 0.438 | 0.508 | 0.390 | 0.353 | 0.491 | 0.378 | 0.527 | 0.463 | 0.459 | 0.483 | 0.849 | -0.238 | -0.469 | -0.290 |
| EMEX05 | 0.393 | 0.458 | 0.304 | 0.315 | 0.468 | 0.332 | 0.491 | 0.406 | 0.404 | 0.427 | 0.752 | -0.134 | -0.344 | -0.265 |
| INNO01 | -0.110 | -0.054 | -0.053 | -0.001 | -0.045 | -0.007 | -0.033 | -0.124 | -0.107 | -0.078 | -0.194 | 0.835 | 0.402 | 0.309 |
| INNO02 | -0.072 | -0.075 | -0.064 | 0.012 | -0.082 | 0.020 | -0.049 | -0.100 | -0.124 | -0.085 | -0.244 | 0.851 | 0.465 | 0.323 |
| INNO05 | -0.013 | -0.049 | 0.034 | -0.038 | -0.064 | -0.022 | -0.057 | -0.059 | -0.085 | -0.075 | -0.248 | 0.687 | 0.361 | 0.325 |
| JOSA01 | -0.208 | -0.216 | -0.192 | -0.107 | -0.214 | -0.111 | -0.219 | -0.215 | -0.200 | -0.225 | -0.422 | 0.431 | 0.871 | 0.393 |
| JOSA02 | -0.167 | -0.186 | -0.207 | -0.079 | -0.209 | -0.072 | -0.178 | -0.179 | -0.142 | -0.171 | -0.343 | 0.386 | 0.815 | 0.342 |
| JOSA03 | -0.194 | -0.250 | -0.228 | -0.139 | -0.241 | -0.152 | -0.245 | -0.236 | -0.214 | -0.231 | -0.505 | 0.507 | 0.890 | 0.445 |
| USSA01 | -0.389 | -0.280 | -0.282 | -0.191 | -0.292 | -0.197 | -0.285 | -0.405 | -0.315 | -0.380 | -0.344 | 0.362 | 0.453 | 0.921 |
| USSA02 | -0.336 | -0.264 | -0.243 | -0.167 | -0.265 | -0.202 | -0.260 | -0.373 | -0.288 | -0.353 | -0.322 | 0.359 | 0.411 | 0.922 |
| USSA03 | -0.342 | -0.281 | -0.244 | -0.187 | -0.279 | -0.194 | -0.283 | -0.379 | -0.303 | -0.389 | -0.325 | 0.362 | 0.413 | 0.908 |
| USSA04 | -0.284 | -0.235 | -0.191 | -0.171 | -0.199 | -0.185 | -0.216 | -0.308 | -0.234 | -0.306 | -0.294 | 0.329 | 0.379 | 0.855 |
| DSS000 = Items in the DSS; EMEX00 = Items of Emotional Exhaustion; INNO00 = Items of Innovation Climate; JOSA00 = Items of Job Satisfaction; USSA00 = Items of User Satisfaction  I = Complexity, II = Conflicts, III = Insecurity, IV = Invasion, V = Overload, VI = Safety, VII = Social Environment, VIII = Usefulness, IX = Technical Support, X = Unreliabilty  A = Emotional Exhaustion, B = Innovation Climate, C = Job Satisfaction, D = User Satisfaction | | | | | | | | | | | | | | |

**Table 8.** Discriminant Validity based on Fornell-Larcker Criterion for 1^st^ Order DSS Constructs and Criterion Variables

|  | 01 | 02 | 03 | 04 | 05 | 06 | 07 | 08 | 09 | 10 | A | B | C | D |
| --- | --- | --- | --- | --- | --- | --- | --- | --- | --- | --- | --- | --- | --- | --- |
| 01. Complexity | 0.818 |  |  |  |  |  |  |  |  |  |  |  |  |  |
| 02. Conflicts | 0.602 | 0.851 |  |  |  |  |  |  |  |  |  |  |  |  |
| 03. Insecurity | 0.658 | 0.545 | 0.871 |  |  |  |  |  |  |  |  |  |  |  |
| 04. Invasion | 0.501 | 0.547 | 0.414 | 0.799 |  |  |  |  |  |  |  |  |  |  |
| 05. Overload | 0.744 | 0.757 | 0.607 | 0.543 | 0.791 |  |  |  |  |  |  |  |  |  |
| 06. Safety | 0.553 | 0.486 | 0.500 | 0.663 | 0.529 | 0.789 |  |  |  |  |  |  |  |  |
| 07. Social Environment | 0.656 | 0.776 | 0.533 | 0.635 | 0.784 | 0.534 | 0.741 |  |  |  |  |  |  |  |
| 08. Technical Support | 0.750 | 0.631 | 0.618 | 0.536 | 0.732 | 0.559 | 0.688 | 0.814 |  |  |  |  |  |  |
| 09. Usefulness | 0.708 | 0.578 | 0.591 | 0.545 | 0.697 | 0.563 | 0.644 | 0.825 | 0.780 |  |  |  |  |  |
| 10. Unreliability | 0.809 | 0.625 | 0.619 | 0.527 | 0.742 | 0.520 | 0.696 | 0.776 | 0.754 | 0.779 |  |  |  |  |
| A. Emotional Exhaustion | 0.511 | 0.605 | 0.415 | 0.406 | 0.598 | 0.405 | 0.613 | 0.567 | 0.538 | 0.547 | 0.831 |  |  |  |
| B. Innovation Climate | -0.091 | -0.076 | -0.049 | -0.004 | -0.079 | 0.001 | -0.055 | -0.099 | -0.135 | -0.124 | -0.278 | 0.794 |  |  |
| C. Job Satisfaction | -0.221 | -0.255 | -0.244 | -0.128 | -0.258 | -0.132 | -0.251 | -0.245 | -0.218 | -0.246 | -0.498 | 0.517 | 0.859 |  |
| D. User Satisfaction | -0.377 | -0.295 | -0.268 | -0.199 | -0.289 | -0.216 | -0.291 | -0.398 | -0.318 | -0.409 | -0.357 | 0.392 | 0.461 | 0.902 |
| Values in italics indicate the square root of the AVE, while values below indicate the inter-construct correlations of the latent variable scores. | | | | | | | | | | | | | | |

**Table 9.** Discriminant Validity based on HTMT for 1^st^ Order DSS Constructs and Criterion Variables

|  | *01* | *02* | *03* | *04* | *05* | *06* | *07* | *08* | *09* | *10* | *A* | *B* | *C* |
| --- | --- | --- | --- | --- | --- | --- | --- | --- | --- | --- | --- | --- | --- |
| 01. Complexity | - |  |  |  |  |  |  |  |  |  |  |  |  |
| 02. Conflicts | 0.671 | - |  |  |  |  |  |  |  |  |  |  |  |
| 03. Insecurity | 0.729 | 0.592 | - |  |  |  |  |  |  |  |  |  |  |
| 04. Invasion | 0.563 | 0.606 | 0.455 | - |  |  |  |  |  |  |  |  |  |
| 05. Overload | 0.846 | 0.862* | 0.676 | 0.625 | - |  |  |  |  |  |  |  |  |
| 06. Safety | 0.639 | 0.555 | 0.565 | 0.778 | 0.625 | - |  |  |  |  |  |  |  |
| 07. Social Environment | 0.777 | 0.911 | 0.610 | 0.755 | 0.949 | 0.647 | - |  |  |  |  |  |  |
| 08. Technical Support | 0.854 | 0.708 | 0.686 | 0.609 | 0.844 | 0.649 | 0.818 | - |  |  |  |  |  |
| 09. Usefulness | 0.818 | 0.659 | 0.671 | 0.634 | 0.815 | 0.667 | 0.775 | 0.959 | - |  |  |  |  |
| 10. Unreliability | 0.942 | 0.718 | 0.699 | 0.611 | 0.875 | 0.617 | 0.850* | 0.906 | 0.891* | - |  |  |  |
| A. Emotional Exhaustion | 0.578 | 0.674 | 0.455 | 0.456 | 0.683 | 0.468 | 0.723 | 0.643 | 0.618 | 0.634 | - |  |  |
| B. Innovation Climate | 0.105 | 0.091 | 0.076 | 0.063 | 0.105 | 0.059 | 0.076 | 0.124 | 0.173 | 0.147 | 0.357 | - |  |
| C. Job Satisfaction | 0.260 | 0.293 | 0.277 | 0.143 | 0.303 | 0.154 | 0.304 | 0.286 | 0.251 | 0.290 | 0.577 | 0.662 | - |
| D. User Satisfaction | 0.416 | 0.321 | 0.285 | 0.213 | 0.315 | 0.243 | 0.335 | 0.441 | 0.350 | 0.460 | 0.393 | 0.490 | 0.524 |
| * Indicates values that fall below the .900 threshold, but do not fulfill the more conservative .850 threshold. | | | | | | | | | | | | | |

Table 10 shows the reliability and validity statistics for the original 15 stressors categories and outcome variables including all items (as specified in Table 2, for all 138 DSS items, and Table 6, for all items of the four outcome variables).

**Table 10.** Reliability and Convergent Validity Statistics for the Original 15 Stressor Categories and Outcome Variables

|  | I | II | III | IV | V | VI | VII | VIII | IX | X | XI | XII | XIII | XIV | XV | A | B | C | D |
| --- | --- | --- | --- | --- | --- | --- | --- | --- | --- | --- | --- | --- | --- | --- | --- | --- | --- | --- | --- |
| α | 0.910 | 0.929 | 0.738 | 0.773 | 0.939 | 0.903 | 0.694 | 0.893 | 0.880 | 0.906 | 0.877 | 0.629 | 0.730 | 0.819 | 0.897 | 0.887 | 0.799 | 0.822 | 0.923 |
| Rho | 0.914 | 0.932 | 0.826 | 0.818 | 0.942 | 0.913 | 0.782 | 0.912 | 0.905 | 0.915 | 0.881 | 0.849 | 0.765 | 0.829 | 0.908 | 0.890 | 0.800 | 0.830 | 0.928 |
| CR | 0.926 | 0.941 | 0.825 | 0.842 | 0.949 | 0.920 | 0.789 | 0.912 | 0.902 | 0.921 | 0.901 | 0.770 | 0.815 | 0.863 | 0.917 | 0.918 | 0.861 | 0.894 | 0.946 |
| AVE | 0.581 | 0.639 | 0.405 | 0.406 | 0.673 | 0.562 | 0.338 | 0.539 | 0.512 | 0.497 | 0.505 | 0.422 | 0.360 | 0.420 | 0.553 | 0.691 | 0.554 | 0.738 | 0.813 |
| α = Cronbach’s Alpha, with a threshold of >.700; Rho = Rho Alpha, with a threshold of >.700; CR = Composite Reliability, with a threshold of >.700; AVE = Average Variance Extracted, with a threshold of >.500  I = Complexity, II = Conflicts, III = Control, IV = Costs, V = Insecurity, VI = Invasion, VII = Involvement, VIII = Boredom, IX = Overload, X = Role Stress, XI = Safety, XII = Social Environment, XIII = Usefulness, XIV = Technical Support, XV = Unreliability  A = Emotional Exhaustion, B = Innovation Climate, C = Job Satisfaction, D = User Satisfaction | | | | | | | | | | | | | | | | | | | |

Table 11 shows the loadings and crossloadings for the initial set of items and the 15 1^st^ order constructs as well as the outcome variables included in the measurement model. A grey background indicates the items that belong to a specific construct (first row).

**Table 11.** Crossloadings for the 15 1^st^ order Constructs and the Outcome Variables

|  | I | II | III | IV | V | VI | VII | VIII | IX | X | XI | XII | XIII | XIV | XV | A | B | C | D |
| --- | --- | --- | --- | --- | --- | --- | --- | --- | --- | --- | --- | --- | --- | --- | --- | --- | --- | --- | --- |
| DSS001 | 0.817 | 0.582 | 0.660 | 0.697 | 0.622 | 0.454 | 0.600 | 0.666 | 0.658 | 0.676 | 0.602 | 0.634 | 0.665 | 0.570 | 0.675 | 0.438 | 0.001 | -0.189 | -0.347 |
| DSS002 | 0.792 | 0.567 | 0.633 | 0.667 | 0.586 | 0.457 | 0.556 | 0.625 | 0.670 | 0.673 | 0.574 | 0.629 | 0.607 | 0.544 | 0.645 | 0.452 | -0.041 | -0.191 | -0.299 |
| DSS003 | 0.801 | 0.510 | 0.623 | 0.671 | 0.599 | 0.412 | 0.580 | 0.626 | 0.617 | 0.621 | 0.556 | 0.572 | 0.625 | 0.527 | 0.606 | 0.409 | -0.016 | -0.184 | -0.314 |
| DSS004 | 0.764 | 0.517 | 0.589 | 0.644 | 0.517 | 0.467 | 0.595 | 0.557 | 0.593 | 0.623 | 0.553 | 0.580 | 0.661 | 0.597 | 0.642 | 0.414 | -0.083 | -0.161 | -0.335 |
| DSS005 | 0.776 | 0.540 | 0.613 | 0.674 | 0.545 | 0.474 | 0.555 | 0.589 | 0.622 | 0.635 | 0.536 | 0.572 | 0.578 | 0.510 | 0.599 | 0.425 | -0.012 | -0.169 | -0.288 |
| DSS006 | 0.743 | 0.525 | 0.592 | 0.640 | 0.529 | 0.459 | 0.547 | 0.584 | 0.615 | 0.639 | 0.537 | 0.606 | 0.575 | 0.479 | 0.589 | 0.391 | -0.005 | -0.135 | -0.262 |
| DSS007 | 0.773 | 0.440 | 0.544 | 0.643 | 0.536 | 0.391 | 0.539 | 0.558 | 0.550 | 0.554 | 0.498 | 0.496 | 0.573 | 0.451 | 0.535 | 0.360 | -0.042 | -0.171 | -0.289 |
| DSS008 | 0.702 | 0.447 | 0.539 | 0.585 | 0.475 | 0.432 | 0.512 | 0.493 | 0.521 | 0.520 | 0.495 | 0.490 | 0.505 | 0.455 | 0.544 | 0.360 | -0.015 | -0.138 | -0.290 |
| DSS009 | 0.684 | 0.437 | 0.543 | 0.548 | 0.473 | 0.438 | 0.495 | 0.499 | 0.533 | 0.518 | 0.461 | 0.520 | 0.481 | 0.434 | 0.539 | 0.333 | 0.037 | -0.085 | -0.214 |
| DSS010 | 0.565 | 0.757 | 0.585 | 0.562 | 0.517 | 0.508 | 0.500 | 0.584 | 0.653 | 0.676 | 0.539 | 0.692 | 0.477 | 0.458 | 0.569 | 0.446 | 0.021 | -0.227 | -0.216 |
| DSS011 | 0.444 | 0.772 | 0.521 | 0.493 | 0.449 | 0.455 | 0.422 | 0.494 | 0.597 | 0.592 | 0.488 | 0.633 | 0.398 | 0.384 | 0.488 | 0.448 | 0.053 | -0.168 | -0.144 |
| DSS012 | 0.625 | 0.778 | 0.654 | 0.619 | 0.557 | 0.539 | 0.573 | 0.640 | 0.701 | 0.719 | 0.565 | 0.724 | 0.566 | 0.509 | 0.615 | 0.496 | -0.022 | -0.231 | -0.270 |
| DSS013 | 0.504 | 0.840 | 0.571 | 0.531 | 0.489 | 0.515 | 0.487 | 0.526 | 0.642 | 0.667 | 0.527 | 0.668 | 0.472 | 0.448 | 0.521 | 0.529 | -0.001 | -0.222 | -0.244 |
| DSS014 | 0.511 | 0.837 | 0.560 | 0.529 | 0.467 | 0.447 | 0.442 | 0.504 | 0.646 | 0.636 | 0.474 | 0.657 | 0.471 | 0.417 | 0.518 | 0.496 | 0.011 | -0.226 | -0.250 |
| DSS015 | 0.479 | 0.804 | 0.498 | 0.542 | 0.429 | 0.508 | 0.444 | 0.486 | 0.609 | 0.628 | 0.481 | 0.652 | 0.445 | 0.416 | 0.508 | 0.492 | 0.002 | -0.183 | -0.225 |
| DSS016 | 0.566 | 0.837 | 0.608 | 0.581 | 0.547 | 0.492 | 0.526 | 0.574 | 0.689 | 0.668 | 0.526 | 0.674 | 0.521 | 0.481 | 0.557 | 0.551 | -0.044 | -0.232 | -0.272 |
| DSS017 | 0.513 | 0.754 | 0.514 | 0.509 | 0.470 | 0.461 | 0.432 | 0.519 | 0.585 | 0.591 | 0.514 | 0.619 | 0.431 | 0.393 | 0.492 | 0.445 | 0.032 | -0.186 | -0.143 |
| DSS018 | 0.590 | 0.811 | 0.621 | 0.607 | 0.545 | 0.516 | 0.529 | 0.586 | 0.686 | 0.690 | 0.548 | 0.671 | 0.539 | 0.493 | 0.577 | 0.503 | -0.016 | -0.217 | -0.259 |
| DSS019 | 0.522 | 0.464 | 0.645 | 0.537 | 0.411 | 0.524 | 0.522 | 0.495 | 0.514 | 0.546 | 0.479 | 0.546 | 0.483 | 0.447 | 0.506 | 0.351 | -0.018 | -0.124 | -0.283 |
| DSS020 | 0.525 | 0.531 | 0.637 | 0.494 | 0.509 | 0.448 | 0.486 | 0.561 | 0.589 | 0.594 | 0.482 | 0.565 | 0.414 | 0.423 | 0.509 | 0.399 | 0.036 | -0.151 | -0.122 |
| DSS021 | 0.660 | 0.590 | 0.806 | 0.651 | 0.660 | 0.503 | 0.608 | 0.707 | 0.681 | 0.668 | 0.564 | 0.642 | 0.606 | 0.518 | 0.619 | 0.423 | -0.051 | -0.236 | -0.326 |
| DSS022 | -0.141 | -0.197 | -0.104 | -0.088 | -0.134 | -0.192 | -0.146 | -0.202 | -0.222 | -0.226 | -0.206 | -0.222 | 0.017 | -0.101 | -0.183 | -0.098 | -0.280 | -0.079 | -0.205 |
| DSS023 | 0.583 | 0.617 | 0.752 | 0.568 | 0.598 | 0.453 | 0.527 | 0.638 | 0.653 | 0.631 | 0.561 | 0.600 | 0.509 | 0.489 | 0.608 | 0.471 | 0.018 | -0.208 | -0.220 |
| DSS024 | 0.635 | 0.584 | 0.812 | 0.618 | 0.618 | 0.481 | 0.592 | 0.695 | 0.688 | 0.682 | 0.590 | 0.633 | 0.561 | 0.510 | 0.625 | 0.458 | -0.002 | -0.213 | -0.270 |
| DSS025 | 0.032 | -0.048 | 0.151 | 0.135 | 0.005 | -0.047 | 0.050 | -0.025 | -0.042 | -0.029 | -0.039 | -0.076 | 0.227 | 0.081 | 0.004 | 0.070 | -0.378 | -0.246 | -0.358 |
| DSS026 | 0.605 | 0.517 | 0.785 | 0.581 | 0.554 | 0.458 | 0.589 | 0.638 | 0.611 | 0.618 | 0.534 | 0.576 | 0.576 | 0.507 | 0.614 | 0.410 | -0.055 | -0.211 | -0.283 |
| DSS027 | 0.435 | 0.418 | 0.545 | 0.339 | 0.408 | 0.371 | 0.442 | 0.453 | 0.523 | 0.473 | 0.388 | 0.471 | 0.289 | 0.353 | 0.450 | 0.297 | 0.069 | -0.087 | -0.073 |
| DSS028 | 0.697 | 0.587 | 0.655 | 0.727 | 0.585 | 0.493 | 0.564 | 0.622 | 0.662 | 0.670 | 0.565 | 0.628 | 0.573 | 0.517 | 0.629 | 0.390 | 0.060 | -0.122 | -0.250 |
| DSS029 | 0.013 | -0.045 | 0.042 | 0.174 | 0.029 | -0.122 | 0.009 | -0.017 | -0.054 | -0.061 | -0.079 | -0.077 | 0.188 | 0.058 | -0.028 | 0.022 | -0.310 | -0.234 | -0.292 |
| DSS030 | 0.563 | 0.446 | 0.503 | 0.634 | 0.418 | 0.472 | 0.480 | 0.441 | 0.505 | 0.536 | 0.511 | 0.535 | 0.428 | 0.411 | 0.514 | 0.346 | 0.049 | -0.084 | -0.202 |
| DSS031 | 0.673 | 0.575 | 0.624 | 0.727 | 0.611 | 0.524 | 0.541 | 0.631 | 0.631 | 0.644 | 0.626 | 0.629 | 0.519 | 0.529 | 0.620 | 0.400 | 0.034 | -0.144 | -0.236 |
| DSS032 | 0.537 | 0.486 | 0.505 | 0.622 | 0.495 | 0.464 | 0.466 | 0.522 | 0.522 | 0.551 | 0.544 | 0.511 | 0.461 | 0.432 | 0.512 | 0.364 | 0.007 | -0.136 | -0.173 |
| DSS033 | -0.040 | -0.023 | -0.021 | 0.156 | -0.071 | -0.062 | -0.032 | -0.080 | -0.122 | -0.056 | -0.107 | -0.084 | 0.148 | 0.074 | -0.031 | 0.034 | -0.297 | -0.219 | -0.334 |
| DSS034 | 0.680 | 0.510 | 0.558 | 0.764 | 0.487 | 0.448 | 0.560 | 0.517 | 0.562 | 0.593 | 0.497 | 0.546 | 0.574 | 0.513 | 0.596 | 0.388 | -0.059 | -0.143 | -0.299 |
| DSS035 | 0.606 | 0.555 | 0.581 | 0.765 | 0.464 | 0.535 | 0.570 | 0.556 | 0.563 | 0.625 | 0.537 | 0.586 | 0.579 | 0.547 | 0.622 | 0.452 | -0.091 | -0.231 | -0.347 |
| DSS036 | 0.681 | 0.515 | 0.592 | 0.763 | 0.553 | 0.404 | 0.511 | 0.598 | 0.579 | 0.592 | 0.513 | 0.551 | 0.639 | 0.504 | 0.568 | 0.410 | -0.026 | -0.203 | -0.313 |
| DSS037 | 0.603 | 0.535 | 0.614 | 0.562 | 0.851 | 0.416 | 0.512 | 0.671 | 0.566 | 0.578 | 0.538 | 0.535 | 0.509 | 0.461 | 0.550 | 0.389 | 0.015 | -0.230 | -0.247 |
| DSS038 | 0.559 | 0.470 | 0.597 | 0.537 | 0.826 | 0.416 | 0.503 | 0.645 | 0.532 | 0.546 | 0.536 | 0.490 | 0.467 | 0.447 | 0.530 | 0.331 | 0.029 | -0.164 | -0.189 |
| DSS039 | 0.565 | 0.491 | 0.558 | 0.525 | 0.753 | 0.414 | 0.475 | 0.587 | 0.532 | 0.542 | 0.516 | 0.515 | 0.437 | 0.405 | 0.489 | 0.334 | 0.070 | -0.181 | -0.200 |
| DSS040 | 0.558 | 0.502 | 0.604 | 0.536 | 0.854 | 0.412 | 0.507 | 0.648 | 0.536 | 0.554 | 0.531 | 0.501 | 0.479 | 0.446 | 0.532 | 0.352 | 0.031 | -0.236 | -0.255 |
| DSS041 | 0.581 | 0.518 | 0.601 | 0.542 | 0.810 | 0.436 | 0.526 | 0.624 | 0.568 | 0.586 | 0.526 | 0.548 | 0.487 | 0.479 | 0.557 | 0.378 | 0.037 | -0.153 | -0.179 |
| DSS042 | 0.597 | 0.524 | 0.627 | 0.593 | 0.840 | 0.399 | 0.534 | 0.657 | 0.564 | 0.573 | 0.541 | 0.532 | 0.512 | 0.481 | 0.547 | 0.391 | -0.014 | -0.221 | -0.263 |
| DSS043 | 0.657 | 0.573 | 0.671 | 0.635 | 0.744 | 0.436 | 0.556 | 0.691 | 0.645 | 0.666 | 0.558 | 0.597 | 0.578 | 0.515 | 0.614 | 0.419 | -0.000 | -0.220 | -0.269 |
| DSS044 | 0.527 | 0.453 | 0.559 | 0.504 | 0.837 | 0.379 | 0.465 | 0.607 | 0.499 | 0.511 | 0.509 | 0.456 | 0.447 | 0.416 | 0.504 | 0.309 | 0.019 | -0.179 | -0.172 |
| DSS045 | 0.594 | 0.495 | 0.590 | 0.554 | 0.860 | 0.397 | 0.510 | 0.642 | 0.548 | 0.553 | 0.529 | 0.508 | 0.489 | 0.449 | 0.536 | 0.351 | 0.039 | -0.186 | -0.215 |
| DSS046 | 0.521 | 0.537 | 0.496 | 0.548 | 0.424 | 0.769 | 0.486 | 0.482 | 0.522 | 0.579 | 0.567 | 0.583 | 0.454 | 0.444 | 0.511 | 0.381 | -0.032 | -0.173 | -0.227 |
| DSS047 | 0.451 | 0.517 | 0.530 | 0.474 | 0.416 | 0.797 | 0.491 | 0.473 | 0.520 | 0.549 | 0.504 | 0.569 | 0.418 | 0.416 | 0.480 | 0.399 | -0.037 | -0.173 | -0.185 |
| DSS048 | 0.449 | 0.475 | 0.532 | 0.460 | 0.424 | 0.735 | 0.517 | 0.492 | 0.500 | 0.559 | 0.492 | 0.544 | 0.411 | 0.440 | 0.491 | 0.378 | -0.053 | -0.160 | -0.166 |
| DSS049 | 0.435 | 0.468 | 0.424 | 0.453 | 0.343 | 0.754 | 0.408 | 0.420 | 0.454 | 0.500 | 0.536 | 0.518 | 0.368 | 0.407 | 0.453 | 0.323 | 0.031 | -0.102 | -0.154 |
| DSS050 | 0.427 | 0.426 | 0.441 | 0.422 | 0.394 | 0.718 | 0.382 | 0.420 | 0.432 | 0.490 | 0.618 | 0.491 | 0.350 | 0.373 | 0.453 | 0.303 | 0.051 | -0.085 | -0.136 |
| DSS051 | 0.429 | 0.473 | 0.454 | 0.464 | 0.341 | 0.756 | 0.422 | 0.396 | 0.446 | 0.503 | 0.539 | 0.538 | 0.354 | 0.418 | 0.488 | 0.313 | 0.024 | -0.077 | -0.141 |
| DSS052 | 0.483 | 0.519 | 0.499 | 0.528 | 0.418 | 0.778 | 0.506 | 0.501 | 0.514 | 0.568 | 0.558 | 0.569 | 0.439 | 0.453 | 0.518 | 0.424 | -0.053 | -0.172 | -0.220 |
| DSS053 | 0.330 | 0.337 | 0.351 | 0.348 | 0.272 | 0.716 | 0.335 | 0.315 | 0.315 | 0.380 | 0.504 | 0.397 | 0.301 | 0.348 | 0.397 | 0.272 | 0.004 | -0.037 | -0.102 |
| DSS054 | 0.314 | 0.329 | 0.359 | 0.354 | 0.298 | 0.719 | 0.347 | 0.312 | 0.319 | 0.375 | 0.428 | 0.391 | 0.282 | 0.321 | 0.361 | 0.271 | -0.011 | -0.079 | -0.144 |
| DSS055 | 0.519 | 0.482 | 0.486 | 0.485 | 0.491 | 0.314 | 0.488 | 0.556 | 0.514 | 0.507 | 0.482 | 0.492 | 0.426 | 0.387 | 0.468 | 0.314 | 0.138 | -0.075 | -0.145 |
| DSS056 | -0.219 | -0.237 | -0.168 | -0.183 | -0.197 | -0.162 | 0.011 | -0.222 | -0.250 | -0.251 | -0.262 | -0.264 | -0.079 | -0.105 | -0.216 | -0.025 | -0.411 | -0.177 | -0.060 |
| DSS057 | 0.489 | 0.426 | 0.490 | 0.489 | 0.396 | 0.462 | 0.662 | 0.412 | 0.457 | 0.514 | 0.442 | 0.503 | 0.416 | 0.444 | 0.503 | 0.337 | 0.001 | -0.058 | -0.228 |
| DSS058 | 0.689 | 0.577 | 0.664 | 0.638 | 0.573 | 0.500 | 0.765 | 0.625 | 0.681 | 0.682 | 0.573 | 0.640 | 0.593 | 0.573 | 0.646 | 0.440 | -0.040 | -0.157 | -0.281 |
| DSS059 | 0.483 | 0.405 | 0.482 | 0.465 | 0.445 | 0.379 | 0.697 | 0.479 | 0.433 | 0.459 | 0.395 | 0.442 | 0.458 | 0.397 | 0.446 | 0.332 | -0.090 | -0.164 | -0.231 |
| DSS060 | 0.628 | 0.541 | 0.660 | 0.626 | 0.527 | 0.499 | 0.791 | 0.597 | 0.609 | 0.637 | 0.534 | 0.594 | 0.580 | 0.553 | 0.627 | 0.452 | -0.118 | -0.190 | -0.317 |
| DSS061 | 0.482 | 0.456 | 0.484 | 0.456 | 0.393 | 0.430 | 0.609 | 0.505 | 0.498 | 0.540 | 0.478 | 0.509 | 0.400 | 0.459 | 0.549 | 0.351 | 0.010 | -0.090 | -0.179 |
| DSS062 | 0.198 | 0.100 | 0.206 | 0.187 | 0.204 | 0.229 | 0.511 | 0.190 | 0.140 | 0.173 | 0.133 | 0.164 | 0.237 | 0.203 | 0.188 | 0.223 | -0.192 | -0.124 | -0.142 |
| DSS063 | -0.144 | -0.217 | -0.070 | -0.122 | -0.125 | -0.065 | 0.190 | -0.160 | -0.178 | -0.161 | -0.218 | -0.173 | 0.004 | -0.057 | -0.152 | 0.041 | -0.411 | -0.170 | -0.136 |
| DSS064 | 0.646 | 0.653 | 0.673 | 0.598 | 0.571 | 0.485 | 0.573 | 0.664 | 0.832 | 0.701 | 0.530 | 0.669 | 0.560 | 0.505 | 0.599 | 0.503 | -0.050 | -0.244 | -0.284 |
| DSS065 | 0.590 | 0.634 | 0.637 | 0.568 | 0.507 | 0.480 | 0.532 | 0.620 | 0.790 | 0.682 | 0.538 | 0.665 | 0.512 | 0.489 | 0.584 | 0.482 | 0.023 | -0.192 | -0.204 |
| DSS066 | 0.581 | 0.509 | 0.541 | 0.536 | 0.459 | 0.419 | 0.468 | 0.514 | 0.687 | 0.569 | 0.441 | 0.548 | 0.453 | 0.400 | 0.483 | 0.365 | 0.017 | -0.141 | -0.219 |
| DSS067 | 0.421 | 0.550 | 0.502 | 0.418 | 0.396 | 0.442 | 0.426 | 0.497 | 0.654 | 0.570 | 0.451 | 0.587 | 0.383 | 0.376 | 0.472 | 0.356 | 0.041 | -0.140 | -0.118 |
| DSS068 | 0.648 | 0.663 | 0.688 | 0.607 | 0.589 | 0.470 | 0.553 | 0.645 | 0.791 | 0.692 | 0.552 | 0.660 | 0.542 | 0.513 | 0.606 | 0.510 | -0.027 | -0.232 | -0.244 |
| DSS069 | 0.386 | 0.512 | 0.453 | 0.385 | 0.362 | 0.458 | 0.383 | 0.448 | 0.592 | 0.541 | 0.446 | 0.555 | 0.341 | 0.343 | 0.412 | 0.288 | 0.138 | -0.059 | -0.071 |
| DSS070 | 0.690 | 0.644 | 0.662 | 0.652 | 0.527 | 0.496 | 0.588 | 0.599 | 0.791 | 0.714 | 0.561 | 0.673 | 0.612 | 0.548 | 0.613 | 0.491 | -0.018 | -0.196 | -0.260 |
| DSS071 | 0.255 | 0.370 | 0.324 | 0.237 | 0.254 | 0.330 | 0.286 | 0.345 | 0.440 | 0.391 | 0.302 | 0.399 | 0.142 | 0.203 | 0.314 | 0.192 | 0.238 | 0.037 | 0.097 |
| DSS072 | 0.643 | 0.628 | 0.653 | 0.606 | 0.579 | 0.413 | 0.531 | 0.644 | 0.776 | 0.668 | 0.521 | 0.611 | 0.553 | 0.487 | 0.581 | 0.458 | -0.041 | -0.246 | -0.286 |
| DSS073 | 0.476 | 0.419 | 0.472 | 0.443 | 0.597 | 0.311 | 0.406 | 0.641 | 0.460 | 0.461 | 0.444 | 0.437 | 0.370 | 0.369 | 0.427 | 0.309 | 0.058 | -0.134 | -0.092 |
| DSS074 | 0.609 | 0.553 | 0.659 | 0.599 | 0.625 | 0.467 | 0.550 | 0.821 | 0.653 | 0.620 | 0.552 | 0.592 | 0.561 | 0.514 | 0.609 | 0.465 | -0.058 | -0.292 | -0.295 |
| DSS075 | 0.583 | 0.539 | 0.679 | 0.569 | 0.572 | 0.504 | 0.579 | 0.758 | 0.645 | 0.626 | 0.549 | 0.606 | 0.503 | 0.500 | 0.580 | 0.462 | -0.006 | -0.229 | -0.233 |
| DSS076 | 0.644 | 0.569 | 0.669 | 0.614 | 0.599 | 0.496 | 0.580 | 0.736 | 0.650 | 0.661 | 0.583 | 0.621 | 0.547 | 0.521 | 0.613 | 0.385 | 0.001 | -0.139 | -0.234 |
| DSS077 | 0.652 | 0.583 | 0.675 | 0.603 | 0.617 | 0.466 | 0.547 | 0.796 | 0.651 | 0.647 | 0.565 | 0.624 | 0.575 | 0.484 | 0.599 | 0.398 | -0.010 | -0.219 | -0.291 |
| DSS078 | 0.401 | 0.307 | 0.433 | 0.346 | 0.450 | 0.277 | 0.300 | 0.514 | 0.416 | 0.393 | 0.389 | 0.366 | 0.297 | 0.284 | 0.377 | 0.123 | 0.176 | 0.026 | -0.019 |
| DSS079 | 0.434 | 0.403 | 0.489 | 0.404 | 0.521 | 0.330 | 0.424 | 0.699 | 0.456 | 0.486 | 0.416 | 0.451 | 0.382 | 0.352 | 0.447 | 0.334 | 0.035 | -0.193 | -0.115 |
| DSS080 | 0.591 | 0.526 | 0.642 | 0.597 | 0.602 | 0.434 | 0.564 | 0.786 | 0.609 | 0.598 | 0.492 | 0.571 | 0.571 | 0.483 | 0.565 | 0.447 | -0.079 | -0.301 | -0.355 |
| DSS081 | 0.587 | 0.530 | 0.604 | 0.551 | 0.644 | 0.455 | 0.516 | 0.801 | 0.592 | 0.601 | 0.586 | 0.556 | 0.489 | 0.473 | 0.569 | 0.433 | 0.032 | -0.240 | -0.199 |
| DSS082 | 0.613 | 0.631 | 0.632 | 0.618 | 0.556 | 0.505 | 0.562 | 0.625 | 0.671 | 0.782 | 0.564 | 0.659 | 0.574 | 0.488 | 0.592 | 0.479 | -0.043 | -0.248 | -0.286 |
| DSS083 | 0.599 | 0.676 | 0.625 | 0.604 | 0.553 | 0.535 | 0.567 | 0.616 | 0.692 | 0.783 | 0.572 | 0.678 | 0.602 | 0.520 | 0.595 | 0.513 | -0.078 | -0.252 | -0.310 |
| DSS084 | 0.691 | 0.607 | 0.666 | 0.675 | 0.597 | 0.502 | 0.574 | 0.632 | 0.672 | 0.755 | 0.599 | 0.648 | 0.594 | 0.551 | 0.678 | 0.487 | 0.003 | -0.165 | -0.307 |
| DSS085 | 0.616 | 0.539 | 0.595 | 0.593 | 0.470 | 0.474 | 0.556 | 0.560 | 0.599 | 0.694 | 0.562 | 0.591 | 0.551 | 0.598 | 0.692 | 0.456 | -0.018 | -0.177 | -0.273 |
| DSS086 | 0.480 | 0.570 | 0.494 | 0.475 | 0.379 | 0.482 | 0.436 | 0.455 | 0.573 | 0.695 | 0.465 | 0.591 | 0.438 | 0.416 | 0.490 | 0.403 | -0.006 | -0.162 | -0.186 |
| DSS087 | 0.332 | 0.389 | 0.362 | 0.389 | 0.274 | 0.451 | 0.389 | 0.344 | 0.381 | 0.482 | 0.376 | 0.421 | 0.314 | 0.302 | 0.372 | 0.320 | 0.040 | -0.078 | -0.147 |
| DSS088 | 0.633 | 0.604 | 0.641 | 0.594 | 0.535 | 0.525 | 0.582 | 0.606 | 0.667 | 0.760 | 0.563 | 0.633 | 0.566 | 0.572 | 0.648 | 0.466 | -0.007 | -0.173 | -0.257 |
| DSS089 | 0.465 | 0.563 | 0.435 | 0.443 | 0.440 | 0.407 | 0.390 | 0.472 | 0.491 | 0.609 | 0.440 | 0.574 | 0.372 | 0.350 | 0.414 | 0.423 | 0.052 | -0.186 | -0.135 |
| DSS090 | 0.593 | 0.567 | 0.603 | 0.579 | 0.539 | 0.506 | 0.535 | 0.604 | 0.620 | 0.709 | 0.627 | 0.613 | 0.547 | 0.534 | 0.640 | 0.417 | 0.047 | -0.158 | -0.231 |
| DSS091 | 0.509 | 0.549 | 0.551 | 0.496 | 0.500 | 0.407 | 0.453 | 0.526 | 0.585 | 0.681 | 0.458 | 0.581 | 0.460 | 0.417 | 0.493 | 0.391 | 0.056 | -0.169 | -0.193 |
| DSS092 | 0.646 | 0.612 | 0.648 | 0.631 | 0.545 | 0.498 | 0.571 | 0.596 | 0.700 | 0.781 | 0.539 | 0.653 | 0.610 | 0.550 | 0.612 | 0.477 | -0.037 | -0.211 | -0.315 |
| DSS093 | 0.485 | 0.563 | 0.548 | 0.489 | 0.424 | 0.470 | 0.493 | 0.485 | 0.614 | 0.667 | 0.461 | 0.599 | 0.411 | 0.398 | 0.491 | 0.380 | 0.066 | -0.113 | -0.144 |
| DSS094 | 0.479 | 0.450 | 0.469 | 0.485 | 0.458 | 0.560 | 0.399 | 0.486 | 0.456 | 0.505 | 0.768 | 0.473 | 0.385 | 0.423 | 0.510 | 0.344 | 0.049 | -0.123 | -0.153 |
| DSS095 | 0.526 | 0.458 | 0.507 | 0.499 | 0.500 | 0.555 | 0.450 | 0.527 | 0.488 | 0.544 | 0.808 | 0.501 | 0.417 | 0.449 | 0.538 | 0.332 | 0.029 | -0.119 | -0.191 |
| DSS096 | 0.581 | 0.569 | 0.589 | 0.582 | 0.483 | 0.514 | 0.560 | 0.554 | 0.625 | 0.658 | 0.690 | 0.617 | 0.526 | 0.525 | 0.626 | 0.372 | 0.032 | -0.120 | -0.211 |
| DSS097 | 0.656 | 0.560 | 0.611 | 0.648 | 0.626 | 0.479 | 0.553 | 0.634 | 0.616 | 0.631 | 0.710 | 0.591 | 0.535 | 0.518 | 0.619 | 0.385 | 0.081 | -0.147 | -0.243 |
| DSS098 | 0.529 | 0.501 | 0.535 | 0.508 | 0.483 | 0.412 | 0.400 | 0.532 | 0.541 | 0.571 | 0.673 | 0.532 | 0.433 | 0.427 | 0.538 | 0.312 | 0.091 | -0.071 | -0.138 |
| DSS099 | 0.498 | 0.460 | 0.480 | 0.495 | 0.484 | 0.561 | 0.410 | 0.507 | 0.476 | 0.525 | 0.719 | 0.498 | 0.374 | 0.365 | 0.480 | 0.302 | 0.089 | -0.094 | -0.130 |
| DSS100 | 0.422 | 0.386 | 0.419 | 0.414 | 0.369 | 0.491 | 0.379 | 0.403 | 0.368 | 0.435 | 0.732 | 0.414 | 0.346 | 0.415 | 0.453 | 0.324 | -0.032 | -0.109 | -0.187 |
| DSS101 | 0.346 | 0.309 | 0.363 | 0.343 | 0.296 | 0.461 | 0.359 | 0.324 | 0.331 | 0.387 | 0.650 | 0.361 | 0.314 | 0.368 | 0.403 | 0.292 | -0.025 | -0.070 | -0.188 |
| DSS102 | 0.389 | 0.388 | 0.405 | 0.398 | 0.396 | 0.453 | 0.334 | 0.402 | 0.370 | 0.419 | 0.628 | 0.399 | 0.335 | 0.373 | 0.408 | 0.266 | 0.031 | -0.103 | -0.182 |
| DSS103 | 0.605 | 0.671 | 0.632 | 0.602 | 0.530 | 0.502 | 0.542 | 0.602 | 0.702 | 0.702 | 0.569 | 0.773 | 0.541 | 0.526 | 0.612 | 0.519 | -0.013 | -0.231 | -0.245 |
| DSS104 | 0.548 | 0.502 | 0.542 | 0.496 | 0.481 | 0.425 | 0.480 | 0.582 | 0.571 | 0.570 | 0.486 | 0.648 | 0.453 | 0.449 | 0.511 | 0.349 | 0.028 | -0.116 | -0.215 |
| DSS105 | 0.604 | 0.545 | 0.598 | 0.563 | 0.528 | 0.448 | 0.526 | 0.604 | 0.620 | 0.633 | 0.541 | 0.688 | 0.509 | 0.466 | 0.585 | 0.378 | 0.034 | -0.127 | -0.217 |
| DSS106 | 0.431 | 0.615 | 0.486 | 0.471 | 0.338 | 0.521 | 0.437 | 0.439 | 0.552 | 0.558 | 0.430 | 0.698 | 0.420 | 0.366 | 0.457 | 0.416 | -0.007 | -0.161 | -0.202 |
| DSS107 | 0.477 | 0.529 | 0.467 | 0.472 | 0.407 | 0.422 | 0.396 | 0.477 | 0.519 | 0.543 | 0.433 | 0.650 | 0.404 | 0.367 | 0.466 | 0.370 | 0.031 | -0.144 | -0.174 |
| DSS108 | 0.452 | 0.554 | 0.491 | 0.491 | 0.320 | 0.575 | 0.471 | 0.400 | 0.516 | 0.571 | 0.434 | 0.689 | 0.424 | 0.426 | 0.484 | 0.443 | -0.029 | -0.179 | -0.220 |
| DSS109 | 0.571 | 0.672 | 0.578 | 0.558 | 0.540 | 0.512 | 0.508 | 0.561 | 0.636 | 0.665 | 0.521 | 0.762 | 0.491 | 0.489 | 0.553 | 0.499 | 0.001 | -0.201 | -0.229 |
| DSS110 | -0.377 | -0.427 | -0.393 | -0.349 | -0.341 | -0.388 | -0.399 | -0.402 | -0.422 | -0.434 | -0.398 | -0.555 | -0.292 | -0.327 | -0.379 | -0.316 | -0.018 | 0.090 | 0.061 |
| DSS111 | -0.064 | -0.170 | -0.097 | -0.018 | -0.078 | -0.198 | -0.099 | -0.119 | -0.151 | -0.152 | -0.161 | -0.177 | 0.052 | -0.014 | -0.109 | -0.077 | -0.234 | -0.083 | -0.210 |
| DSS112 | 0.688 | 0.572 | 0.641 | 0.664 | 0.577 | 0.479 | 0.568 | 0.634 | 0.630 | 0.665 | 0.568 | 0.620 | 0.771 | 0.596 | 0.650 | 0.484 | -0.090 | -0.230 | -0.338 |
| DSS113 | 0.671 | 0.513 | 0.617 | 0.653 | 0.536 | 0.422 | 0.545 | 0.606 | 0.573 | 0.600 | 0.511 | 0.575 | 0.752 | 0.540 | 0.592 | 0.415 | -0.073 | -0.217 | -0.361 |
| DSS114 | -0.085 | -0.173 | -0.068 | -0.018 | -0.095 | -0.145 | -0.107 | -0.128 | -0.164 | -0.147 | -0.142 | -0.183 | 0.173 | -0.032 | -0.128 | -0.019 | -0.370 | -0.216 | -0.258 |
| DSS115 | 0.634 | 0.492 | 0.529 | 0.603 | 0.438 | 0.457 | 0.532 | 0.525 | 0.553 | 0.559 | 0.481 | 0.567 | 0.629 | 0.480 | 0.568 | 0.393 | -0.008 | -0.113 | -0.278 |
| DSS116 | 0.648 | 0.549 | 0.657 | 0.575 | 0.535 | 0.429 | 0.584 | 0.595 | 0.656 | 0.669 | 0.502 | 0.602 | 0.698 | 0.545 | 0.631 | 0.437 | -0.039 | -0.196 | -0.259 |
| DSS117 | 0.618 | 0.472 | 0.544 | 0.610 | 0.492 | 0.424 | 0.533 | 0.566 | 0.520 | 0.567 | 0.489 | 0.549 | 0.733 | 0.532 | 0.559 | 0.400 | -0.032 | -0.188 | -0.349 |
| DSS118 | 0.575 | 0.508 | 0.577 | 0.546 | 0.480 | 0.439 | 0.563 | 0.555 | 0.584 | 0.636 | 0.491 | 0.543 | 0.666 | 0.514 | 0.605 | 0.430 | 0.007 | -0.183 | -0.282 |
| DSS119 | -0.012 | -0.041 | -0.034 | 0.033 | -0.085 | -0.034 | 0.014 | -0.126 | -0.078 | -0.022 | -0.066 | -0.099 | 0.262 | 0.114 | -0.006 | 0.047 | -0.398 | -0.214 | -0.295 |
| DSS120 | 0.012 | -0.014 | 0.028 | 0.098 | -0.002 | -0.027 | 0.039 | -0.042 | -0.059 | -0.027 | -0.054 | -0.064 | 0.349 | 0.136 | 0.031 | 0.133 | -0.425 | -0.291 | -0.351 |
| DSS121 | 0.675 | 0.584 | 0.647 | 0.652 | 0.566 | 0.509 | 0.595 | 0.636 | 0.652 | 0.680 | 0.591 | 0.625 | 0.627 | 0.744 | 0.699 | 0.483 | -0.081 | -0.230 | -0.263 |
| DSS122 | 0.616 | 0.492 | 0.584 | 0.587 | 0.503 | 0.480 | 0.566 | 0.554 | 0.582 | 0.611 | 0.562 | 0.563 | 0.591 | 0.779 | 0.679 | 0.438 | -0.089 | -0.183 | -0.264 |
| DSS123 | 0.521 | 0.396 | 0.477 | 0.504 | 0.431 | 0.427 | 0.493 | 0.469 | 0.458 | 0.511 | 0.498 | 0.469 | 0.515 | 0.752 | 0.583 | 0.375 | -0.103 | -0.136 | -0.267 |
| DSS124 | 0.592 | 0.498 | 0.589 | 0.579 | 0.530 | 0.459 | 0.550 | 0.580 | 0.569 | 0.620 | 0.541 | 0.561 | 0.564 | 0.765 | 0.697 | 0.446 | -0.068 | -0.177 | -0.288 |
| DSS125 | 0.336 | 0.307 | 0.351 | 0.384 | 0.228 | 0.441 | 0.404 | 0.318 | 0.322 | 0.404 | 0.347 | 0.405 | 0.306 | 0.519 | 0.456 | 0.322 | -0.016 | -0.060 | -0.212 |
| DSS126 | 0.495 | 0.414 | 0.470 | 0.463 | 0.492 | 0.343 | 0.471 | 0.510 | 0.473 | 0.502 | 0.455 | 0.458 | 0.453 | 0.634 | 0.518 | 0.330 | -0.030 | -0.150 | -0.192 |
| DSS127 | 0.470 | 0.421 | 0.432 | 0.484 | 0.443 | 0.393 | 0.373 | 0.464 | 0.455 | 0.464 | 0.460 | 0.441 | 0.409 | 0.567 | 0.531 | 0.335 | 0.062 | -0.093 | -0.127 |
| DSS128 | 0.048 | 0.041 | 0.076 | 0.126 | -0.016 | 0.059 | 0.113 | -0.005 | 0.015 | 0.066 | 0.027 | 0.053 | 0.229 | 0.470 | 0.146 | 0.172 | -0.433 | -0.258 | -0.324 |
| DSS129 | 0.090 | 0.067 | 0.083 | 0.135 | 0.051 | 0.057 | 0.134 | 0.028 | 0.050 | 0.077 | 0.066 | 0.066 | 0.267 | 0.503 | 0.140 | 0.190 | -0.433 | -0.244 | -0.287 |
| DSS130 | 0.645 | 0.562 | 0.635 | 0.629 | 0.525 | 0.500 | 0.624 | 0.591 | 0.629 | 0.668 | 0.561 | 0.622 | 0.605 | 0.623 | 0.795 | 0.462 | -0.068 | -0.184 | -0.305 |
| DSS131 | 0.614 | 0.510 | 0.596 | 0.611 | 0.517 | 0.479 | 0.561 | 0.557 | 0.566 | 0.629 | 0.579 | 0.575 | 0.575 | 0.661 | 0.797 | 0.436 | -0.019 | -0.163 | -0.320 |
| DSS132 | 0.626 | 0.545 | 0.597 | 0.612 | 0.551 | 0.420 | 0.518 | 0.581 | 0.574 | 0.626 | 0.553 | 0.570 | 0.587 | 0.627 | 0.773 | 0.452 | -0.004 | -0.190 | -0.324 |
| DSS133 | 0.699 | 0.580 | 0.679 | 0.680 | 0.609 | 0.479 | 0.619 | 0.658 | 0.652 | 0.699 | 0.591 | 0.636 | 0.641 | 0.643 | 0.810 | 0.461 | -0.036 | -0.228 | -0.349 |
| DSS134 | 0.518 | 0.459 | 0.500 | 0.495 | 0.427 | 0.492 | 0.478 | 0.482 | 0.478 | 0.544 | 0.550 | 0.504 | 0.439 | 0.535 | 0.733 | 0.414 | -0.002 | -0.157 | -0.236 |
| DSS135 | 0.349 | 0.343 | 0.360 | 0.353 | 0.260 | 0.441 | 0.330 | 0.323 | 0.368 | 0.418 | 0.407 | 0.419 | 0.291 | 0.331 | 0.506 | 0.331 | 0.051 | -0.067 | -0.111 |
| DSS136 | 0.590 | 0.533 | 0.586 | 0.585 | 0.499 | 0.475 | 0.570 | 0.583 | 0.581 | 0.626 | 0.537 | 0.566 | 0.593 | 0.604 | 0.759 | 0.494 | -0.072 | -0.226 | -0.319 |
| DSS137 | 0.618 | 0.471 | 0.595 | 0.607 | 0.525 | 0.443 | 0.520 | 0.575 | 0.550 | 0.574 | 0.539 | 0.531 | 0.569 | 0.542 | 0.724 | 0.371 | -0.001 | -0.152 | -0.249 |
| DSS138 | 0.530 | 0.457 | 0.517 | 0.506 | 0.443 | 0.468 | 0.489 | 0.493 | 0.491 | 0.541 | 0.529 | 0.515 | 0.459 | 0.522 | 0.751 | 0.388 | -0.002 | -0.160 | -0.241 |
| EMEX01 | 0.416 | 0.507 | 0.448 | 0.434 | 0.362 | 0.376 | 0.435 | 0.445 | 0.487 | 0.510 | 0.368 | 0.493 | 0.482 | 0.461 | 0.466 | 0.864 | -0.260 | -0.480 | -0.317 |
| EMEX02 | 0.445 | 0.529 | 0.474 | 0.437 | 0.354 | 0.400 | 0.462 | 0.428 | 0.503 | 0.521 | 0.387 | 0.519 | 0.470 | 0.468 | 0.478 | 0.826 | -0.220 | -0.343 | -0.278 |
| EMEX03 | 0.466 | 0.521 | 0.493 | 0.466 | 0.384 | 0.386 | 0.479 | 0.460 | 0.515 | 0.535 | 0.385 | 0.518 | 0.479 | 0.464 | 0.514 | 0.863 | -0.236 | -0.427 | -0.331 |
| EMEX04 | 0.443 | 0.521 | 0.468 | 0.463 | 0.409 | 0.410 | 0.451 | 0.481 | 0.486 | 0.526 | 0.411 | 0.520 | 0.463 | 0.457 | 0.490 | 0.848 | -0.221 | -0.469 | -0.290 |
| EMEX05 | 0.409 | 0.475 | 0.439 | 0.422 | 0.340 | 0.360 | 0.389 | 0.417 | 0.463 | 0.491 | 0.375 | 0.486 | 0.402 | 0.384 | 0.431 | 0.748 | -0.110 | -0.344 | -0.265 |
| INNO01 | -0.100 | -0.050 | -0.092 | -0.131 | -0.050 | -0.019 | -0.146 | -0.068 | -0.038 | -0.043 | -0.018 | -0.039 | -0.243 | -0.194 | -0.052 | -0.194 | 0.737 | 0.402 | 0.308 |
| INNO02 | -0.064 | -0.069 | -0.110 | -0.109 | -0.055 | -0.019 | -0.119 | -0.083 | -0.059 | -0.053 | 0.011 | -0.054 | -0.237 | -0.219 | -0.072 | -0.245 | 0.744 | 0.465 | 0.323 |
| INNO03 | 0.004 | 0.046 | -0.030 | -0.053 | 0.060 | -0.011 | -0.092 | 0.031 | 0.054 | 0.044 | 0.071 | 0.034 | -0.149 | -0.139 | 0.008 | -0.171 | 0.737 | 0.348 | 0.267 |
| INNO04 | 0.093 | 0.131 | 0.037 | 0.045 | 0.115 | 0.055 | -0.057 | 0.090 | 0.121 | 0.121 | 0.140 | 0.119 | -0.044 | -0.067 | 0.073 | -0.076 | 0.720 | 0.292 | 0.204 |
| INNO05 | -0.029 | -0.039 | -0.077 | -0.075 | 0.039 | -0.063 | -0.149 | 0.001 | -0.042 | -0.057 | 0.011 | -0.036 | -0.215 | -0.208 | -0.058 | -0.249 | 0.782 | 0.361 | 0.325 |
| JOSA01 | -0.195 | -0.222 | -0.255 | -0.250 | -0.193 | -0.145 | -0.199 | -0.256 | -0.210 | -0.233 | -0.131 | -0.202 | -0.303 | -0.247 | -0.213 | -0.423 | 0.406 | 0.872 | 0.393 |
| JOSA02 | -0.152 | -0.199 | -0.219 | -0.184 | -0.209 | -0.103 | -0.137 | -0.236 | -0.203 | -0.176 | -0.104 | -0.169 | -0.264 | -0.178 | -0.159 | -0.343 | 0.371 | 0.816 | 0.341 |
| JOSA03 | -0.193 | -0.257 | -0.266 | -0.250 | -0.224 | -0.180 | -0.236 | -0.264 | -0.233 | -0.242 | -0.156 | -0.238 | -0.331 | -0.282 | -0.230 | -0.505 | 0.509 | 0.888 | 0.445 |
| USSA01 | -0.393 | -0.277 | -0.380 | -0.423 | -0.287 | -0.220 | -0.348 | -0.321 | -0.296 | -0.332 | -0.241 | -0.290 | -0.512 | -0.379 | -0.365 | -0.344 | 0.361 | 0.453 | 0.920 |
| USSA02 | -0.340 | -0.256 | -0.340 | -0.396 | -0.249 | -0.190 | -0.320 | -0.270 | -0.257 | -0.310 | -0.241 | -0.271 | -0.477 | -0.356 | -0.335 | -0.322 | 0.350 | 0.411 | 0.923 |
| USSA03 | -0.362 | -0.269 | -0.360 | -0.414 | -0.250 | -0.214 | -0.339 | -0.295 | -0.282 | -0.325 | -0.243 | -0.289 | -0.498 | -0.370 | -0.367 | -0.325 | 0.349 | 0.413 | 0.908 |
| USSA04 | -0.296 | -0.224 | -0.295 | -0.325 | -0.196 | -0.193 | -0.279 | -0.232 | -0.205 | -0.252 | -0.202 | -0.218 | -0.414 | -0.315 | -0.288 | -0.294 | 0.331 | 0.379 | 0.856 |
| DSS000 = Items in the DSS; EMEX00 = Items of Emotional Exhaustion; INNO00 = Items of Innovation Climate; JOSA00 = Items of Job Satisfaction; USSA00 = Items of User Satisfaction  I = Complexity, II = Conflicts, III = Control, IV = Costs, V = Insecurity, VI = Invasion, VII = Involvement, VIII = Boredom, IX = Overload, X = Role Stress, XI = Safety, XII = Social Environment, XIII = Usefulness, XIV = Technical Support, XV = Unreliability  A = Emotional Exhaustion, B = Innovation Climate, C = Job Satisfaction, D = User Satisfaction | | | | | | | | | | | | | | | | | | | |

## Model Specification

Based on Coltman, Devinney, Midgley, and Venaik (2008), theoretical and empirical considerations are used to assess whether the 2^nd^ Order DSS construct should be modeled as reflective or formative. *Theoretical considerations* include (i) the nature of the construct (e.g., Is the 2^nd^ order construct a combination of the 1^st^ order constructs?), (2) the direction of causality (e.g., Does variation in the 2^nd^ order construct cause variation in the 1^st^ order constructs?), and (3) the characteristics of indicators (e.g., Are the 1^st^ order constructs interchangeable?). Existing scales that measure constructs comparable to digital stress have used both formative and reflective specifications. For example, the Computer Hassles scale by Hudiburg (1995), uses a list of potential stressors that form a hassles score indicative of stress perceptions caused by ICT (formative 1^st^ order construct). Regarding higher order stress constructs, the Technostress Creators scale by Ragu-Nathan, Tarafdar, Ragu-Nathan, and Tu (2008) uses a set of five reflective 1^st^ order constructs that are included in a reflective 2^nd^ order construct. A different conceptualization is used, for example, for the concept of role stress, which is commonly measured through three reflective 1^st^ order constructs (role conflict, role ambiguity, role overload) that then form a 2^nd^ order construct (e.g., Edwards, 2001). In the case of the proposed Digital Stressors scale, reflective and formative relationships between 1^st^ order constructs and a 2^nd^ order construct could be argued for based on these examples. Hence, the specification of the construct is mainly based on empirical considerations rather than theoretical considerations.

*Empirical considerations* include (i) item intercorrelations (e.g., same direction of relationships in both cases and highly positive intercorrelations for reflective constructs specifically), (ii) indicator relationships with construct antecedents and consequences (e.g., 1^st^ order constructs may not have the same sign and significance with construct consequences in the case of formative constructs) and (iii) measurement error and collinearity (e.g., vanishing tetrad test to check the significance of a formative specification). As basis for these empirical tests, 1^st^ order constructs were first transformed into construct indicators using the disjoint two-stage approach and in the case of a reflective specification Mode A was used for model estimation, while Mode B was used for the formative specification (Sarstedt, Hair, Cheah, Becker, & Ringle, 2019). The inter-construct correlations of the 1^st^ order constructs (see Table 8 in section 4.2) are all positive and comparatively high, ranging from .486 to .825 (inter-construct correlations of 1^st^ order constructs for the Technostress Creators scale, which is specified as reflective construct, range from .328 to .727; see Table 12). Regarding the relationships of the 1^st^ order constructs with the four selected criterion variables, a substantially varied picture emerges (see Table 13 for path coefficients and significance of paths). In some instances, none of the proposed relationships are significant (e.g., for Safety), while in some instances significant paths show a different sign than expected (e.g., Overload shows a positive relationship with User Satisfaction). Hence, while the highly positive inter-construct correlations might indicate a reflective specification to be appropriate, the varied relationships of 1^st^ order constructs with the selected criterion variables point to a formative specification. Therefore, a confirmatory tetrad analysis (CTA-PLS, Gudergan, Ringle, Wende, & Will, 2008) was conducted with the ten 1^st^ order constructs serving as indicators for a 2^nd^ order construct that is then connected to the four criterion variables. Of the 35 calculated tetrads, 18 were vanishing (i.e., the adjusted confidence interval covers 0) and therefore do not reject the null hypothesis of a reflective specification (see Table 14). As only a minority of 17 tetrads indicates a formative specification, the CTA as a whole therefore supports a reflective specification for the 2^nd^ order construct.

**Table 12.** Inter-Construct Correlations for 1^st^ Order TSC Constructs

|  | *Complexity* | *Insecurity* | *Invasion* | *Overload* | *Uncertainty* |
| --- | --- | --- | --- | --- | --- |
| Complexity | 0.766 |  |  |  |  |
| Insecurity | 0.678 | 0.778 |  |  |  |
| Invasion | 0.566 | 0.621 | 0.805 |  |  |
| Overload | 0.633 | 0.684 | 0.727 | 0.788 |  |
| Uncertainty | 0.382 | 0.452 | 0.357 | 0.525 | 0.845 |

**Table 13.** Path Coefficients and Significances for 1^st^ Order DSS Constructs and Criterion Variables

|  | *β* | *t statistic* | *p statistic* |
| --- | --- | --- | --- |
| Complexity -> Emotional Exhaustion | -0.020 | 0.401 | 0.688* |
| Complexity -> Innovation Climate | -0.006 | 0.075 | 0.940* |
| Complexity -> Job Satisfaction | 0.050 | 0.786 | 0.432* |
| Complexity -> User Satisfaction | -0.135 | 2.231 | 0.026 |
| Conflicts -> Emotional Exhaustion | 0.247 | 5.554 | 0.000 |
| Conflicts -> Innovation Climate | -0.076 | 1.307 | 0.191* |
| Conflicts -> Job Satisfaction | -0.091 | 1.526 | 0.127* |
| Conflicts -> User Satisfaction | -0.116 | 2.450 | 0.014 |
| Insecurity -> Emotional Exhaustion | -0.042 | 1.160 | 0.246* |
| Insecurity -> Innovation Climate | 0.048 | 0.865 | 0.387* |
| Insecurity -> Job Satisfaction | -0.120 | 2.433 | 0.015 |
| Insecurity -> User Satisfaction | 0.027 | 0.595 | 0.552* |
| Invasion -> Emotional Exhaustion | -0.068 | 1.710 | 0.087 |
| Invasion -> Innovation Climate | 0.063 | 1.196 | 0.232* |
| Invasion -> Job Satisfaction | 0.070 | 1.522 | 0.128* |
| Invasion -> User Satisfaction | 0.047 | 1.189 | 0.234* |
| Overload -> Emotional Exhaustion | 0.100 | 1.879 | 0.060* |
| Overload -> Innovation Climate | 0.025 | 0.319 | 0.750* |
| Overload -> Job Satisfaction | -0.050 | 0.669 | 0.503* |
| Overload -> User Satisfaction | 0.177 | 3.016 | 0.003 |
| Safety -> Emotional Exhaustion | 0.044 | 1.072 | 0.284* |
| Safety -> Innovation Climate | 0.079 | 1.572 | 0.116* |
| Safety -> Job Satisfaction | 0.040 | 0.909 | 0.363* |
| Safety -> User Satisfaction | 0.012 | 0.282 | 0.778* |
| Social Environment -> Emotional Exhaustion | 0.211 | 4.015 | 0.000 |
| Social Environment -> Innovation Climate | 0.065 | 0.929 | 0.353* |
| Social Environment -> Job Satisfaction | -0.086 | 1.299 | 0.194* |
| Social Environment -> User Satisfaction | 0.021 | 0.383 | 0.702* |
| Technical Support -> Emotional Exhaustion | 0.095 | 1.886 | 0.059* |
| Technical Support -> Innovation Climate | -0.187 | 2.817 | 0.005 |
| Technical Support -> Job Satisfaction | 0.008 | 0.131 | 0.896* |
| Technical Support -> User Satisfaction | 0.106 | 1.985 | 0.047 |
| Unreliability -> Emotional Exhaustion | 0.119 | 2.187 | 0.029 |
| Unreliability -> Innovation Climate | 0.035 | 0.482 | 0.630* |
| Unreliability -> Job Satisfaction | -0.068 | 1.016 | 0.310* |
| Unreliability -> User Satisfaction | -0.289 | 5.087 | 0.000 |
| Usefulness -> Emotional Exhaustion | 0.063 | 1.165 | 0.244* |
| Usefulness -> Innovation Climate | -0.126 | 1.778 | 0.076* |
| Usefulness -> Job Satisfaction | -0.069 | 0.997 | 0.319* |
| Usefulness -> User Satisfaction | -0.276 | 4.723 | 0.000 |
| * indicates non-significant paths based on a p<.05 threshold | | | |

**Table 14.** Results of the CTA-PLS for the 2^nd^ Order DSS Construct

| *Tetrads* | *CI Low adj.* | *CI Up adj.* | *Indicates* |
| --- | --- | --- | --- |
| 1: Complexity, Conflicts, Insecurity, Invasion | -0.175 | -0.048 | Formative |
| 2: Complexity, Conflicts, Invasion, Insecurity | -0.075 | 0.025 | Reflective |
| 4: Complexity, Conflicts, Insecurity, Overload | -0.196 | -0.070 | Formative |
| 6: Complexity, Insecurity, Overload, Conflicts | 0.016 | 0.170 | Formative |
| 7: Complexity, Conflicts, Insecurity, Safety | -0.073 | 0.034 | Reflective |
| 10: Complexity, Conflicts, Insecurity, Social Environment | -0.258 | -0.124 | Formative |
| 13: Complexity, Conflicts, Insecurity, Technical Support | -0.081 | 0.031 | Reflective |
| 17: Complexity, Conflicts, Unreliability, Insecurity | -0.098 | 0.022 | Reflective |
| 20: Complexity, Conflicts, Usefulness, Insecurity | -0.135 | -0.004 | Formative |
| 29: Complexity, Conflicts, Social Environment, Invasion | -0.026 | 0.073 | Reflective |
| 31: Complexity, Conflicts, Invasion, Technical Support | -0.013 | 0.091 | Reflective |
| 35: Complexity, Conflicts, Unreliability, Invasion | -0.153 | -0.023 | Formative |
| 41: Complexity, Conflicts, Safety, Overload | -0.158 | -0.043 | Formative |
| 43: Complexity, Conflicts, Overload, Social Environment | -0.156 | -0.056 | Formative |
| 47: Complexity, Conflicts, Technical Support, Overload | -0.176 | -0.058 | Formative |
| 50: Complexity, Conflicts, Unreliability, Overload | -0.190 | -0.066 | Formative |
| 60: Complexity, Safety, Technical Support, Conflicts | -0.079 | 0.031 | Reflective |
| 64: Complexity, Conflicts, Safety, Usefulness | -0.074 | 0.009 | Reflective |
| 66: Complexity, Safety, Usefulness, Conflicts | -0.112 | 0.017 | Reflective |
| 71: Complexity, Conflicts, Unreliability, Social Environment | -0.237 | -0.101 | Formative |
| 80: Complexity, Conflicts, Usefulness, Technical Support | -0.072 | 0.044 | Reflective |
| 91: Complexity, Insecurity, Invasion, Social Environment | 0.089 | 0.214 | Formative |
| 120: Complexity, Safety, Social Environment, Insecurity | -0.083 | 0.016 | Reflective |
| 169: Complexity, Invasion, Safety, Unreliability | -0.051 | 0.019 | Reflective |
| 182: Complexity, Invasion, Usefulness, Social Environment | -0.230 | -0.100 | Formative |
| 205: Complexity, Overload, Social Environment, Technical Support | -0.017 | 0.060 | Reflective |
| 233: Complexity, Safety, Unreliability, Technical Support | -0.031 | 0.097 | Reflective |
| 236: Complexity, Safety, Usefulness, Technical Support | -0.105 | 0.026 | Reflective |
| 248: Complexity, Social Environment, Usefulness, Unreliability | -0.092 | -0.003 | Formative |
| 281: Conflicts, Insecurity, Unreliability, Overload | -0.034 | 0.067 | Reflective |
| 324: Conflicts, Overload, Technical Support, Invasion | 0.037 | 0.161 | Formative |
| 358: Conflicts, Invasion, Unreliability, Usefulness | 0.028 | 0.156 | Formative |
| 395: Conflicts, Safety, Unreliability, Social Environment | -0.047 | 0.041 | Reflective |
| 434: Insecurity, Invasion, Usefulness, Overload | -0.093 | 0.033 | Reflective |
| 526: Invasion, Overload, Safety, Social Environment | -0.301 | -0.159 | Formative |

**Table 15.** Significance of Loadings for the Reflective Specification of the DSS

| *Path* | *t statistics* | *p statistics* |
| --- | --- | --- |
| Complexity <- DSS | 82.195 | 0.000 |
| Conflicts <- DSS | 62.077 | 0.000 |
| Insecurity <- DSS | 41.115 | 0.000 |
| Invasion <- DSS | 37.686 | 0.000 |
| Overload <- DSS | 111.029 | 0.000 |
| Safety <- DSS | 34.203 | 0.000 |
| Social Environment <- DSS | 88.235 | 0.000 |
| Technical Support <- DSS | 85.223 | 0.000 |
| Unreliability <- DSS | 107.138 | 0.000 |
| Usefulness <- DSS | 108.670 | 0.000 |

**Table 16.** Significance of Weights for the Formative Specification of the DSS

| *Path* | *t statistics* | *p statistics* |
| --- | --- | --- |
| Complexity -> DSS | 0.448 | 0.654* |
| Conflicts -> DSS | 4.497 | 0.000 |
| Insecurity -> DSS | 0.243 | 0.808* |
| Invasion -> DSS | 1.994 | 0.046 |
| Overload -> DSS | 0.095 | 0.925* |
| Safety -> DSS | 0.104 | 0.917* |
| Social Environment -> DSS | 2.449 | 0.014 |
| Technical Support -> DSS | 0.618 | 0.536* |
| Unreliability -> DSS | 3.309 | 0.001 |
| Usefulness -> DSS | 2.886 | 0.004 |
| * Indicates non-significant paths based on <0.05 threshold. | | |

## 2^nd^ Order DSS Construct - Alternatives

### One 1st Order Construct

**Table 17.** Reliability and Validity Statistic for a One-Factor DSS Solution

|  | *α* | *ρA* | *ρc* | *AVE* | *DSS* | *A* | *B* | *C* | *D* |
| --- | --- | --- | --- | --- | --- | --- | --- | --- | --- |
| DSS | 0.984 | 0.988 | 0.986 | 0.370* | *0.608* |  |  |  |  |
| A. Emotional Exhaustion | 0.887 | 0.889 | 0.918 | 0.691 | 0.643 | *0.831* |  |  |  |
| B. Innovation Climate | 0.718 | 0.736 | 0.840 | 0.637 | -0.107 | -0.283 | *0.798* |  |  |
| C. Job Satisfaction | 0.822 | 0.839 | 0.894 | 0.738 | -0.289 | -0.500 | 0.517 | *0.859* |  |
| D. User Satisfaction | 0.923 | 0.932 | 0.946 | 0.813 | -0.397 | -0.357 | 0.396 | 0.462 | *0.902* |
| Values in italics in the right half of the table indicate the square root of the AVE, while values below indicate the inter-construct correlations for the latent variable scores.  * Indicates insufficient AVE (below the .500 threshold). | | | | | | | | | |

**Table 18.** Discriminant Validity based on HTMT for a One-Factor DSS Solution

|  | *DSS* | *Emotional Exhaustion* | *Innovation Climate* | *Job Satisfaction* |
| --- | --- | --- | --- | --- |
| DSS |  |  |  |  |
| Emotional Exhaustion | 0.668 |  |  |  |
| Innovation Climate | 0.172 | 0.357 |  |  |
| Job Satisfaction | 0.312 | 0.577 | 0.662 |  |
| User Satisfaction | 0.406 | 0.393 | 0.490 | 0.524 |

**Table 19.** Item Loadings and Significances for a One-Factor DSS Solution

| *Item* | *Loading* | *t statistic* | *p statistic* |
| --- | --- | --- | --- |
| 1. Due to ICT I have too little to do. | 0.755 | 49.262 | 0.000 |
| 1. Due to ICT my work is too monotonous. | 0.728 | 38.583 | 0.000 |
| 1. Due to ICT I always have to work in accordance with the same tedious routines. | 0.703 | 38.993 | 0.000 |
| 1. ICT automatically rule out too many decision alternatives that I would be willing to try. | 0.697 | 36.474 | 0.000 |
| 1. ICT take away too many of the aspects of my work that I find entertaining. | 0.694 | 35.253 | 0.000 |
| 1. ICT have automatized work tasks that I really enjoyed doing myself. | 0.682 | 35.244 | 0.000 |
| 1. Sometimes I feel bored, because ICT made my job too easy and less thrilling. | 0.634 | 28.681 | 0.000 |
| 1. ICT have made my job less interesting. | 0.604 | 26.915 | 0.000 |
| 1. ICT undertake tasks for me and hence my job is monotonous. | 0.597 | 25.851 | 0.000 |
| 1. I often find it too complicated to accomplish a task using the ICT that are available to me at work. | 0.688 | 36.411 | 0.000 |
| 1. I often need more time than expected to accomplish a task using the ICT that are available to me at work. | 0.610 | 26.789 | 0.000 |
| 1. I feel that the ICT that are available to me at work are too confusing. | 0.744 | 44.459 | 0.000 |
| 1. Often I find the ICT that are available to me at work not user-friendly enough. | 0.670 | 34.504 | 0.000 |
| 1. I often do not find enough time to keep up with new functionalities of ICT at work. | 0.649 | 32.744 | 0.000 |
| 1. There are too many functionalities of the ICT at work that I only require rarely and I need to learn how to use every time again. | 0.633 | 31.407 | 0.000 |
| 1. It would take me too long to completely figure out how to use the ICT that are available to me at work. | 0.704 | 39.351 | 0.000 |
| 1. I feel that using ICT is often a complex process. | 0.622 | 28.072 | 0.000 |
| 1. The complexity involved in using ICT is typically high. | 0.716 | 40.247 | 0.000 |
| 1. ICT enables private problems to reach me too often at work. | 0.595 | 26.961 | 0.000 |
| 1. ICT enables work-related problems to reach me too often at home. | 0.613 | 26.223 | 0.000 |
| 1. I feel that my work routine suffers due to ICT enabling private problems to reach me everywhere. | 0.744 | 48.857 | 0.000 |
| 1. I feel that my private life suffers due to ICT enabling work-related problems to reach me everywhere. | -0.193 | 4.944 | 0.000 |
| 1. It is too hard for me to keep my private life and work life separated due to ICT. | 0.693 | 35.822 | 0.000 |
| 1. ICT make it harder to create clear boundaries between my private life and work life. | 0.733 | 43.283 | 0.000 |
| 1. My work-life balance suffers due to ICT. | 0.018 | 0.420 | 0.675* |
| 1. Distinguishing the use of ICT for work and for private life is difficult. | 0.685 | 34.755 | 0.000 |
| 1. The ubiquity of ICT disturbs my work-life balance. | 0.502 | 18.796 | 0.000 |
| 1. I think it is bad when ICT dictate how I should do my work (e.g., when work routines are highly penetrated by ICT). | 0.723 | 40.247 | 0.000 |
| 1. I think that I am too dependent on ICT at work. | -0.007 | 0.174 | 0.862* |
| 1. Due to ICT I do not have the autonomy at work that I would like to have. | 0.579 | 24.779 | 0.000 |
| 1. I think that I can work more autonomously due to ICT (e.g., when I can accomplish the same task with different ICT). **(-)** | 0.716 | 38.587 | 0.000 |
| 1. I think that ICT have too much impact on the timing of my breaks (i.e., when I can take a break and when not). | 0.599 | 23.907 | 0.000 |
| 1. ICT force me to follow a standardized workflow; I cannot work according to my desired work schedule. | -0.037 | 0.896 | 0.370* |
| 1. ICT give me more flexibility at work; it is easier to reschedule tasks if needed. **(-)** | 0.658 | 32.106 | 0.000 |
| 1. I cannot experiment with new ways of doing my work, as ICT force me into specific work routines. | 0.678 | 37.360 | 0.000 |
| 1. ICT define the way in which I have to do my work. | 0.671 | 31.385 | 0.000 |
| 1. I have to invest more time into the adaptation of ICT to my individual needs than I would like to. | 0.672 | 32.557 | 0.000 |
| 1. I think that the time needed to adapt ICT to my individual needs is worth it. **(-)** | 0.638 | 29.250 | 0.000 |
| 1. I feel uncomfortable when replacing an existing ICT with a new one as I have often already invested a lot of time into their adaptation to my own needs. | 0.617 | 26.973 | 0.000 |
| 1. I often fear using new ICT, because of the cost that would be involved should I not do so. | 0.647 | 30.966 | 0.000 |
| 1. I often feel uncomfortable when I ask for old ICT to be replaced by new ICT as I know that new ICT cost a lot of money. | 0.662 | 32.910 | 0.000 |
| 1. I think that no matter the amount of money invested into ICT, it is always worth it. **(-)** | 0.672 | 32.150 | 0.000 |
| 1. I often feel that setting up new ICT is too much of a hassle. | 0.722 | 39.971 | 0.000 |
| 1. I am often annoyed by ICT updates as they are not worth my time. | 0.606 | 27.938 | 0.000 |
| 1. I think that the time needed to adapt ICT to my individual needs is not worth it. | 0.652 | 30.866 | 0.000 |
| 1. I feel that my job position is threatened due to ICT. | 0.619 | 29.675 | 0.000 |
| 1. I feel that it is threatening that my job could be accomplished in an automated fashion due to ICT. | 0.592 | 27.622 | 0.000 |
| 1. I fear that I could be replaced by individuals with better technology expertise at work. | 0.586 | 25.994 | 0.000 |
| 1. I fear that I could be replaced at work due to the increasing standardization of work processes, which is enabled by ICT. | 0.541 | 22.291 | 0.000 |
| 1. I fear that what I like about my job will one day be obsolete due to ICT. | 0.532 | 21.159 | 0.000 |
| 1. I cannot be optimistic about my long-term job security because of the threat of ICT automatization. | 0.546 | 22.744 | 0.000 |
| 1. It is too hard for me to get involved in my job routine, as I feel that it will change quickly anyway due to ICT. | 0.612 | 28.928 | 0.000 |
| 1. I fear that I could be replaced by machines. | 0.435 | 16.019 | 0.000 |
| 1. I fear that digitalization will cost me my job. | 0.425 | 15.769 | 0.000 |
| 1. If decisions are made in my work environment to use new ICT or replace existing ICT than I am getting too much involved in the decision process. | 0.561 | 19.893 | 0.000 |
| 1. If decisions are made in my work environment to use new ICT or replace existing ICT than I am getting sufficiently involved in the decision process. **(-)** | -0.234 | 6.164 | 0.000 |
| 1. It is frustrating if new ICT is introduced at the workplace without being asked what we think about it beforehand. | 0.551 | 23.351 | 0.000 |
| 1. I am informed too often about the new ICT I have to use at work when there is no time left to do anything about it. | 0.737 | 44.616 | 0.000 |
| 1. I do not get involved enough when the requirements concerning new ICT are assessed. | 0.531 | 19.291 | 0.000 |
| 1. I feel that new ICT is too often forced upon me at work without an opportunity to express my opinion about it. | 0.700 | 39.746 | 0.000 |
| 1. I am annoyed when my demands for new or changed ICT are ignored at work. | 0.567 | 22.722 | 0.000 |
| 1. I am rarely involved in organization decision processes about hardware and software acquisitions. | 0.226 | 7.332 | 0.000 |
| 1. Management involves me in decisions about hardware and software purchases. **(-)** | -0.147 | 4.049 | 0.000 |
| 1. If decisions are made in my work environment to use new ICT or replace existing ICT than I am getting too much involved in the decision process. | 0.733 | 41.818 | 0.000 |
| 1. If decisions are made in my work environment to use new ICT or replace existing ICT than I am getting sufficiently involved in the decision process. **(-)** | 0.699 | 37.174 | 0.000 |
| 1. It is frustrating if new ICT is introduced at the workplace without being asked what we think about it beforehand. | 0.601 | 23.373 | 0.000 |
| 1. I am informed too often about the new ICT I have to use at work when there is no time left to do anything about it. | 0.566 | 22.912 | 0.000 |
| 1. I do not get involved enough when the requirements concerning new ICT are assessed. | 0.730 | 42.798 | 0.000 |
| 1. I feel that new ICT is too often forced upon me at work without an opportunity to express my opinion about it. | 0.524 | 19.551 | 0.000 |
| 1. I am annoyed when my demands for new or changed ICT are ignored at work. | 0.739 | 42.358 | 0.000 |
| 1. I am rarely involved in organization decision processes about hardware and software acquisitions. | 0.367 | 10.992 | 0.000 |
| 1. Management involves me in decisions about hardware and software purchases. **(-)** | 0.704 | 37.642 | 0.000 |
| 1. I fear that my use of ICT is less confidential than I would like to. | 0.533 | 18.351 | 0.000 |
| 1. I fear that I can be more easily monitored due to ICT than I would like to. | 0.707 | 37.831 | 0.000 |
| 1. I fear that I can be more easily monitored by my superiors due to ICT than I would like to. | 0.690 | 36.217 | 0.000 |
| 1. I fear that the information that I exchange using ICT is not as protected as I would like to. | 0.716 | 40.276 | 0.000 |
| 1. I fear that malevolent outsiders (e.g., hackers) can easily copy my identity due to ICT. | 0.717 | 35.290 | 0.000 |
| 1. My personal information is too easily accessible due to ICT. | 0.440 | 13.841 | 0.000 |
| 1. It is unnerving that I can never be sure whether my activity with ICT can be tracked or not. | 0.529 | 18.558 | 0.000 |
| 1. I fear that my personal data can easily be stolen by others online. | 0.678 | 33.624 | 0.000 |
| 1. I fear that my activities on the Internet can be tracked by others. | 0.675 | 31.471 | 0.000 |
| 1. It is too difficult for me to concentrate on my work as I could be disrupted by ICT that I might need for other tasks at any point. | 0.719 | 40.849 | 0.000 |
| 1. It is too difficult for me to concentrate on my work as I am getting constantly disrupted by ICT that I do not actually need to accomplish my tasks. | 0.730 | 43.157 | 0.000 |
| 1. It is too difficult for me to concentrate on my work and cope with ICT-related problems at the same time. | 0.746 | 45.061 | 0.000 |
| 1. Problems with ICT (e.g., technical malfunctions) require more of my attention than I would like to give them. | 0.685 | 37.927 | 0.000 |
| 1. I am too often interrupted by electronic messages (e.g., emails) during work. | 0.592 | 27.528 | 0.000 |
| 1. I think that it is bad, if I am interrupted by electronic media (e.g., due to pop-ups on websites) during work. | 0.438 | 15.865 | 0.000 |
| 1. Due to frequent technical interruptions (e.g., reminders by other systems than the one that I currently work with) I can work in a less orderly fashion than I would like to. | 0.721 | 41.787 | 0.000 |
| 1. I cannot concentrate on my work as much as I want to due to the constant distraction afforded by electronic media (e.g., Facebook). | 0.548 | 21.503 | 0.000 |
| 1. Security measures (e.g., warning messages) interrupt me too often during my work tasks. | 0.696 | 34.727 | 0.000 |
| 1. My colleagues feel encouraged to interrupt me too often during work when I am online in electronic media (e.g., chat tools). | 0.610 | 25.803 | 0.000 |
| 1. The constant multitasking that is needed to handle all the ICT I need for work does not allow me to concentrate on the task at hand sufficiently. | 0.728 | 43.881 | 0.000 |
| 1. ICT make it too easy for new tasks and demands to reach me before I have the chance to finish what I am doing at the moment. | 0.602 | 27.194 | 0.000 |
| 1. I have to worry too often, whether I might download malicious programs. | 0.570 | 23.030 | 0.000 |
| 1. I have to worry too often, whether I might receive malicious e-mails. | 0.606 | 25.750 | 0.000 |
| 1. I have to cope too often with rigorous security measures that are implemented using ICT (e.g., resulting in e-mails being wrongly classified as junk e-mail). | 0.683 | 34.823 | 0.000 |
| 1. I have to worry too often, whether I might damage ICT when interacting with them. | 0.706 | 35.729 | 0.000 |
| 1. Too much time is needed to sort out spam mails during work. | 0.600 | 24.738 | 0.000 |
| 1. I fear that hackers might get access to company secrets through a mistake of mine. | 0.574 | 24.438 | 0.000 |
| 1. I feel anxious when I get an e-mail from somebody that I do not know as it could be a malevolent attack. | 0.500 | 18.935 | 0.000 |
| 1. E-Mails whose sender I do not know make me nervous. | 0.433 | 16.068 | 0.000 |
| 1. A considerable number of e-mails are sent with evil intent. | 0.475 | 17.143 | 0.000 |
| 1. Due to ICT I have too much to do with the problems of others. | 0.720 | 41.471 | 0.000 |
| 1. Due to ICT I have less contact with other people than I would like to. | 0.610 | 25.610 | 0.000 |
| 1. I am too often prompted by people in my close environment to use specific ICT. | 0.667 | 29.902 | 0.000 |
| 1. I think that ICT generate too much of an expectation that I have to be reachable everywhere and at any time. | 0.569 | 24.971 | 0.000 |
| 1. Too much time gets lost at work because of irrelevant communication with other people on social media. | 0.556 | 22.936 | 0.000 |
| 1. I feel that ICT create unwanted social norms (e.g., the expectation that e-mails should be answered right away). | 0.572 | 26.675 | 0.000 |
| 1. It is too hard to take a break from social interactions at work due to the communication possibilities of ICT. | 0.682 | 35.523 | 0.000 |
| 1. ICT helps me to avoid interaction with others. **(-)** | -0.462 | 15.054 | 0.000 |
| 1. Due to ICT I have more contact with beloved individuals. **(-)** | -0.127 | 3.355 | 0.001 |
| 1. I have to worry about ICT-related problems as our organization does not offer enough support for their removal. | 0.731 | 39.901 | 0.000 |
| 1. In the case of ICT-related problems, it happens too often that there is not enough support available at work. | 0.681 | 34.449 | 0.000 |
| 1. I think that it happens too often that technical support is not available when I need it. | -0.125 | 3.227 | 0.001 |
| 1. I often have to wait for a long time because technical problems cannot be adequately solved in our organization. | 0.630 | 28.774 | 0.000 |
| 1. It annoys me if it takes longer than expected for a technical problem to be solved at work. | 0.699 | 38.535 | 0.000 |
| 1. Whenever I encounter a technical problem at work, I blame our incompetent technical support for it. | 0.641 | 28.583 | 0.000 |
| 1. I fear that a technical problem I have at work could not be solved by anyone else at work. | 0.651 | 31.729 | 0.000 |
| 1. The help desk in my company works perfectly. **(-)** | -0.025 | 0.625 | 0.532* |
| 1. The IT service desk members in my company always fulfill my expectations. **(-)** | 0.019 | 0.442 | 0.658* |
| 1. I think that I am too often confronted with unexpected behavior of the ICT I use at work (e.g., breakdowns or long response times). | 0.745 | 42.257 | 0.000 |
| 1. I think that I lose too much time due to technical malfunctions. | 0.684 | 37.507 | 0.000 |
| 1. I think that I spend too much time trying to fix technical malfunctions. | 0.583 | 24.806 | 0.000 |
| 1. There is just too much of my time at work wasted coping with the unreliability of ICT. | 0.682 | 36.185 | 0.000 |
| 1. I fear that a system might crash if I need it most. | 0.429 | 15.728 | 0.000 |
| 1. I fear that a lot of work might get lost if do not save my progress often enough. | 0.561 | 20.884 | 0.000 |
| 1. The daily hassles with ICT (e.g., slow programs or unexpected behavior) are really bothering me. | 0.536 | 20.632 | 0.000 |
| 1. The unreliability of ICT is its most dominant characteristic. | 0.101 | 2.548 | 0.011 |
| 1. I fear that a system breaks down when I need it most. | 0.130 | 3.361 | 0.001 |
| 1. I think that the demands of my work and the functions provided by the ICT I use do not fit sufficiently. | 0.724 | 43.520 | 0.000 |
| 1. I think that I do not gain enough benefits from using the ICT that I am provided with at work for my tasks. | 0.693 | 36.324 | 0.000 |
| 1. At work, I profit from the synergies between my tasks and the functions that are provided by the ICT that I can use. **(-)** | 0.691 | 39.224 | 0.000 |
| 1. The ICT I use at work are full of too many functionalities that I never need. | 0.762 | 50.599 | 0.000 |
| 1. It requires too many different systems to fulfill the tasks that I have to do during an average day at work. | 0.600 | 28.326 | 0.000 |
| 1. I think that most of the ICT I am supplied with at work is not useful enough and I could work without it. | 0.432 | 16.134 | 0.000 |
| 1. I have to constantly switch between systems to fulfill a task, which can get annoying. | 0.683 | 34.801 | 0.000 |
| 1. The ICT at work is ideal, because one single system offers all functionalities that I need to accomplish my work. **(-)** | 0.658 | 30.576 | 0.000 |
| 1. The task-technology fit of the ICT that I use at work is excellent. **(-)** | 0.606 | 27.258 | 0.000 |
| (-) Indicates reverse-scored items.  * Indicates non-significant loadings based on 0.05 threshold. | | | |

### Several 2nd Order Constructs

**Table 20.** Results of the EFA with 1^st^ Order DSS Constructs in SPSS

| *Factor* | *Eigenvalue* | *% of Variance Explained* | *Cumulative % of Variance Explained* |
| --- | --- | --- | --- |
| 1 | 6.698 | 66.984 | 66.984 |
| 2 | .796 | 7.958 | 74.941 |
| 3 | .646 | 6.464 | 81.406 |
| 4 | .486 | 4.860 | 86.266 |
| 5 | .320 | 3.199 | 89.465 |
| 6 | .310 | 3.100 | 92.565 |
| 7 | .208 | 2.079 | 94.644 |
| 8 | .197 | 1.968 | 96.612 |
| 9 | .179 | 1.790 | 98.402 |
| 10 | .160 | 1.598 | 100.000 |
| Extraction method: principal axis; rotation: promax; Converged after 4 iterations | | | |
| Loadings for the first factor: Complexity: .846; Conflicts: .775; Insecurity: .704; Invasion: .671; Overload: .870; Safety: .669; Social Environment: .836; Technical Support: .833; Unreliability: .867; Usefulness: .862 | | | |

## TSC, Criterion Variables and Control Variables

**Table 21.** Loadings for Criterion and Control Variables in both Sub-Samples involving the DSS

| *Model: DSS (2^nd^ Order Construct) – with Controls*  ***Innovation Climate*** *– Source:* Tarafdar, Tu, and Ragu-Nathan (2010)   1. In our organization we have a very open communications environment. 2. In our organization employees and functional managers are supportive of each other. 3. In our organization employees at all levels are rewarded for learning new skills. 4. Management encourages experimental mind-set and risk taking. 5. In our organization new ideas are easy to be implemented.   ***Emotional Exhaustion*** *– Source:* Maslach and Jackson (1981), Schaufeli and Salanova (2007)   1. I feel burned out from my work. 2. I feel used up at the end of the workday. 3. I feel emotionally drained from my work. 4. I feel fatigued when I get up in the morning and have to face another day on the job. 5. Working all day is really a strain for me.   ***Job Satisfaction*** *– Source:* Ragu-Nathan et al. (2008)   1. I like doing the things I do at work. 2. I feel a sense of pride in doing my job. 3. My job is enjoyable.   ***User Satisfaction*** *– Source:* Bhattacherjee (2001), Fuglseth and Sørebø (2014)  How do you feel about your overall experience of utilizing ICT in connection with your work tasks?   1. Very dissatisfied/Very satisfied 2. Very displeased/Very pleased 3. Very frustrated/Very contented 4. Absolutely terrible/Absolutely delighted   ***Computer Self-Efficacy*** *– Source: Compeau and Higgins (1995)*  I could complete my tasks using the new ICT, if…   1. …if there was no one around to tell me what to do as I go. 2. …if I had never used a package like it before. 3. …if I had only the software manuals for reference. 4. …if I had seen someone else using it before trying it myself. 5. …if I could call someone for help if I got stuck. 6. …if someone else had helped me get started. 7. …if I had a lot of time to complete the job for which the new ICT was provided. 8. …if I had just the built-in help facility for assistance. 9. …if someone showed me how to do it first. 10. …if I had used similar packages before this one to do the same job. | *Loadings – Sample 1*  0.816  0.819  -  -  0.761    0.862  0.824  0.864  0.850  0.750  0.876  0.820  0.881  0.921  0.923  0.908  0.854  0.660  0.673  0.709  0.760  0.754  0.727  0.774  0.747  0.714  0.757 | *Loadings – Sample 2*  0.865  0.881  -  -  0.622    0.870  0.837  0.865  0.845  0.776  0.865  0.868  0.862  0.913  0.925  0.895  0.859  0.692  0.632  0.699  0.773  0.770  0.761  0.802  0.777  0.737  0.764 |
| --- | --- | --- |
| - Indicates items that were excluded due to low loadings / high cross-loadings. | | |

**Table 22.** Reliability and Validity Statistics for 1^st^ Order TSC Constructs
and 2^nd^ Order TSC Construct

|  | *α* | *ρA* | *ρc* | *AVE* |
| --- | --- | --- | --- | --- |
| Complexity | 0.823 | 0.836 | 0.876 | 0.586 |
| Insecurity | 0.839 | 0.863 | 0.884 | 0.606 |
| Invasion | 0.821 | 0.839 | 0.880 | 0.649 |
| Overload | 0.847 | 0.860 | 0.891 | 0.620 |
| Uncertainty | 0.799 | 0.802 | 0.882 | 0.713 |
| TSC (2^nd^ Order) | 0.865 | 0.906 | 0.901 | 0.650 |

**Table 23.** Loadings for Criterion and Control Variables in both Sub-Samples involving the TSC

| *Model: TSC (2^nd^ Order) & TSC (1^st^ Order) – with Controls*    ***Technostress Creators*** *– Source:* Ragu-Nathan et al. (2008)   1. *Techno-overload* 2. I am forced by ICT to work much faster. 3. I am forced by ICT to do more work than I can handle. 4. I am forced by ICT to work with very tight time schedules. 5. I am forced to change my work habits to adapt to new technologies. 6. I have a higher workload because of increased technology complexity. 7. *Techno-invasion* 8. I spend less time with my family due to ICT. 9. I have to be in touch with my work even during my vacation due to ICT. 10. I have to sacrifice my vacation and weekend time to keep current on new technologies. 11. I feel my personal life is being invaded by ICT. 12. *Techno-complexity* 13. I do not know enough about ICT to handle my job satisfactorily. 14. I need a long time to understand and use new ICT. 15. I do not find enough time to study and upgrade my technology skills. 16. I find new recruits to this organization know more about ICT than I do. 17. I often find it too complex for me to understand and use ICT. 18. *Techno-insecurity* 19. I feel constant threat to my job security due to ICT. 20. I have to constantly update my technology skills to avoid being replaced. 21. I am threatened by coworkers with newer technology skills. 22. I do not share my ICT-related knowledge with my coworkers for fear of being replaced. 23. I feel there is less sharing of ICT-related knowledge among coworkers for fear of being replaced. 24. *Techno-uncertainty* 25. There are always new developments in the technology sector. 26. There are constant changes in computer software in our organization. 27. There are constant changes in computer hardware in our organization. 28. There are frequent upgrades in computer networks in our organization.   ***Innovation Climate*** *– Source:* Tarafdar et al. (2010)   1. In our organization we have a very open communications environment. 2. In our organization employees and functional managers are supportive of each other. 3. In our organization employees at all levels are rewarded for learning new skills. 4. Management encourages experimental mind-set and risk taking. 5. In our organization new ideas are easy to be implemented.   ***Emotional Exhaustion*** *– Source:* Maslach and Jackson (1981), Schaufeli and Salanova (2007)   1. I feel burned out from my work. 2. I feel used up at the end of the workday. 3. I feel emotionally drained from my work. 4. I feel fatigued when I get up in the morning and have to face another day on the job. 5. Working all day is really a strain for me.   ***Job Satisfaction*** *– Source:* Ragu-Nathan et al. (2008)   1. I like doing the things I do at work. 2. I feel a sense of pride in doing my job. 3. My job is enjoyable.   ***User Satisfaction*** *– Source:* Bhattacherjee (2001), Fuglseth and Sørebø (2014)  How do you feel about your overall experience of utilizing ICT in connection with your work tasks?   1. Very dissatisfied/Very satisfied 2. Very displeased/Very pleased 3. Very frustrated/Very contented 4. Absolutely terrible/Absolutely delighted   ***Computer Self-Efficacy*** *– Source: Compeau and Higgins (1995)*  I could complete my tasks using the new ICT, if…   1. …if there was no one around to tell me what to do as I go. 2. …if I had never used a package like it before. 3. …if I had only the software manuals for reference. 4. …if I had seen someone else using it before trying it myself. 5. …if I could call someone for help if I got stuck. 6. …if someone else had helped me get started. 7. …if I had a lot of time to complete the job for which the new ICT was provided. 8. …if I had just the built-in help facility for assistance. 9. …if someone showed me how to do it first. 10. …if I had used similar packages before this one to do the same job. | *Loadings – Sample 1*  0.898  0.787  0.823  0.836  0.706  0.781  0.850  0.838  0.735  0.817  0.829  0.822  0.778  0.820  0.708  0.664  0.847  0.858  0.841  0.651  0.795  0.784  0.809    0.555  -  0.864  0.829  0.841  0.813  0.821  -  -  0.762  0.861  0.823  0.866  0.849  0.751  0.872  0.834  0.871  0.922  0.923  0.907  0.853  0.661  0.671  0.708  0.759  0.753  0.729  0.775  0.745  0.716  0.759 | *Loadings – Sample 2*  0.884    0.834  0.839  0.879  0.465    0.875  0.883  -  -  0.567  0.868  0.838  0.865  0.842  0.780  0.859  0.881  0.851  0.914  0.925  0.897  0.854  0.685  0.620  0.695  0.775  0.775  0.769  0.805  0.773  0.744  0.769 |
| --- | --- | --- |
| - Indicates items that were excluded due to low loadings / high cross-loadings. | | |

**Table 24.** Significance of Loadings for TSC 1^st^ Order Constructs and 2^nd^ Order Construct
based on Sub-Sample 1

| *Path* | *t statistics* | *p statistics* |
| --- | --- | --- |
| Overload 1 <- Overload | 43.497 | 0.000 |
| Overload 2 <- Overload | 62.735 | 0.000 |
| Overload 3 <- Overload | 77.191 | 0.000 |
| Overload 4 <- Overload | 32.912 | 0.000 |
| Overload 5 <- Overload | 46.248 | 0.000 |
| Invasion 1 <- Invasion | 70.374 | 0.000 |
| Invasion 2 <- Invasion | 30.983 | 0.000 |
| Invasion 3 <- Invasion | 57.769 | 0.000 |
| Invasion 4 <- Invasion | 69.157 | 0.000 |
| Complexity 1 <- Complexity | 44.180 | 0.000 |
| Complexity 2 <- Complexity | 56.643 | 0.000 |
| Complexity 3 <- Complexity | 35.913 | 0.000 |
| Complexity 4 <- Complexity | 24.366 | 0.000 |
| Complexity 5 <- Complexity | 74.439 | 0.000 |
| Insecurity 1 <- Insecurity | 76.617 | 0.000 |
| Insecurity 2 <- Insecurity | 22.321 | 0.000 |
| Insecurity 3 <- Insecurity | 48.456 | 0.000 |
| Insecurity 4 <- Insecurity | 39.081 | 0.000 |
| Insecurity 5 <- Insecurity | 50.713 | 0.000 |
| Uncertainty 2 <- Uncertainty | 42.083 | 0.000 |
| Uncertainty 3 <- Uncertainty | 35.487 | 0.000 |
| Uncertainty 4 <- Uncertainty | 24.295 | 0.000 |
| Complexity <- TSC | 58.625 | 0.000 |
| Insecurity <- TSC | 71.412 | 0.000 |
| Invasion <- TSC | 88.392 | 0.000 |
| Overload <- TSC | 137.750 | 0.000 |
| Uncertainty <- TSC | 16.328 | 0.000 |

**Table 25.** Discriminant Validity based on Fornell-Larcker Criterion for 1^st^ Order TSC Constructs

|  | *Complexity* | *Insecurity* | *Invasion* | *Overload* | *Uncertainty* | *A* | *B* | *C* | *D* |
| --- | --- | --- | --- | --- | --- | --- | --- | --- | --- |
| Complexity | *0.766* |  |  |  |  |  |  |  |  |
| Insecurity | 0.678 | *0.778* |  |  |  |  |  |  |  |
| Invasion | 0.566 | 0.621 | *0.805* |  |  |  |  |  |  |
| Overload | 0.633 | 0.684 | 0.727 | *0.788* |  |  |  |  |  |
| Uncertainty | 0.382 | 0.452 | 0.357 | 0.525 | *0.845* |  |  |  |  |
| A. Emotional Exhaustion | 0.436 | 0.436 | 0.553 | 0.561 | 0.202 | *0.831* |  |  |  |
| B. Innovation Climate | -0.035 | 0.010 | -0.031 | -0.049 | 0.146 | -0.291 | *0.797* |  |  |
| C. Job Satisfaction | -0.187 | -0.199 | -0.232 | -0.234 | 0.042 | -0.498 | 0.507 | *0.859* |  |
| D. User Satisfaction | -0.342 | -0.236 | -0.248 | -0.293 | -0.043 | -0.357 | 0.399 | 0.460 | *0.902* |
| Values in italics indicate the square root of the AVE, while values below indicate the inter-construct correlations of the latent variable scores. | | | | | | | | | |

**Table 26.** Discriminant Validity based on HTMT for 1^st^ Order TSC Constructs

|  | *Complexity* | *Insecurity* | *Invasion* | *Overload* | *Uncertainty* | *A* | *B* | *C* | *D* |
| --- | --- | --- | --- | --- | --- | --- | --- | --- | --- |
| Complexity | - |  |  |  |  |  |  |  |  |
| Insecurity | 0.812 | - |  |  |  |  |  |  |  |
| Invasion | 0.678 | 0.739 | - |  |  |  |  |  |  |
| Overload | 0.753 | 0.814 | 0.860* | - |  |  |  |  |  |
| Uncertainty | 0.480 | 0.584 | 0.444 | 0.649 | - |  |  |  |  |
| A. Emotional Exhaustion | 0.501 | 0.491 | 0.640 | 0.642 | 0.241 | - |  |  |  |
| B. Innovation Climate | 0.085 | 0.112 | 0.089 | 0.073 | 0.185 | 0.357 | - |  |  |
| C. Job Satisfaction | 0.218 | 0.221 | 0.272 | 0.272 | 0.060 | 0.577 | 0.662 | - |  |
| D. User Satisfaction | 0.380 | 0.246 | 0.269 | 0.323 | 0.079 | 0.393 | 0.490 | 0.524 | - |
| * Indicates values that fall below the .900 threshold, but do not fulfill the more conservative .850 threshold. | | | | | | | | | |

**Table 27.** Discriminant Validity based on Fornell-Larcker Criterion for 2^nd^ Order TSC Construct

|  | *TSC* | *A* | *B* | *C* | *D* |
| --- | --- | --- | --- | --- | --- |
| TSC | *0.806* |  |  |  |  |
| A. Emotional Exhaustion | 0.575 | *0.831* |  |  |  |
| B. Innovation Climate | 0.066 | 0.194 | *0.675* |  |  |
| C. Job Satisfaction | -0.236 | -0.497 | -0.435 | *0.859* |  |
| D. User Satisfaction | -0.315 | -0.357 | -0.292 | 0.461 | *0.902* |
| Values in italics indicate the square root of the AVE, while values below indicate the inter-construct correlations for the latent variable scores. | | | | | |

**Table 28.** Discriminant Validity based on HTMT for 2^nd^ TSC Order Construct

|  | *TSC* | *A* | *B* | *C* | *D* |
| --- | --- | --- | --- | --- | --- |
| TSC | - |  |  |  |  |
| A. Emotional Exhaustion | 0.619 | - |  |  |  |
| B. Innovation Climate | 0.107 | 0.357 | - |  |  |
| C. Job Satisfaction | 0.262 | 0.577 | 0.662 | - |  |
| D. User Satisfaction | 0.319 | 0.393 | 0.490 | 0.524 | - |

# Structural Model Evaluation

In this section, structural models involving the DSS and TSC and related reliability and validity statistics as well as metrics related to explanatory power and predictive relevance are reported. Calculations in this section are based on sub-sample 2 unless indicated otherwise.

**Table 29.** Comparison of Latent Variable Scores in both Sub-Samples

| Construct | Mean Rank | | U statistic | Asympt. Sig. |
| --- | --- | --- | --- | --- |
|  | Sample 1 | Sample 2 |  |  |
| Computer Self-Efficacy | 1,001.35 | 997.59 | 496,975.500 | .884 |
| DSS | 997.93 | 1,001.12 | 500,451.500 | .902 |
| Emotional Exhaustion | 994.14 | 1,005.04 | 504,297.500 | .673 |
| Innovation Climate | 999.66 | 999.33 | 498,692.000 | .990 |
| Job Satisfaction | 1,011.49 | 987.09 | 486,671.000 | .344 |
| User Satisfaction | 1,012.25 | 986.31 | 485,903.500 | .315 |

## DSS – Structural Models

### Model 1: DSS Main Effects

**Table 30.** Reliability and Validity Statistics for Latent Variables in Sub-Sample 2
(DSS – Main Effects)

|  | α | ρA | ρc | AVE | DSS | A | B | C | D |
| --- | --- | --- | --- | --- | --- | --- | --- | --- | --- |
| DSS | 0.943 | 0.951 | 0.951 | 0.663 | 0.814 |  |  |  |  |
| A. Emotional Exhaustion | 0.894 | 0.895 | 0.922 | 0.704 | 0.640 | 0.839 |  |  |  |
| B. Innovation Climate | 0.739 | 0.897 | 0.826 | 0.620 | -0.232 | -0.341 | 0.788 |  |  |
| C. Job Satisfaction | 0.833 | 0.837 | 0.899 | 0.749 | -0.274 | -0.504 | 0.486 | 0.865 |  |
| D. User Satisfaction | 0.920 | 0.927 | 0.944 | 0.807 | -0.438 | -0.409 | 0.397 | 0.467 | 0.898 |
| Values in italics in the right half of the table indicate the square root of the AVE, while values below indicate the inter-construct correlations of the latent variable scores. | | | | | | | | | |

**Table 31.** Discriminant Validity based on HTMT in Sub-Sample 2 (DSS – Main Effects)

|  | *DSS* | *Emotional Exhaustion* | *Innovation Climate* | *Job Satisfaction* |
| --- | --- | --- | --- | --- |
| DSS | - |  |  |  |
| Emotional Exhaustion | 0.691 | - |  |  |
| Innovation Climate | 0.221 | 0.392 | - |  |
| Job Satisfaction | 0.301 | 0.580 | 0.621 | - |
| User Satisfaction | 0.461 | 0.449 | 0.499 | 0.533 |

**Table 32.** Path Coefficients and Significance (DSS – Main Effects)

|  | *β* | *t statistics* | *p statistics* |
| --- | --- | --- | --- |
| DSS -> Emotional Exhaustion | 0.640 | 32.991 | 0.000 |
| DSS -> Innovation Climate | -0.232 | 7.955 | 0.000 |
| DSS -> Job Satisfaction | -0.274 | 9.038 | 0.000 |
| DSS -> User Satisfaction | -0.438 | 14.672 | 0.000 |

**Table 33.** Explained Variance (DSS – Main Effects)

|  | *R Square* | *R Square Adjusted* |
| --- | --- | --- |
| Emotional Exhaustion | 0.409 | 0.409 |
| Innovation Climate | 0.054 | 0.053 |
| Job Satisfaction | 0.075 | 0.074 |
| User Satisfaction | 0.192 | 0.191 |

### Model 2: DSS with Controls

**Table 34.** Reliability and Validity Statistics for Latent Variables in Sub-Sample 2
(DSS – With Controls)

|  | α | ρA | ρc | AVE | CSE | DSS | A | B | C | D |
| --- | --- | --- | --- | --- | --- | --- | --- | --- | --- | --- |
| Computer Self-Efficacy (CSE) | 0.909 | 0.915 | 0.924 | 0.551 | 0.742 |  |  |  |  |  |
| DSS | 0.943 | 0.951 | 0.951 | 0.663 | -0.159 | 0.814 |  |  |  |  |
| A. Emotional Exhaustion | 0.894 | 0.896 | 0.922 | 0.704 | -0.053 | 0.639 | 0.839 |  |  |  |
| B. Innovation Climate | 0.739 | 0.819 | 0.837 | 0.637 | 0.189 | -0.220 | -0.334 | 0.798 |  |  |
| C. Job Satisfaction | 0.833 | 0.838 | 0.899 | 0.748 | 0.147 | -0.272 | -0.497 | 0.485 | 0.865 |  |
| D. User Satisfaction | 0.920 | 0.925 | 0.944 | 0.807 | 0.240 | -0.438 | -0.409 | 0.401 | 0.464 | 0.898 |
| Values in italics in the right half of the table indicate the square root of the AVE, while values below indicate the inter-construct correlations of the latent variable scores. | | | | | | | | | | |

**Table 35.** Discriminant Validity based on HTMT in Sub-Sample 2 (DSS – With Controls)

|  | *Computer Self-Efficacy* | *DSS* | *Emotional Exhaustion* | *Innovation Climate* | *Job Satisfaction* |
| --- | --- | --- | --- | --- | --- |
| DSS | 0.164 |  |  |  |  |
| Emotional Exhaustion | 0.064 | 0.691 |  |  |  |
| Innovation Climate | 0.201 | 0.221 | 0.392 |  |  |
| Job Satisfaction | 0.159 | 0.301 | 0.580 | 0.621 |  |
| User Satisfaction | 0.255 | 0.461 | 0.449 | 0.499 | 0.533 |

**Table 36.** Path Coefficients and Significance (DSS – With Controls)

|  | *β* | *t statistics* | *p statistics* |
| --- | --- | --- | --- |
| Age -> DSS | -0.122 | 3.800 | 0.000 |
| Age -> Emotional Exhaustion | -0.137 | 5.584 | 0.000 |
| Age -> Innovation Climate | -0.053 | 1.586 | 0.113* |
| Age -> Job Satisfaction | 0.159 | 5.423 | 0.000 |
| Age -> User Satisfaction | -0.088 | 3.046 | 0.002 |
| Computer Self-Efficacy -> DSS | -0.164 | 5.229 | 0.000 |
| Computer Self-Efficacy -> Emotional Exhaustion | 0.046 | 1.872 | 0.061* |
| Computer Self-Efficacy -> Innovation Climate | 0.152 | 4.840 | 0.000 |
| Computer Self-Efficacy -> Job Satisfaction | 0.115 | 3.508 | 0.001 |
| Computer Self-Efficacy -> User Satisfaction | 0.169 | 6.081 | 0.000 |
| DSS -> Emotional Exhaustion | 0.636 | 31.112 | 0.000 |
| DSS -> Innovation Climate | -0.204 | 5.939 | 0.000 |
| DSS -> Job Satisfaction | -0.238 | 7.348 | 0.000 |
| DSS -> User Satisfaction | -0.421 | 13.985 | 0.000 |
| Education -> DSS | 0.046 | 1.410 | 0.159* |
| Education -> Emotional Exhaustion | -0.035 | 1.397 | 0.162* |
| Education -> Innovation Climate | -0.005 | 0.146 | 0.884* |
| Education -> Job Satisfaction | 0.024 | 0.740 | 0.459* |
| Education -> User Satisfaction | -0.025 | 0.838 | 0.402* |
| Gender -> DSS | -0.050 | 1.613 | 0.107* |
| Gender -> Emotional Exhaustion | 0.088 | 3.608 | 0.000 |
| Gender -> Innovation Climate | -0.095 | 2.951 | 0.003 |
| Gender -> Job Satisfaction | 0.008 | 0.246 | 0.806* |
| Gender -> User Satisfaction | -0.060 | 2.146 | 0.032 |
| * Indicates non-significant paths that do not meet the <0.05 threshold. | | | |

**Table 37.** Effect Sizes (DSS – With Controls)

|  | *DSS* | *Emotional Exhaustion* | *Innovation Climate* | *Job Satisfaction* | *User Satisfaction* |
| --- | --- | --- | --- | --- | --- |
| DSS | - | 0.699 | 0.043 | 0.061 | 0.221 |
| Age | 0.015 | 0.032 | 0.003 | 0.027 | 0.009 |
| Computer Self-Efficacy | 0.028 | 0.004 | 0.025 | 0.014 | 0.036 |
| Education | 0.002 | 0.002 | 0.000 | 0.001 | 0.001 |
| Gender | 0.003 | 0.014 | 0.010 | 0.000 | 0.004 |
| R² | 0.042 | 0.445 | 0.083 | 0.112 | 0.232 |
| R² Adjusted | 0.038 | 0.442 | 0.078 | 0.107 | 0.228 |
| Values above the bold line are f² statistics. | | | | | |

## TSC – Structural Models

### Model 3: TSC Main Effects

**Table 38.** Reliability and Validity Statistics for Latent Variables in Sub-Sample 2
(TSC – Main Effects)

|  | α | ρA | ρc | AVE | TSC | A | B | C | D |
| --- | --- | --- | --- | --- | --- | --- | --- | --- | --- |
| TSC | 0.853 | 0.897 | 0.893 | 0.634 | 0.796 |  |  |  |  |
| A. Emotional Exhaustion | 0.894 | 0.895 | 0.922 | 0.704 | 0.597 | 0.839 |  |  |  |
| B. Innovation Climate | 0.739 | 0.519 | 0.773 | 0.555 | -0.216 | -0.334 | 0.745 |  |  |
| C. Job Satisfaction | 0.833 | 0.833 | 0.900 | 0.749 | -0.222 | -0.499 | 0.458 | 0.866 |  |
| D. User Satisfaction | 0.920 | 0.934 | 0.943 | 0.806 | -0.373 | -0.409 | 0.366 | 0.466 | 0.898 |
| Values in italics in the right half of the table indicate the square root of the AVE, while values below indicate the inter-construct correlations of the latent variable scores. | | | | | | | | | |

**Table 39.** Discriminant Validity based on HTMT in Sub-Sample 2 (TSC – Main Effects)

|  | *TSC* | *Emotional Exhaustion* | *Innovation Climate* | *Job Satisfaction* |
| --- | --- | --- | --- | --- |
| TSC | - |  |  |  |
| Emotional Exhaustion | 0.649 | - |  |  |
| Innovation Climate | 0.231 | 0.392 | - |  |
| Job Satisfaction | 0.247 | 0.580 | 0.621 | - |
| User Satisfaction | 0.372 | 0.449 | 0.499 | 0.533 |

**Table 40.** Path Coefficients and Significance (TSC – Main Effects)

|  | *β* | *t statistics* | *p statistics* |
| --- | --- | --- | --- |
| TSC -> Emotional Exhaustion | 0.597 | 29.661 | 0.000 |
| TSC -> Innovation Climate | -0.216 | 3.363 | 0.001 |
| TSC -> Job Satisfaction | -0.222 | 7.149 | 0.000 |
| TSC -> User Satisfaction | -0.373 | 11.914 | 0.000 |

**Table 41.** Explained Variance (TSC – Main Effects)

|  | *R Square* | *R Square Adjusted* |
| --- | --- | --- |
| Emotional Exhaustion | 0.356 | 0.326 |
| Innovation Climate | 0.047 | 0.046 |
| Job Satisfaction | 0.049 | 0.048 |
| User Satisfaction | 0.139 | 0.139 |

### Model 4: TSC with Controls

**Table 42.** Reliability and Validity Statistics for Latent Variables in Sub-Sample 2
(TSC – With Controls)

|  | α | ρA | ρc | AVE | CSE | TSC | A | B | C | D |
| --- | --- | --- | --- | --- | --- | --- | --- | --- | --- | --- |
| Computer Self-Efficacy (CSE) | 0.909 | 0.914 | 0.924 | 0.551 | 0.743 |  |  |  |  |  |
| TSC | 0.945 | 0.950 | 0.953 | 0.669 | -0.198 | 0.796 |  |  |  |  |
| A. Emotional Exhaustion | 0.894 | 0.896 | 0.922 | 0.704 | -0.053 | 0.596 | 0.839 |  |  |  |
| B. Innovation Climate | 0.739 | 0.819 | 0.838 | 0.638 | 0.195 | -0.179 | -0.332 | 0.789 |  |  |
| C. Job Satisfaction | 0.833 | 0.836 | 0.899 | 0.749 | 0.150 | -0.222 | -0.491 | 0.478 | 0.864 |  |
| D. User Satisfaction | 0.920 | 0.925 | 0.944 | 0.807 | 0.238 | -0.372 | -0.409 | 0.393 | 0.462 | 0.898 |
| Values in italics in the right half of the table indicate the square root of the AVE, while values below indicate the inter-construct correlations of the latent variable scores. | | | | | | | | | | |

**Table 43.** Discriminant Validity based on HTMT in Sub-Sample 2 (TSC – With Controls)

|  | *Computer Self-Efficacy* | *TSC* | *Emotional Exhaustion* | *Innovation Climate* | *Job Satisfaction* |
| --- | --- | --- | --- | --- | --- |
| TSC | 0.205 |  |  |  |  |
| Emotional Exhaustion | 0.064 | 0.649 |  |  |  |
| Innovation Climate | 0.201 | 0.231 | 0.392 |  |  |
| Job Satisfaction | 0.159 | 0.247 | 0.580 | 0.621 |  |
| User Satisfaction | 0.255 | 0.372 | 0.449 | 0.499 | 0.533 |

**Table 44.** Path Coefficients and Significance (TSC – With Controls)

|  | *β* | *t statistics* | *p statistics* |
| --- | --- | --- | --- |
| Age -> TSC | -0.104 | 5.988 | 0.000 |
| Age -> Emotional Exhaustion | -0.153 | 1.089 | 0.276* |
| Age -> Innovation Climate | -0.038 | 5.810 | 0.000 |
| Age -> Job Satisfaction | 0.172 | 3.145 | 0.002 |
| Age -> User Satisfaction | -0.072 | 2.471 | 0.014 |
| Computer Self-Efficacy -> TSC | -0.203 | 2.563 | 0.010 |
| Computer Self-Efficacy -> Emotional Exhaustion | 0.064 | 4.988 | 0.000 |
| Computer Self-Efficacy -> Innovation Climate | 0.161 | 3.505 | 0.000 |
| Computer Self-Efficacy -> Job Satisfaction | 0.119 | 6.455 | 0.000 |
| Computer Self-Efficacy -> User Satisfaction | 0.165 | 5.533 | 0.000 |
| TSC -> Emotional Exhaustion | 0.601 | 0.969 | 0.333* |
| TSC -> Innovation Climate | -0.154 | 0.183 | 0.854* |
| TSC -> Job Satisfaction | -0.184 | 0.590 | 0.555* |
| TSC -> User Satisfaction | -0.348 | 1.053 | 0.293* |
| Education -> TSC | 0.034 | 1.052 | 0.293* |
| Education -> Emotional Exhaustion | -0.026 | 3.719 | 0.000 |
| Education -> Innovation Climate | -0.006 | 2.961 | 0.003 |
| Education -> Job Satisfaction | 0.020 | 0.238 | 0.812* |
| Education -> User Satisfaction | -0.032 | 2.039 | 0.041 |
| Gender -> TSC | -0.064 | 2.121 | 0.034 |
| Gender -> Emotional Exhaustion | 0.095 | 28.683 | 0.000 |
| Gender -> Innovation Climate | -0.096 | 4.197 | 0.000 |
| Gender -> Job Satisfaction | 0.007 | 5.862 | 0.000 |
| Gender -> User Satisfaction | -0.061 | 10.970 | 0.000 |
| * Indicates non-significant paths that do not meet the <0.05 threshold. | | | |

**Table 45.** Effect Sizes (TSC – With Controls)

|  | *TSC* | *Emotional Exhaustion* | *Innovation Climate* | *Job Satisfaction* | *User Satisfaction* |
| --- | --- | --- | --- | --- | --- |
| TSC | - | 0.568 | 0.024 | 0.036 | 0.139 |
| Age | 0.011 | 0.037 | 0.001 | 0.031 | 0.006 |
| Computer Self-Efficacy | 0.043 | 0.007 | 0.026 | 0.015 | 0.032 |
| Education | 0.001 | 0.001 | 0.000 | 0.000 | 0.001 |
| Gender | 0.004 | 0.014 | 0.010 | 0.000 | 0.004 |
| R² | 0.053 | 0.398 | 0.068 | 0.092 | 0.176 |
| R² Adjusted | 0.049 | 0.395 | 0.063 | 0.087 | 0.172 |
| Values above the bold line are f² statistics. | | | | | |

## Hierarchical Regressions

To directly compare TSC and DSS, we created a number of hierarchical regressions for all four criterion variables in SPSS, with control variables being entered in the first step, the TSC in the second step, and the DSS in the third step. The latent variable scores for TSC and DSS were calculated using SmartPLS and a structural model that included TSC, DSS, all control variables and all four criterion variables, based on the data of the second sub-sample.

**Table 46.** Hierarchical Regression for Emotional Exhaustion

| EMEX | Step 1 (Control variables) | | | Step 2 (TSC) | | | Step 3 (DSS) | | |
| --- | --- | --- | --- | --- | --- | --- | --- | --- | --- |
|  | B | SE B | β | B | SE B | β | B | SE B | β |
| Age | -14.321 | 2.116 | -.215** | -10.283 | 1.691 | -.155** | -9.219 | 1.636 | -.139** |
| Gender | 114.171 | 63.627 | .056 | 191.415 | 50.700 | .095** | 182.039 | 48.896 | .090** |
| Education | -2.973 | 16.255 | -.006 | -14.010 | 12.934 | -.027 | -17.765 | 12.479 | -.034 |
| CSE | -.058 | .031 | -.058 | .069 | .025 | .069** | .053 | .024 | .053* |
| TSC |  |  |  | .606 | .025 | .606** | .104 | .063 | .104 |
| DSS |  |  |  |  |  |  | .542 | .063 | .542** |
| r² |  |  | .052 |  |  | .401 |  | . | .443 |
| ∆ r² |  |  |  |  |  | .348 |  |  | .042 |
| ∆ F |  |  |  |  |  | 569,010 |  |  | 74.843 |
| Sig. ∆ F |  |  |  |  |  | < .001 |  |  | < .001 |
| * = p < .05; ** = p < .01 | | | | | | | | | |

**Table 47.** Hierarchical Regression for Innovation Climate

| INNO | Step 1 (Control variables) | | | Step 2 (TSC) | | | Step 3 (DSS) | | |
| --- | --- | --- | --- | --- | --- | --- | --- | --- | --- |
|  | B | SE B | β | B | SE B | β | B | SE B | β |
| Age | -2.395 | 2.133 | -.036 | -3.356 | 2.124 | -.050 | -4.171 | 2.102 | -.063* |
| Gender | -171.742 | 64.315 | -.085** | -190.117 | 63.636 | -.094** | -182.941 | 62.834 | -.091** |
| Education | -9.214 | 16.385 | -.018 | -6.589 | 16.235 | -.013 | -3.715 | 16.036 | -.007 |
| CSE | .178 | .031 | .178** | .148 | .032 | .148** | .161 | .031 | .161** |
| TSC |  |  |  | -.144 | .032 | -.144* | .240 | .081 | .240** |
| DSS |  |  |  |  |  |  | -.415 | .080 | -.415** |
| r² |  |  | .037 |  |  | .056 |  |  | .080 |
| ∆ r² |  |  |  |  |  | .020 |  |  | .025 |
| ∆ F |  |  |  |  |  | 20.439 |  |  | 26.550 |
| Sig. ∆ F |  |  |  |  |  | < .001 |  |  | < .001 |
| * = p < .05; ** = p < .01 | | | | | | | | | |

**Table 48.** Hierarchical Regression for Job Satisfaction

| JOSA | Step 1 (Control variables) | | | Step 2 (TSC) | | | Step 3 (DSS) | | |
| --- | --- | --- | --- | --- | --- | --- | --- | --- | --- |
|  | B | SE B | β | B | SE B | β | B | SE B | β |
| Age | 12.471 | 2.116 | .187** | 11.175 | 2.087 | .168** | 10.385 | 2.067 | .156** |
| Gender | 38.822 | 63.615 | .019 | 14.029 | 62.556 | .007 | 20.992 | 61.790 | .010 |
| Education | 6.981 | 16.252 | .014 | 10.524 | 15.959 | .020 | 13.312 | 15.769 | .026 |
| CSE | .152 | .031 | .152** | .111 | .031 | .111** | .123 | .031 | .123** |
| TSC |  |  |  | -.195 | .031 | -.195** | .179 | .080 | .179* |
| DSS |  |  |  |  |  |  | -.402 | .079 | -.402** |
| r² |  |  | .053 |  |  | .088 |  |  | .110 |
| ∆ r² |  |  |  |  |  | .036 |  |  | .023 |
| ∆ F |  |  |  |  |  | 38.505 |  |  | 25.848 |
| Sig. ∆ F |  |  |  |  |  | < .001 |  |  | < .001 |
| * = p < .05; ** = p < .01 | | | | | | | | | |

**Table 49.** Hierarchical Regression for User Satisfaction

| USSA | Step 1 (Control variables) | | | Step 2 (TSC) | | | Step 3 (DSS) | | |
| --- | --- | --- | --- | --- | --- | --- | --- | --- | --- |
|  | B | SE B | β | B | SE B | β | B | SE B | β |
| Age | -2.447 | 2.111 | -.037 | -4.805 | 1.984 | -.072* | -6.061 | 1.918 | -.091** |
| Gender | -79.007 | 63.461 | -.039 | -124.126 | 59.470 | -.061* | -113.059 | 57.327 | -.056* |
| Education | -22.871 | 16.213 | -.044 | -16.424 | 15.172 | -.032 | -11.993 | 14.630 | -.023 |
| CSE | .235 | .031 | .235** | .161 | .030 | .161** | .180 | .029 | .180** |
| TSC |  |  |  | -.354 | .030 | -.354** | .239 | .074 | .239** |
| DSS |  |  |  |  |  |  | -.639 | .073 | -.639** |
| r² |  |  | .057 |  |  | .176 |  |  | .234 |
| ∆ r² |  |  |  |  |  | .119 |  |  | .059 |
| ∆ F |  |  |  |  |  | 141.102 |  |  | 75.875 |
| Sig. ∆ F |  |  |  |  |  | < .001 |  |  | < .001 |
| * = p < .05; ** = p < .01 | | | | | | | | | |

References

Bhattacherjee, A. (2001). Understanding Information Systems Continuance: An Expectation-Confirmation Model. *MIS Quarterly*, *25*(3), 351–370. https://doi.org/10.2307/3250921

Bosma, H., Peter, R., Siegrist, J., & Marmot, M. (1998). Two Alternative Job Stress Models and the Risk of Coronary Heart Disease. *American Journal of Public Health*, *88*(1), 68–74.

Coltman, T., Devinney, T. M., Midgley, D. F., & Venaik, S. (2008). Formative versus reflective measurement models: Two applications of formative measurement. *Journal of Business Research*, *61*(12), 1250–1262. https://doi.org/10.1016/j.jbusres.2008.01.013

Compeau, D. R., & Higgins, C. A. (1995). Computer Self-Efficacy: Development of a Measure and Initial Test. *MIS Quarterly*, *19*(2), 189–211. https://doi.org/10.2307/249688

Cooper, C. L., & Cartwright, S. (1994). Healthy Mind; Healthy Organization - A Proactive Approach to Occupational Stress. *Human Relations*, *47*(4), 455–471.

Cooper, C. L., & Payne, R. (Eds.) (1978). *Stress at work*. *Wiley series on studies in occupational stress*. Chichester, New York: Wiley.

Edwards, J. R. (2001). Multidimensional Constructs in Organizational Behavior Research: An Integrative Analytical Framework. *Organizational Research Methods*, *4*(2), 144–192. https://doi.org/10.1177/109442810142004

Fuglseth, A. M., & Sørebø, Ø. (2014). The Effects of Technostress within the Context of Employee Use of ICT. *Computers in Human Behavior*, *40*, 161–170.

Gudergan, S. P., Ringle, C. M., Wende, S., & Will, A. (2008). Confirmatory tetrad analysis in PLS path modeling. *Journal of Business Research*, *61*(12), 1238–1249. https://doi.org/10.1016/j.jbusres.2008.01.012

Hudiburg, R. A. (1995). Psychology of Computer Use. XXXIV. The Computer Hassles Scale: Subscales, Norms, and Reliability. *Psychological Reports*, *77*(3), 779–782.

Ivancevich, J. M., & Matteson, M. T. (1980). *Stress and Work: A Managerial Perspective*. *Management applications series*. Glenview, Ill.: Scott, Foresman.

Kahn, R. L., & Byosiere, P. (1992). Stress in Organizations. In M. D. Dunnette & L. M. Hough (Eds.), *Handbook of industrial and organizational psychology* (2nd ed., pp. 571–650). Palo Alto, Calif: Consulting Psychologists Press.

Karasek, R. A. J., Brisson, C., Kawakami, N., Houtman, I., Bongers, P., & Amick, B. (1998). The Job Content Questionnaire (JCQ): An Instrument for Internationally Comparative Assessments of Psychosocial Job Characteristics. *Journal of Occupational Health Psychology*, *3*(4), 322–355.

Marshall, J., & Cooper, C. L. (1979). *Executives under pressure: A psychological study*. New York: Praeger.

Maslach, C., & Jackson, S. E. (1981). The measurement of experienced burnout. *Journal of Organizational Behavior*, *2*(2), 99–113. https://doi.org/10.1002/job.4030020205

Parker, D. F., & DeCotiis, T. A. (1983). Organizational Determinants of Job Stress. *Organizational Behavior and Human Performance*, *32*, 160–177.

Peters, L. H., & O‘Connor, E. J. (1980). Situational Constraints and Work Outcomes: The Influences of a Frequently Overlooked Construct. *The Academy of Management Review*, *5*(3), 391–397. https://doi.org/10.2307/257114

Ragu-Nathan, T. S., Tarafdar, M., Ragu-Nathan, B. S., & Tu, Q. (2008). The Consequences of Technostress for End Users in Organizations: Conceptual Development and Empirical Validation. *Information Systems Research*, *19*(4), 417–433.

Riedl, R., Kindermann, H., Auinger, A., & Javor, A. (2012). Technostress from a Neurobiological Perspective - System Breakdown Increases the Stress Hormone Cortisol in Computer Users. *Business & Information Systems Engineering*, *4*(2), 61–69.

Sarstedt, M., Hair, J. F., Cheah, J.-H., Becker, J.-M., & Ringle, C. M. (2019). How to specify, estimate, and validate higher-order constructs in PLS-SEM. *Australasian Marketing Journal (AMJ)*, *27*(3), 197–211. https://doi.org/10.1016/j.ausmj.2019.05.003

Schaufeli, W. B., & Salanova, M. (2007). Efficacy or inefficacy, that‘s the question: burnout and work engagement, and their relationships with efficacy beliefs. *Anxiety, Stress, and Coping*, *20*(2), 177–196. https://doi.org/10.1080/10615800701217878

Tarafdar, M., Tu, Q., & Ragu-Nathan, T. S. (2010). Impact of Technostress on End-User Satisfaction and Performance. *Journal of Management Information Systems*, *27*(3), 303–334.

Williams, S., & Cooper, C. L. (1998). Measuring Occupational Stress: Development of the Pressure Management Indicator. *Journal of Occupational Health Psychology*, *3*(4), 306–321.
